# High throughput analysis of MHC class I and class II diversity of Zambian indigenous cattle populations

**DOI:** 10.1111/tan.14976

**Published:** 2023-01-29

**Authors:** Isaac Silwamba, Deepali Vasoya, Martin Simuunza, Thomas Tzelos, King S. Nalubamba, Edgar Simulundu, Christina Vrettou, Geoffrey Mainda, Mick Watson, John Bwalya Muma, Timothy Connelley

**Affiliations:** ^1^ Department of Laboratory and Diagnostics Livestock Services Cooperative Society Lusaka Zambia; ^2^ Department of Disease Control, School of Veterinary Medicine University of Zambia Lusaka Zambia; ^3^ Centre for Tropical Livestock Genetics and Health (CTLGH), The Roslin Institute University of Edinburgh, Easter Bush Campus Roslin UK; ^4^ Department of Clinical Studies, School of Veterinary Medicine University of Zambia Lusaka Zambia; ^5^ Macha Research Trust Choma Zambia; ^6^ Department of Veterinary Services, Ministry of Fisheries and Livestock Central Veterinary Research Institute Lusaka Zambia; ^7^ The Roslin Institute University of Edinburgh, Easter Bush Campus Roslin UK

**Keywords:** cattle, high‐throughput sequencing, MHC

## Abstract

The classical MHC class I and class II molecules play key roles in determining the antigenic‐specificity of CD8+ and CD4+ T‐cell responses—as such characterisation of the repertoire of MHCI and MHCII allelic diversity is fundamental to our ability to understand, and potentially, exploit how genetic diversity influences the outcome of immune responses. Cattle remain one of the most economically livestock species, with particular importance to many small‐holder farmers in low‐and‐middle income countries (LMICs). However, our knowledge of MHC (BoLA) diversity in the indigenous breeds that form the mainstay of cattle populations in many LMICs remains very limited. In this study we develop a MiSeq‐based platform to enable the rapid analysis of BoLA‐DQA and BoLA‐DQB, and combine this with similar platforms to analyse BoLA‐I and BoLA‐DRB repertoires, to study a large cohort of cattle (~800 animals) representing the 3 major indigenous breeds (Angoni, Barotse, Tonga) in Zambia. The data presented confirms the capacity of this high‐throughput and high‐resolution approach to provide a full characterisation of the MHCI‐MHCII genotypes of cattle for which little previous MHC sequence data has been obtained. The cattle in Zambia were found to express a diverse range of MHCI, MHCII and extended MHCI‐MHCII haplotypes. The combined MHCI‐MHCII genotyping now possible opens new opportunities to rapidly expand our knowledge of MHC diversity in cattle that could find applications in a related translational disciplines such as vaccine development.

## 1 INTRODUCTION

The Major Histocompatibility Complex (MHC) locus contains many genes associated with antigen presentation, including the ‘classical’ MHCI and MHCII genes, which encode molecules that bind and present peptide fragments to CD8^+^ and CD4^+^ T‐cells, respectively. As key regulators of the antigen‐specificity of T‐cell immune responses, characterisation of the MHCI and MHCII genes is important for many aspects of immunology and associated disciplines such as vaccinology.

Although they have similar biological functions, the molecular structure and genetic organisation of MHCI and MHCII are fundamentally different. MHCI molecules are heterodimers composed of two polypeptide chains; the α chain (a.k.a. as the heavy chain – encoded within the MHC locus and containing 3 extra‐cellular α domains, a transmembrane region and a cytoplasmic tail) and β_2_‐microglobulin (a monomorphic protein that non‐covalently associates with the extra‐cellular component of the α chain and is not encoded within the MHC locus). MHCI molecules bind short antigenic peptide fragments of ~8–12 amino acids in the peptide‐binding groove (PBG) formed by the membrane‐distal α1 and α2 domains of the α chain (encoded by exon 2 and 3, respectively). In contrast, MHCII molecules are heterodimers composed of paired α and β chains, each of which has 2 extra‐cellular domains (α1/α2 and β1/β2, respectively), a transmembrane region and a cytoplasmic tail. There are multiple MHCII isotypes—for example, in humans, there are DR, DQ and DP MHCII molecules—and each of the α and β chains is encoded by genes situated within the MHC locus. The PBG of MHCII molecules is formed by the membrane‐distal α1 and β1 domains (encoded by exon 2 of the respective genes) and has a more open structure compared with the PBG of MHCI molecules, enabling the binding and presentation of longer peptides (~13–25 amino acids).

In most species, the ‘classical’ MHCI is polygenic, although the number of expressed MHCI genes varies between species; for example, in humans and mice there are 3 classical MHCI loci, whilst in Rhesus macaques (*Macaca mulatta*) upto 17 classical MHCI genes are expressed.^1^ For MHCII genes the number of loci varies—for example, in humans, there is a single DRA locus, 9 DRB loci (although only 3 encode for functional proteins), and 2 loci for each of DQA, DQB (although DQA‐2 and DQB‐2 are only expressed in Langerhans cells),^2^ DPA and DPB (although DPA‐2 and DPB‐2 are both pseudogenes). A key feature of the MHCI and most of the functional MHCII loci is extensive polymorphism. For example, in humans, each of the HLA‐A, ‐B and ‐C loci exhibit roughly equivalent, very high levels of polymorphism (7562, 9000 and 7513 alleles, respectively—IPD—http://www.ebi.ac.uk/ipd as of August 2022). The levels of polymorphism observed at the different functional MHCII loci varies; the DRB/DQB1/DPB1 loci exhibit high levels of polymorphism (4149, 2278 and 2067 alleles, respectively), the DQA1 and DPA1 moderate levels of polymorphism (483 and 455 alleles respectively) and the DRA a more limited degree of polymorphism (32 alleles, although only 5 protein variants). Most of this polymorphism is focused in the exons that encode the PBG domains (α1/α2 domains of MHCI and α1/β1 domains of MHCII), leading to variation in the PBG structure and, consequently, differences in the repertoires of peptides that can be bound and presented by different MHC molecules. The combination of polymorphism and polygenicity (of MHCI and the equivalent expression of multiple MHCII molecules achieved by the expression of multiple isotypes) generates a situation in which diverse sets of peptides from pathogens can be presented to T‐cells, constraining the capacity of pathogens to evade immune responses.

In cattle, the MHC locus and the repertoire of MHCI/MHCII (BoLA‐I/BoLA‐II) genes remain only partially characterised. Based on sequence analyses, it had been proposed that there were 6 classical BoLA‐I loci in cattle^3^, ^4^ although recent work using highly‐contiguous bovine genomes has suggested that this convention will need further refinement.^5^ However, the number of loci expressed varies between different haplotypes (ranging from 1 to 4), with different permutations of the six loci represented in different haplotypes. At present 138 different classical MHCI alleles are in the IPD database (https://www.ebi.ac.uk/ipd/mhc/group/BoLA—as of August 2022).

Cattle express both BoLA‐DQ and BoLA‐DR but no orthologue to human HLA‐DP.^6^ BoLA‐DRA is monogenic and although there are 3 BoLA‐DRB loci, only BoLA‐DRB3 is considered to be functional.^7^ As in other species, from a functional perspective, BoLA‐DRA is essentially mono‐morphic,^8^ whereas BoLA‐DRB3 shows high levels of polymorphism with 384 alleles recorded in the IPD database. The genetic organisation of BoLA‐DQ is more complex, with up to 5 putative DQA/DQB loci proposed based on sequence similarity of genes.^9^, ^10^ However, a maximum of 2 loci are expressed in any single haplotype. As with MHCI there is also variability in the number of loci expressed in different haplotypes—with approximately half of the haplotypes expressing single DQA/DQB genes and the other half expressing 2.^6^, ^11^ As a consequence of intra‐and inter‐haplotyping pairing of DQA and DQB molecules, individuals expressing 2 haplotypes bearing duplicated DQA/DQB loci theoretically have the capacity to generate up to 16 different DQ molecules.^12^, ^13^ As in humans, both BoLA‐DQA and DQB exhibit polymorphism—although the reported levels are more similar, with 76 and 91 alleles, respectively detailed in the IPD database.

Because of the polygenic nature of bovine MHCI, DQA and DQB, the generation of high‐resolution sequence data using Sanger sequencing has required costly and laborious sub‐cloning procedures, which have limited large‐scale studies of the allelic repertoires of these genes or the characterisation of complete MHCI/MHCII haplotypes. In recent studies, we have exploited the opportunities afforded by high‐throughput sequencing technologies to study the BoLA‐I and BoLA‐DRB repertoires of large cattle cohorts from the UK, Brazil, Kenya and Cameroon.^14^, ^15^

In this study, we expand on our previous work by developing an equivalent NGS approach to the analysis of BoLA‐DQA and ‐DQB. This was used in conjunction with the previously described BoLA‐I and ‐DRB typing to characterise the MHC haplotypes of a cohort of indigenous cattle from Zambia. As in many sub‐Saharan African countries, the livestock sector in Zambia contributes significantly to the agricultural GDP and accounts for employment of about 50% of the rural population.^16^ At present the national herd of Zambia is estimated to be 3,714,867 head of cattle, with the vast majority (93.5%) of these animals being kept by small‐holder farmers in herds of under 15 animals (85.8%). The main breeds of animals kept by small‐holder farmers were the local indigenous breeds: Angoni (26.1%), Tonga (21.4%) and Barotse (15.1%). In total 84.2% of small‐holder farmers keep either indigenous cattle or indigenous cross‐bred cattle, whilst only ~12% keep imported ‘exotic’ breeds such as Boran, Afrikander, Holstein‐Friesian and Jersey. At present there is no information available about the MHC repertoire of the main breeds constituting the Zambian national herd; in this study application of the NGS approach has provided sequence data on the combined MHCI‐MHCII repertoires on a large cohort (*n* = 627) of Tonga, Barotse and Angoni animals. The data generated confirms the capacity of the approach developed to enable a rapid, high‐resolution and high‐throughput characterisation of extended MHCI‐MHCII haplotypes in cattle and has demonstrated that indigenous Zambian cattle breeds have a highly diverse MHC repertoire. Information on the MHC repertoires of such cattle populations will be fundamental to ensuring that the immunogenetic diversity of indigenous populations can be considered in the development of new immunological interventions (e.g., vaccines) addressing endemic diseases.

## 2 MATERIALS AND METHODS

### 2.1 Sampling

**FIGURE 1:**
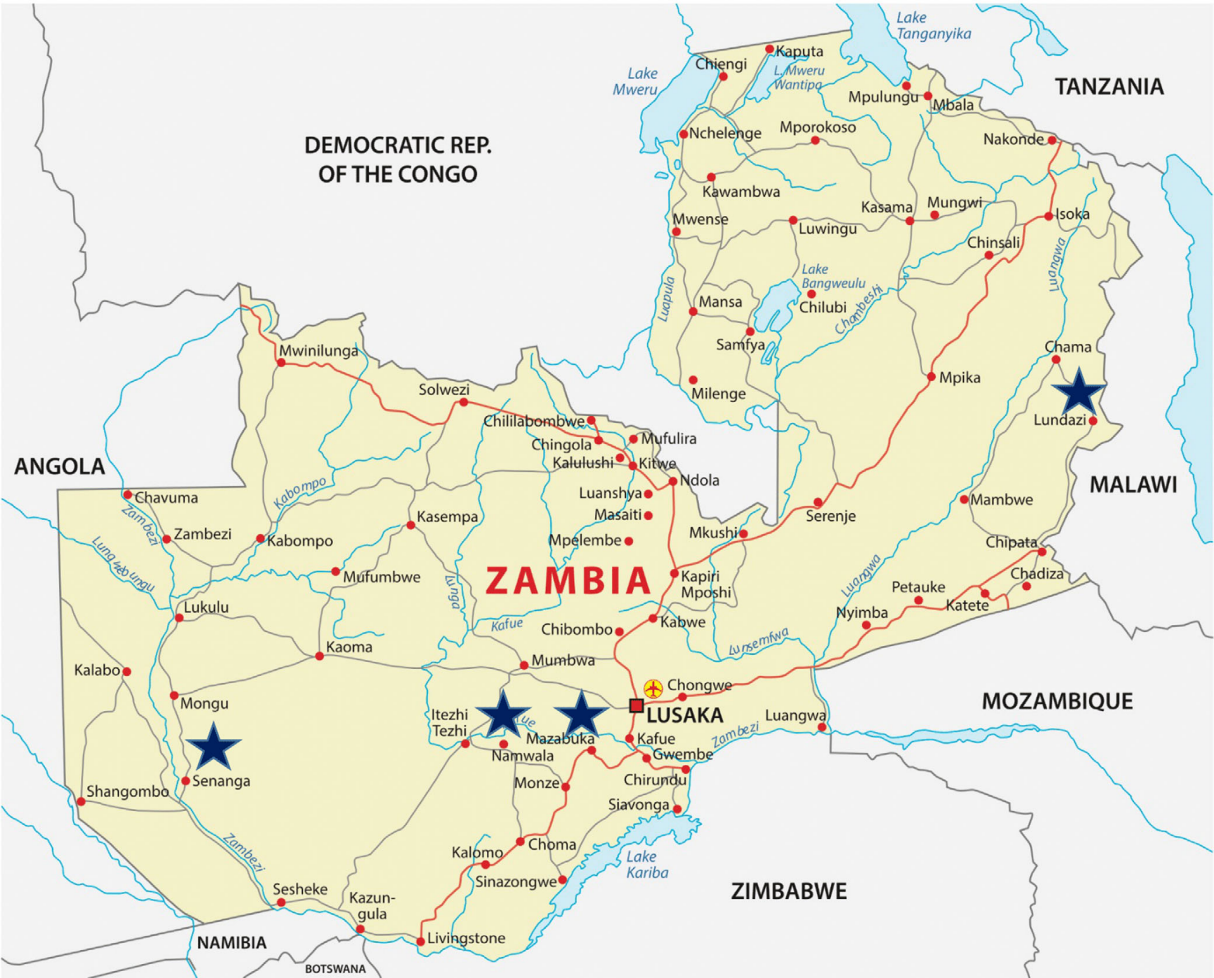
Map of Zambia showing the location of sampling sites. Samples were collected from farms in the following locations—Senanga (Western region), Namwala and Mazabuka (Southern Region) and Lundazi (Eastern Region). These locations are indicated by stars on the map.

Samples came from the Holstein‐Friesian animals at the University of Edinburgh's Langhill herd and cattle from multiple herds in different regions of Zambia, as shown in Figure 1 and Table 1. The work was approved by The Roslin Institute Animal Welfare and Ethics Review Panel and conducted under licence and in accordance with the UK Government Animal (Scientific Procedures) Act 1986 and the necessary permission from the relevant Zambian authorities (ERES Converge for ethics (Ref. No 2018‐Jan‐009) and PACRA for Nagoya compliance). Blood samples were collected by jugular venepuncture into EDTA vacutainers (BD Biosciences, Oxford, UK), and erythrocytes lysed by incubation in 5 × volume of red blood cell (RBC) lysis buffer (0.144M ammonium chloride/0.175M Tris pH 7.4) for 5 min at room temperature. The white blood cell (WBC) pellet was washed three times in PBS, total RNA extracted using Tri‐reagent (Sigma, Gillingham, UK) and cDNA synthesised using a Reverse Transcription Kit (Promega, Madison, WI), both according to the manufacturer's instructions.

**TABLE 1:** Summary of sample collection in UK and Zambia.

Breed (1)	Country (2)	Region (3)	District (4)	Sampling site (5)	Number of animals sampled (6)	Samples removed by QC (7)	Paired MHCI/MHCII data (8)	MHCI data only (9)
Holstein‐Friesian	UK	Roslin	Midlothian	Langhill farm	327	0	326	1
Angoni (*Bos indicus*)	Zambia	Eastern	Lundazi	Katube	99	0	65	34
				Mkanile	18	0	17	1
				Mwase	115	2	105	8
Tonga (Sanga)		Southern	Mazabuka	Research station	39	0	39	0
			Namwala	Lubanga Banga	50	2	43	5
				Moboola	39	0	33	6
				Ndema	89	6	46	37
				Settlement	49	3	14	32
				Shababwa	9	2	1	6
				Shimomba	54	0	51	3
Baila (Sanga)				Lubanga Banga	10	0	9	1
Barotse (Sanga)		Western	Senanga	Lui wanyau	59	1	52	6
				Nande	102	1	94	7
				Sibukali	68	1	58	9

*Note*: The table shows the breeds of cattle sampled (column 1), the country (2), region (3) and district (4) of origin and sampling sites (5) for all samples. The total numbers of animals sampled from which viable cDNA was isolated (6), samples removed during QC of the primary data (7), and animals for which paired MHCI/MHCII haplotypes (8) or MHCI haplotypes only (9) were identified are shown.

### 2.2 PCR amplification and library preparation

PCR amplification of MHCI genes was performed as described^14^ and DRB3 genes as described.^15^ DQA PCR amplification used a primer pair consisting of BoLA_DQAfor3 (STG GRR GTG AAG ACA TYG TG) and BoLA_DQArev2 (GAY TTG GRA AAC ACA GYC AC) and DQB PCR amplification used a primer pair consisting of BoLA_DQBfor1 (GGR CYG AGG GCA GAG ACT) and BoLA_DQBrev2 (GGR GAG ATG GTC ACT GTA GG); designed based on the bovine DQA and DQB exon1 and 3 nucleotide sequence data available in the IPD‐MHC database in July 2018. Series of BoLA‐DQ forward and reverse primers incorporating Illumina adaptors and multiplex identifier tags (MID) were obtained from IDT (Leuven, Belgium) to permit the generation of 192 amplicon pools from separate samples for each PCR that could be multi‐plexed in a single MiSeq run. BoLA‐DQA and ‐DQB amplification was conducted using the Phusion High‐Fidelity PCR Kit (New England Biolabs, Hitchin, UK) with 50 μL reactions composed of Phusion HF amplification buffer, 3% DMSO, 0.2 mM dNTPS, 25 pmol of for and rev primers, 1U Phusion Hot Start DNA polymerase and 1 μL of cDNA. Cycling conditions were 98°C for 30 s, 30 cycles of 98°C for 10 s, 61°C for 30 s, 72°C for 30 s and a final extension period of 72°C for 10 min. Following amplification, 5 μL of PCR product from each sample were pooled, run on a 1% agarose gel and bands of the appropriate size (~500 bp) were extracted and purified using the Qiagen Gel extraction kit (Qiagen, Manchester, UK). The extracted DNA was subsequently purified using Agencourt AMPure XP beads (Beckham Coulter, High Wycombe, UK) at a v/v ration of 1:1 beads to PCR product and quantified using 260/280 nm absorbance readings obtained from a Nanodrop spectrophotometer (Wilmington, DE, USA). The purified products from the MHCI For1/Rev2 and For3/Rev1, DQA, DQB and DRB3 pool were mixed at a ratio of 30%:25%:15%:15%:15%, respectively to generate the library submitted for sequencing. To increase diversity 10% PhiX was added to the libraries prior to sequencing.

### 2.3 Sequencing and bioinformatics analysis

Libraries were submitted to Edinburgh genomics, where after standard quality control procedures, they underwent 300 bp paired‐end sequencing on an Illumina MiSeq v3. Sequence reads were segregated based on MID combinations into (up to) 192 datasets, the raw data was assessed for quality (threshold score of >Q_28_), and paired‐end sequences were overlapped using FLASH.^17^ Data were then processed using a bioinformatics pipeline, essentially as described^15^ (available at https://github.com/deepalivasoya/MHCtyping). The raw sequencing data is available on ENA with the accession id PRJEB44287 (Holstein‐Friesian samples) and PRJEB55564 (Zambian samples). The downstream analysis of haplotypes and alleles, including calculating frequency, associations and linkage, was done using Perl and Python scripts (https://github.com/deepalivasoya/MHCtyping). Plots were created using ggplot2 in R; MHC haplotypes overlap, and diversity indices within/between breeds were calculated using R packages *divo* and *vegan*. Sequence data from previously defined MHC alleles were obtained from the IPD database (https://www.ebi.ac.uk/ipd/mhc/group/BoLA/).

### 2.4 Nomenclature of novel MHC sequences and haplotypes

The approach to nomenclature of MHCI alleles and haplotypes and DRB3 alleles is as described previously^14^, ^15^ and in accordance with the IPD‐MHC guidelines for nomenclature of non‐human MHC sequences generated from NGS data.^18^ An equivalent approach was used to name novel DQA and DQB alleles identified in this study. In brief, nucleotide sequences were translated to amino acid sequences and compared with the data in the IPD‐MHC database. If sequences showed ≤4 amino acid differences from an official designated sequence, then it was considered to be in the same allelic group as that sequence and was given a name to identify it as a novel allele. To avoid potential confusion with official IPD‐MHC (Maccari et al. 2018) naming, we used alphabet characters to name novel allele and synonymous variants (e.g., BoLA‐DQA*024:AA and BoLA‐DQA*012:05:AA). Sequences that showed >4 amino acid differences from any sequence in the IPD‐MHC database were considered to represent novel allelic groups and were assigned alpha‐numeric names based on the country of origin of the cattle they were first identified in (i.e., gb or zm for the UK and Zambia respectively) and a number followed by colon and a double digit number (e.g., BoLA‐DQB*gb1:01). MHCI haplotypes were defined by recurrent patterns of co‐segregation of alleles; to be assigned as a confirmed haplotype the pattern needed to be repeated in a minimum of 2 animals, if only identified in 1 animal the putative haplotype was prefixed with ‘un’ (i.e., unconfirmed). To provide a consistent system for the nomenclature of MHCII haplotypes (defined as a unique combination of DRB3, DQA and DQB alleles) we adopted the following approach; (i) pairs of DQA/DQB alleles were assigned a name composed of two numbers, with the first number representing the expression of a unique combination of DQA and DQB allelic groups and the second representing variants that contain a different allele of one or both of the DQA/DQB alleles (e.g., DQ30.1 = BoLA‐DQA*001:02|BoLA‐DQB*004:02 and DQ30.2 = BoLA‐DQA*001:AA|BoLA‐DQB*004:02); (ii) the BoLA‐DRB3 allele was abbreviated to a similar format—for example, BoLA‐DRB3*023:01 was abbreviated to DR23.1; (iii) the haplotype name was then formed from the concatenation of the assigned DR and DQ names—for example a haplotype expressing BoLA‐DRB3*023:01, BoLA‐DQA*001:02 and BoLA‐DQB*004:02 was defined as DR23.1|DQ30.1. For haplotypes expressing two pairs of DQ genes the haplotype type would include the names of two DQ pairs—for example, DR4.2|DQ18.3|DQ51.1. As with MHCI haplotypes, MHCII haplotype names were only assigned when the same combination of alleles was identified in multiple individuals.

## 3 RESULTS

### 3.1 Development of a bovine DQA and DQB allele sequencing protocol on the MiSeq platform

PCR protocols that permitted the amplification of exon 2 of bovine DQA and DQB alleles from cDNA were developed. The primers were designed to enable amplification of known bovine DQ alleles in the IPD database (based on in silico prediction) and enable unambiguous discrimination of the different DQA and DQB alleles after sequencing of the PCR amplicons on the MiSeq platform. The size of the DQA and DQB PCR amplicons were ~324 bp (36 bp of exon1, 264‐267 bp of exon2 and 21 bp of exon3) and ~319 bp (24 bp of exon1, 270 bp of exon2 and 26 bp of exon3) respectively. To provide preliminary validation of this protocol, DQA and DQB PCR amplification was conducted on cDNA samples from a cohort of 327 Holstein‐Friesian cattle and the amplicons subjected to sequencing analysis (performed in parallel with equivalent analysis of MHCI and DRB3 using the MiSeq protocol as described previously^15^). Bioinformatic analysis of the DQA and DQB sequencing data generated was performed using a pipeline based on that previously developed for the DRB3 data, which included filters to remove artefact reads that (i) had ambiguous base‐calls (N), (ii) were >9 bp different from the anticipated sequence size, (iii) were identified as PCR chimaeras and (iv) contained potential PCR/sequencing errors that led to the generation of 1 bp variants.

**FIGURE 2:**
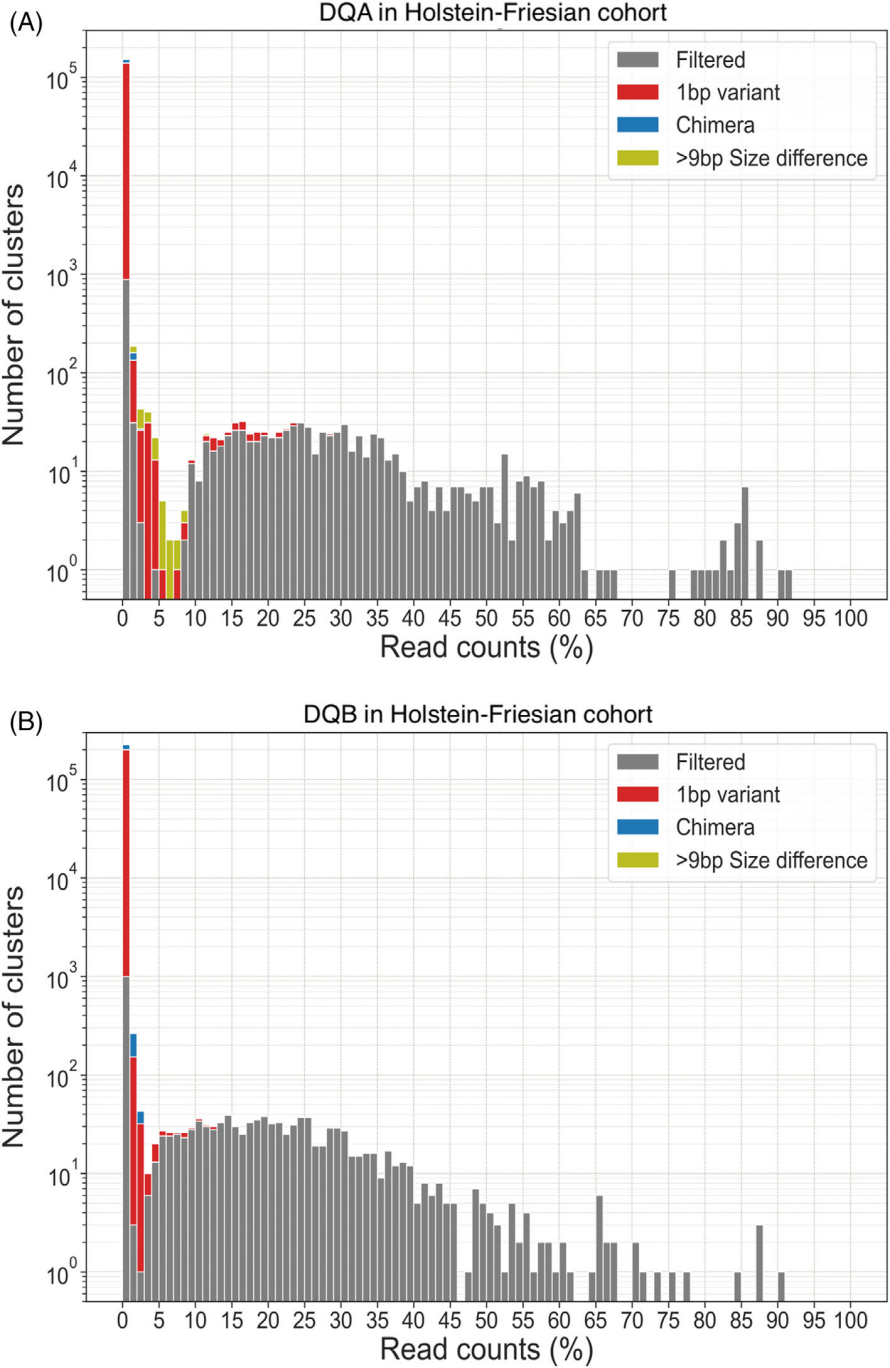
Histogram of BoLA‐DQ sequence clusters identified in a cohort of Holstein‐Friesian cattle. The figures show the total compiled number of clusters of (A) DQA and (B) DQB sequences identified in the Holstein‐Friesian cohort distributed by the % of the read count frequency they accounted for in their sample. Clusters that passed all of the other filters are shown in grey. Clusters that represented sequences that were 1 bp variants, chimaeras or were more than +/−9 bp difference from the anticipated length of the amplicon (and so were removed by the filters) are coloured red, blue and yellow respectively. There were no reads removed because of the inclusion of ambiguous base (‘N’) calls and so this is not shown in the legend.

As part of the bioinformatics pipeline, it was necessary to introduce a read count threshold filter to remove low‐frequency artefactual sequences generated as a consequence of non‐specific PCR and/or sequencing errors. To empirically define this threshold, the read frequency of each unique cluster of DQA and DQB reads (reads with 100% identity) in each animal was calculated, compiled, and plotted on a histogram (Figure 2). For DQA the data exhibited a profile with a dominant population of clusters extending from 9% to 68%, a small number of high‐frequency clusters (75%–90%) and a series of clusters with a frequency of <5%. The DQB profile was similar, but with an inflexion point in the number of clusters at 2%. Based on the profiles, the DQA and DQB thresholds were set at 7% and 2%, respectively. Subsequent analyses (based on patterns of co‐expressed MHCI and MHCII alleles – see below) demonstrated that across all animals in this cohort, only 1 DQA and 1 DQB cluster that were subsequently considered to be genuine sequences were below these thresholds, confirming their overall validity.

### 3.2 Analysis of MHCII haplotypes in a cohort of Holstein‐Friesian animals

Using the established DQA/DQB sequencing protocol, we sought (with the equivalent data for DRB3 – published previously^15^) to characterise the MHCII haplotypes in the cohort of 327 Holstein‐Friesian animals from which data had been derived. The summary of this data is provided in (Supplementary Data 1).

A total of 16 DQA and 20 DQB alleles were observed in this cohort of animals; of these, only 2 DQA and 1 DQB alleles were novel—BoLA‐DQA*024:AA, BoLA‐DQA*012:05:AA and BoLA‐DQB*gb1:01 (observed 17, 2 and 1 times, respectively—Supplementary Data 2). Recurrent co‐expression of DRB3/DQA/DQB alleles identified 20 different MHCII haplotypes (Table 2) with an additional 2 putative MHCII haplotypes implied from the presence of unique combinations of DRB3/DQA/DQB alleles in 1 animal (LH17‐703772—see Supplementary Data 1). To provide a consistent nomenclature for the MHCII haplotypes, we adopted a new notation system (as described in the materials and methods) in which the DRB3 and DQA/DQB pairs are defined by abbreviated terms, and the MHCII haplotype is a concatenation of these terms. Fourteen of the MHCII haplotypes matched previously published haplotypes.^12^, ^13^, ^19^, ^20^, ^21^, ^22^ However, for 4 of these haplotypes—DR5.1|DQ13.1|DQ4.1 (DH01A), DR10.1|DQ10.2|DQ6.1 (DH03A) and DR18.1|DQ13.1|DQ15.1 (DH18A) and DR6.1|DQ13.1|DQ16.1 (0601A)—our data identified the presence of additional alleles (Table 2). Conversely, the data indicated that our DQA primers failed to amplify alleles DQA*001:01 and DQA*014:01, which had been shown in previous studies to be expressed in DR1.1|DQ1.1 (DH24A) and DR14.1|DQ14.1 (DH27A) haplotypes (Table 2). Six of the MHCII haplotypes identified in this cohort of animals were novel—2 of these haplotypes were observed at relatively high frequency and represented the same DQA/DQB pairs combined with alternative DRB3 alleles (DR27.3|DQ1.2|DQ17.1 and DR7.1|DQ1.2|DQ17.1—combined frequency of ~9.3%), another 3 of the novel haplotypes were present at low frequencies (<0.5%—Table 2) and the final novel haplotype was only observed in a single animal (and so was considered to be an unconfirmed haplotype). In 4 of the novel haplotypes, there were ‘orphaned’ DQB alleles for which no partner DQA allele was identified; on the assumption that haplotypes express equal numbers of paired DQA and DQB genes, it appears that in these haplotypes not all DQA alleles were successfully amplified (Table 2).

**TABLE 2:** List of MHC class II haplotypes identified in this study.

Haplotype (1)	Previously designated haplotype^1^ (2)	BoLA‐DRB3 (3)	BoLA‐DQA (4)	BoLA‐DQB (5)	BoLA‐DQA (6)	BoLA‐DQB (7)	Observations in Holstein‐Friesian cattle		Observations in Zambian cattle	
							(8)	(9)	(10)	(11)
**DR10.1|DQ10.2|DQ6.1**	**DH03A**	*010:01	*010:04	*010:02:01	*021:02	*009:01	100	15.34%		
DR1.1|DQ1.1	DH24A	*001:01	*001:01	*001:01			95	14.57%	3	0.23%
DR11.1|DQ10.1|DQ5.1	DH22H	*011:01	*010:03	*010:02:01	*034:01	*014:02	94	14.42%		
DR15.1|DQ10.1|DQ4.1	DH16A	*015:01	*010:03	*010:02:01	*022:09	*013:01	85	13.04%	34	2.65%
DR14.1|DQ14.1	DH27A	*014:01:01	*014:01:01	*014:01			53	8.13%	59	4.60%
DR12.1|DQ12.1|DQ17.1	DH08A	*012:01	*012:05	*010:05	*022:08	*012:01	39	5.98%	16	1.25%
DR27.3|DQ1.2|DQ11.1		*027:03	?	*001:05	*028:02	*020:02	36	5.52%		
**DR18.1|DQ13.1|DQ15.1**	**DH18A**	*018:01	*025:01:02	*006:01	*022:08	*017:02	29	4.45%		
DR9.2|DQ3.1	0902B	*009:02/*049:01	*002:07	*018:03			27	4.14%	17	1.33%
DR7.1|DQ1.2|DQ11.1		*007:01	?	*001:05	*028:02	*020:02	25	3.83%	21	1.64%
DR16.1|DQ12.3|DQ4.1	1601C	*016:01	*012:06	*010:01	*022:09	*013:01	22	3.37%	1	0.08%
**DR6.1|DQ13.1|DQ16.1**	**0601A**	*006:01	*025:01:02	*006:01	*024:AA	*003:02	17	2.61%	1	0.08%
DR2.1|DQ2.1	DH07A	*002:01	*002:03	*002:01			11	1.69%	24	1.87%
**DR5.1|DQ13.1|DQ4.1**	**DH01A**	*005:01	*025:01:02	*006:01	*022:09	*013:01	5	0.77%		
DR5.1|DQ13.1|DQ9.1		*005:01	*025:01:02	*006:01	*022:08	*005:01	3	0.46%		
DR11.1|DQ10.2|DQ7.1	DH22E	*011:01	*010:04	*010:02:01	*021:02	*029:02	3	0.46%		
DR11.1|DQ12.3|DQ4.1		*011:01	*012:06	*010:01	*022:09	*013:01	3	0.46%		
DR20.1|DQ25.1		*020:01:01	?	*025:01			2	0.31%	33	2.57%
DR27.3|DQ12.2|DQ9.1	2703E	*027:03	*012:05:AA	*010:01	*022:08	*005:01	2	0.31%		
unDR18.1|DQ53.1|DQ52.1		*018:01	?	*018:03	*023:01	*gb1:01	1	0.15%		
DR30.1|DQ18.1		*030:01	*002:02	*004:02					76	5.92%
DR18.1|DQ37.1|DQ52.1		*018:01	*023:01	*003:02	?	*gb1:01			70	5.46%
DR28.1|DQ39.1|DQ15.1		*028:01/*028:04	*026:03	*006:01	*022:08	*017:02			67	5.22%
DR24.1|DQ25.2		*024:01/*024:07	?	*025:02					50	3.90%
DR13.AA|DQ38.1|DQ49.1		*013:AA	*026:02	*030:01	*029:01	*016:02			47	3.66%
DR21.1|DQ24.1		*021:01	*003:01	*015:01					43	3.35%
DR3.2|DQ19.2		*003:02:01	*002:07	*003:02					42	3.27%
DR16.1|DQ33.1|DQ46.1		*016:01	*012:05:AA	*041:01	*022:08	*009:01			41	3.20%
DR11.3|DQ21.1	1103A^1^	*011:03	*002:03	*036:02					38	2.96%
DR22.1|DQ41.3		*022:01	*027:04	*zm1:01					38	2.96%
DR19.1|DQ12.3|DQ47.1		*019:01	*012:06	*010:01	*022:08	*031:AA			31	2.42%
DR21.1|DQ27.3		*021:01	*003:01	*018:06:AA					29	2.26%
DR22.1|DQ40.4|DQ9.2		*022:01	*027:01:AA	*007:01	*022:08	*005:02			25	1.95%
DR44.1|DQ44.1		*044:01	?	*027:01					25	1.95%
DR28.1|DQ13.1|DQ15.1		*028:01/*028:04	*025:01:02	*006:01	*022:08	*017:02			22	1.71%
DR10.2|DQ26.1		*010:02	?	*026:01					20	1.56%
DR33.1|DQ30.2		*033:01	*001:AA	*004:02					19	1.48%
DR26.1|DQ10.4|DQ46.1		*026:01	*010:04	*010:09	*022:08	*009:01			18	1.40%
DR25.1|DQ36.1|DQ11.2		*025:01:01	*013:01	*032:01	*028:02:AA	*020:01			18	1.40%
DR25.1|DQ26.1		*025:01:01	?	*026:01					17	1.33%
DR13.AA|DQ40.1		*013:AA	*027:01:01	*007:AA					15	1.17%
DR27.4|DQ29.1|DQ11.3		*027:04	*004:01	*018:09	*028:AB	*020:AC			15	1.17%
DR28.2|DQ34.1		*028:02	*013:01	*018:02					15	1.17%
DR3.2|DQ25.1		*003:02:01	?	*025:01					11	0.86%
DR12.1|DQ12.1|DQ46.1		*012:01	*012:05	*010:05	*022:08	*009:01			10	0.78%
DR16.1|DQ10.2|DQ45.1		*016:01	*010:04	*010:02:01	*021:02	*017:02			10	0.78%
DR29.2|DQ42.1		*029:02	*030:01	*zm1:03					10	0.78%
DR20.3|DQ25.1		*020:03	?	*025:01					9	0.70%
DR16.1|DQ53.1|DQ46.1		*016:01	?	*041:01	*022:08	*009:01			9	0.70%
DR31.1|DQ32.1		*031:01	*008:03	*024:01					9	0.70%
DR23.1|DQ29.1|DQ11.3		*023:01	*004:01	*018:09	*028:AB	*020:AC			8	0.62%
DR13.2|DQ41.1	**1302A**	*013:02	*027:01:01	*zm1:01					8	0.62%
DR31.1|DQ38.1|DQ4.1		*031:01	*026:02	*030:01	*022:09	*013:01			8	0.62%
DR42.1|DQ27.2		*042:01	*003:01	*018:05					8	0.62%
DR20.5|DQ25.1		*020:05	?	*025:01					7	0.55%
DR48.2|DQ25.1		*048:02	?	*025:01					7	0.55%
DR10.2|DQ29.1|DQ11.3		*010:02	*004:01	*018:09	*028:AB	*020:AC			7	0.55%
DR32.2|DQ40.2		*032:02	*027:01:01	*007:AB					7	0.55%
DR10.1|DQ12.3|DQ4.1		*010:01	*012:06	*010:01	*022:09	*013:01			7	0.55%
DR39.1|DQ8.2		*039:01	*008:03	*008:02					7	0.55%
DR20.1.AA|DQ25.1		*020:01:AA	?	*025:01					6	0.47%
DR13.AA|DQ40.3		*013:AA	*027:01:01	*007:AC					6	0.47%
DR11.3|DQ19.1		*011:03	*002:03	*003:02					6	0.47%
DR27.5|DQ35.1		*027:05	*013:01	*019:01					6	0.47%
DR16.2|DQ1.1		*016:02	?	*001:01					6	0.47%
DR3.2|DQ3.1		*003:02:01	*002:07	*018:03					5	0.39%
DR3.1|DQ19.2		*003:01	*002:07	*003:02					5	0.39%
DR36.1|DQ34.1		*036:01	*013:01	*018:02					5	0.39%
DR5.1|DQ13.1|DQ15.1		*005:01	*025:01:02	*006:01	*022:08	*017:02			4	0.31%
DR4.1|DQ36.1|DQ11.2		*004:01	*013:01	*032:01	*028:02:AA	*020:01			4	0.31%
DR14.1|DQ12.3|DQ4.1		*014:01:01	*012:06	*010:01	*022:09	*013:01			4	0.31%
DR26.1|DQ33.2|DQ46.1		*026:01	*012:AA	*041:01	*022:08	*009:01			4	0.31%
DR36.1|DQ12.5|DQ4.2		*036:01	*012:05:AA	*010:02:01	*022:09	*013:02			4	0.31%
DR4.1|DQ18.2		*004:01	*002:AA	*004:01					4	0.31%
DR5.2|DQ41.2		*005:02	*027:03	*zm1:03					4	0.31%
DR10.2|DQ26.1|DQ11.2		*010:02	?	*026:01	*028:02:AA	*020:01			4	0.31%
DR16.1|DQ10.2|DQ9.1		*016:01	*010:04	*010:02:01	*022:08	*005:01			4	0.31%
DR4.2|DQ18.3|DQ51.1		*004:02	*002:AA	*004:02	*zm1:01	*020:AB			4	0.31%
DR41.1|DQ13.1|DQ16.1		*041:01	*025:01:02	*006:01	*024:AA	*003:02			3	0.23%
DR19.1|DQ36.1|DQ11.2		*019:01	*013:01	*032:01	*028:02:AA	*020:01			3	0.23%
DR30.1|DQ43.1		*030:01	?	*004:02					3	0.23%
DR5.2|DQ38.1|DQ48.1		*005:02	*026:02	*030:01	*029:01	*014:02			3	0.23%
DR9.1|DQ33.2|DQ46.1		*009:01	*012:AA	*041:01	*022:08	*009:01			3	0.23%
DR9.2|DQ28.1		*009:02/*049:01	*003:01	*033:02					3	0.23%
DR7.1|DQ1.2|DQ51.1		*007:01	?	*001:05	*zm1:01	*020:AB			3	0.23%
DR8.1|DQ8.1|DQ11.2		*008:01	*008:01	*008:01	*028:02:AA	*020:01			3	0.23%
DR15.1|DQ10.3|DQ4.1		*015:01	*010:03	*010:01	*022:09	*013:01			3	0.23%
DR20.AB|DQ31.1		*020:AB	*008:01	*022:01					3	0.23%
DR27.3|DQ1.1		*027:03	?	*001:01					3	0.23%
DR10.1|DQ27.3		*010:01	*003:01	*018:06:AA					2	0.16%
DR16.1|DQ12.3|DQ47.1		*016:01	*012:06	*010:01	*022:08	*031:AA			2	0.16%
DR16.1|DQ12.2|DQ9.1		*016:01	*012:05:AA	*010:01	*022:08	*005:01			2	0.16%
DR48.2|DQ8.1|DQ11.2		*048:02	*008:01	*008:01	*028:02:AA	*020:01			2	0.16%
DR23.1|DQ30.1		*023:01	*001:02	*004:02					2	0.16%
DR15.1|DQ10.1|DQ50.1		*015:01	*010:03	*010:02:01	*034:01	*029:03			2	0.16%
DR35.1|DQ23.1		*035:01	*002:08	*022:01					2	0.16%
unDR21.1|DQ25.1		*021:01	?	*025:01					1	0.08%
unDR24.1|DQ3.1		*024:01/*024:07	*002:07	*018:03					1	0.08%
unDR17.3|DQ20.1		*017:03	*002:03	*023:01					1	0.08%
unDR7.1|DQ12.3|DQ47.1		*007:01	*012:06	*010:01	*022:08	*031:AA			1	0.08%
unDR15.1|DQ26.1		*015:01	?	*026:01					1	0.08%
unDR22.1|DQ40.5		*022:01	*027:04	*007:AC					1	0.08%
unDR27.3|DQ35.1		*027:03	*013:01	*019:01					1	0.08%
unDR16.1|DQ10.2|DQ17.1		*016:01	*010:04	*010:02:01	*022:08	*012:01			1	0.08%
unDR22.1|DQ8.1|DQ11.2		*022:01	*008:01	*008:01	*028:02:AA	*020:01			1	0.08%
unDR10.1|DQ12.2|DQ45.1		*010:01	*012:05:AA	*010:01	*021:02	*017:02			1	0.08%
unDR19.1|DQ12.3|DQ46.1		*019:01	*012:06	*010:01	*022:08	*009:01			1	0.08%
unDR27.7|DQ27.1		*027:07	*003:01	*018:03					1	0.08%
unDR39.1|DQ1.1		*039:01	?	*001:01					1	0.08%
unDR7.1|DQ14.1		*007:01	?	*014:01					1	0.08%

*Note*: The MHCII haplotypes are shown using the nomenclature system adopted in this study (column 1—see Materials and Methods for further details) and where relevant using the names given in previous studies (Lewin et al., 1999; Glass et al., 2000; Park et al., 2004; Staska et al., 2005; Miyasaka et al., 2012) (column 2). The DRB3, DQA and DQB alleles constituting the haplotypes are detailed in columns 3–7. Columns 8–11 shown how often each haplotype was identified (both in absolute count of observations and as a frequency) in the Holstein‐Friesian and Zambian cohorts. Haplotypes which had been previously defined in the literature, but for which our data identifies the expression of additional genes are highlighted in bold (with the additional DQA/DQB alleles identified also highlighted in bold). We have assumed that haplotype DR6.1|DQ13.1|DQ16.1 is the same as the previously reported haplotype 0601A although in the previous publication only the DRB3 and 1 DQB allele were described. Haplotypes for which the DQA primers appear to not amplify DQA alleles are shown in underlined script; where previous data has identified the DQA allele which is presumed to have not been amplified it is shown on a grey background in column 4, for novel haplotypes where the identity of the missing alleles is unknown, a question mark is used to represent the putatively missing DQA allele. Putative haplotypes which are observed in only single animal are designated as unconfirmed and are indicated by prefix of ‘un’ in their names.

Using the combined DQA/DQB and DRB3 sequencing protocols, we were able to characterise the MHCII haplotypes of 326/327 of the animals in this cohort: 291 animals were identified as heterozygous for the MHCII haplotype, 34 were homozygous, and 1 individual expressed 3 MHCII haplotypes (this animal was previously found also to express 3 MHCI haplotypes—suggesting it may be a twin with the expression of >2 MHC haplotypes because of blood chimaerism^15^). The last animal expressed a unique combination of DRB3, DQA and DQB alleles, and it was not possible to unambiguously resolve these into 2 MHCII haplotypes (LH17‐703772—see Supplementary Data 1). Thus, despite the partial deficiency of the DQA primers in not amplifying all alleles, the summation of the data confirmed the capacity of the developed MiSeq‐based sequencing protocol to rapidly characterise the MHCII haplotypes for a large group of cattle.

### 3.3 Analysis of MHC repertoires in Zambian cattle populations

Having verified the capacity to genotype MHCII in Holstein‐Friesian cattle, we applied the combined MHCI/MHCII genotyping to a cohort of cattle from Zambian breeds for which the diversity of expressed MHC has not been previously studied. Viable cDNA was generated from a total of 800 animals from 14 different sites in 3 different regions, representing 4 different indigenous breeds (Angoni, Tonga, Baila and Barotse) as summarised in Table 1.

Within this sample set, there was insufficient read data from 16 animals to reliably determine the MHCI haplotypes and 125 animals for MHCII haplotypes. MHCI data for an additional 2 animals suggested substantial cross‐sample contamination, whilst for an additional 48 animals, the combinations of DRB3, DQA and DQB alleles identified could not be unambiguously resolved into MHCII haplotypes. These data were removed from the subsequent analysis and consequently, the final Zambian dataset analysed comprised paired MHCI/MHCII data from a cohort of 627 animals and MHCI‐only data from an additional 155 animals (Supplementary Data 3). This included paired MHCI/MHCII (and MHCI only) data from 204 (22) Barotse, 227 (89) Tonga, 187 (43) Angoni, and 9 (1) Baila cattle, respectively (Table 1).

### 3.4 MHCI in Zambian cattle

**FIGURE 3:**
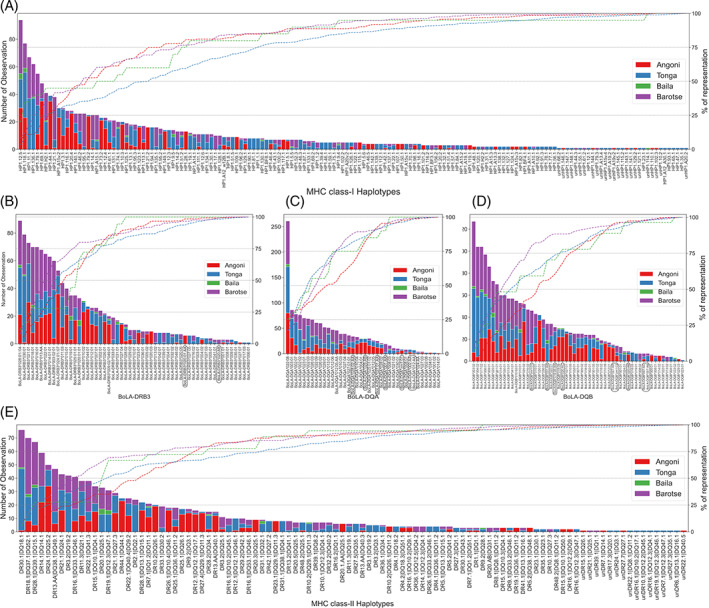
Frequency of (A) MHCI haplotypes, (B) DRB3, (C) DQA, (D) DQB alleles and, (E) MHCII haplotypes in the Zambian cohort. The number of times each MHCI and MHCII haplotype and BoLA‐II allele was observed and the cumulative percentage (represented by the dashed lines) of observations are described in the left and right vertical axis respectively. The alleles/haplotypes are arranged in descending order of observation frequency on the horizontal axis. Different breeds are shown in different colours as detailed in the legend. The newly identified DRB3, DQA and DQB alleles are highlighted in rectangles.

A total of 260 different MHCI alleles were identified, of which 63 were novel. The novel alleles (Supplementary Data 2) include representatives of 16 new allelic groups and 47 new members of previously identified allelic groups (of which 5 have only synonymous substitutions of previously known alleles). By use of recurrent co‐expression (or co‐segregation) of alleles, 158 different MHCI haplotypes were defined in this cohort, of which 78 were previously known haplotypes,^14^, ^15^ 56 were novel, and 24 were putative new haplotypes (i.e., observed only in single individuals and so assigned as unconfirmed haplotypes). Details of the novel and putative MHCI haplotypes are provided in Table 3. Of the 56 novel haplotypes, the majority (37%–66%) were composed of a combination of previously identified and novel alleles, over a quarter (17%–30.5%) were composed of novel combinations of previously known alleles, and only 2 (3.5%) haplotypes were composed of novel alleles only. There is a clear hierarchy in the frequency of the MHCI haplotypes, with the 10 most frequent accounting for ~34% of observed haplotypes and a large number of haplotypes found at low frequencies (ranging in a continuous spectrum from 1.85% to 0.06%—Figure 3A). Consistent with previous data, the MHCI haplotypes identified in this cohort varied in both the numbers of genes expressed (ranging from 1 to 4—Table 3) and the relative levels at which these genes appeared to be expressed (Supplementary Data 4).

**TABLE 3:** List of 80 novel MHCI haplotypes identified from Zambian cattle.

Haplotype (1)	Allele 1 (BoLA‐) (2)	Allele 2 (BoLA‐) (3)	Allele 3 (BoLA‐) (4)	Allele 4 (BoLA‐) (5)	Total Observations (5)	% observations (6)
HP1.100.2	4*063:01	2*032:AAN	3*078:01/3*078:02		7	0.43
HP1.101.2	2*089:AB	4*094:01	3*004:04/3*083:01		3	0.19
HP1.102.2	2*075:01	3*004:04/3*083:01	4*095:01		5	0.31
HP1.111.1	1*028:AC	2*054:AA:01			11	0.68
HP1.112.1	3*068:01	2*032:02			4	0.25
HP1.113.1	1*042:AA	MHCI*cm22:01			17	1.05
HP1.115.1	3*038:AB	3*004:04/3*083:01	6*090:AC		5	0.31
HP1.116.1	MHCI*zm1:01	MHCI*cm41:05	3*004:02:AC		28	1.73
HP1.116.2	MHCI*zm1:02	MHCI*cm41:05	3*004:02:AC		3	0.19
HP1.117.1	4*063:01	2*057:AB	3*078:01/3*078:02		7	0.43
HP1.118.1	2*069:AA	1*067:AA			77	4.75
HP1.119.1	2*022:AA	MHCI*cm24:01			3	0.19
HP1.12.5	3*073:AE	3*004:AB	MHCI*cm1:02	6*090:01	94	5.8
HP1.12.7	3*073:AD	3*004:AB:01	6*090:01		11	0.68
HP1.120.1	MHCI*cm4:01	2*016:01:AA	3*087:01:AA		2	0.12
HP1.122.1	MHCI*zm22:01	MHCI*zm18:01	6*040:01	MHCI*zm16:01	5	0.31
HP1.123.1	3*050:AC	3*004:04/3*083:01	2*025:01	6*040:01	17	1.05
HP1.124.1	3*050:AF	3*037:01			2	0.12
HP1.125.1	2*030:01:AA	MHCI*zm19:01	MHCI*cm1:01		19	1.17
HP1.126.1	MHCI*zm23:01	2*048:01	3*078:01/3*078:02		5	0.31
HP1.127.1	MHCI*zm21:01	MHCI*cm13:01			2	0.12
HP1.128.1	MHCI*zm21:01	2*048:01			10	0.62
HP1.129.1	2*047:AB	1*097:01:AA			8	0.49
HP1.130.1	2*070:01	5*072:01			8	0.49
HP1.131.1	2*071:AA	MHCI*br28:01	6*090:AA		21	1.3
HP1.132.1	BoLA‐3*011:01	BoLA‐2*048:01	BoLA‐3*078:01/BoLA‐3*078:02		2	0.12
HP1.133.1	MHCI*cm4:08	2*048:01	3*004:02/3*053:01/3*081:01		6	0.37
HP1.134.1	MHCI*br32:01	MHCI*zm7:01			11	0.68
HP1.135.1	5*072:01	6*090:AB			16	0.99
HP1.136.1	MHCI*br30:01	4*094:01:AA	3*098:01N		62	3.82
HP1.137.1	MHCI*br7:01	MHCI*zm8:01	2*006:01/2*006:02/2*006:03	3*078:01/3*078:02	4	0.25
HP1.138.1	MHCI*cm9:02	4*094:AB	3*004:04/3*083:01	MHCI*cm1:03	2	0.12
HP1.139.1	MHCI*cm38:01	2*047:01	3*078:01/3*078:02		4	0.25
HP1.14.2	MHCI*cm10:01	3*004:02/3*053:01/3*081:01	MHCI*cm13:01		13	0.8
HP1.140.1	3*050:AE	2*047:01	3*004:04/3*083:01		26	1.6
HP1.142.1	MHCI*zm3:01	3*017:AD	2*055:01	3*033:01N	4	0.25
HP1.149.1	1*009:AD	6*090:01			14	0.86
HP1.156.2	3*050:AE	3*004:04/3*083:01			3	0.19
HP1.20.4	MHCI*cm8:04	MHCI*cm40:02			28	1.73
HP1.21.2	MHCI*cm9:03	3*017:AB	3*004:02:AB	6*092:AA	16	0.99
HP1.46.4	MHCI*cm4:08	2*008:AA	3*004:02/3*053:01/3*081:01	6*091:01	7	0.43
HP1.46.5	MHCI*cm4:08	2*008:AA	3*004:04/3*083:01	6*091:01	4	0.25
HP1.5.5	1*029:AC:01	2*030:01:AA	MHCI*cm1:01		7	0.43
HP1.5.6	1*029:AE	2*030:01:AA	MHCI*cm1:01		4	0.25
HP1.51.2	2*022:AE	1*074:01			13	0.8
HP1.52.3	MHCI*gb20:01	3*004:02/3*053:01/3*081:01	2*016:02/2*016:03	6*091:01	7	0.43
HP1.69.2	3*080:AA	2*057:01	3*033:01N	6*090:01	6	0.37
HP1.79.2	MHCI*br7:01	MHCI*br15:02			25	1.54
HP1.95.2	MHCI*br1:02	3*004:03			15	0.93
HP1.95.3	MHCI*br1:03	3*004:03			18	1.11
HP1.96.2	MHCI*br9:02	2*060:01	6*093:01		8	0.49
HP1.A13.3	1*031:02	2*032:ACN			2	0.12
HP1.A15.3	1*009:AC	4*024:01:01:01/4*024:01:01:02	2*025:01		2	0.12
HP1.A15v.3	1*009:AA	4*024:01:01:01/4*024:01:01:02	6*040:01	2*025:AA	2	0.12
HP1.A20v.2	3*027:02	2*026:04			5	0.31
HP1.H2.2	5*039:AA				2	0.12
unHP1.10.2	3*038:01:AA	2*096:01:AB	3*004:02/3*053:01/3*081:01		1	0.06
unHP1.102.3	2*075:01	3*004:04/3*083:01	4*095:AB		1	0.06
unHP1.110.1	MHCI*zm5:01	2*008:02/2*008:03	2*006:01/2*006:02/2*006:03		1	0.06
unHP1.114.1	MHCI*zm9:01	2*008:AC			1	0.06
unHP1.115.2	3*038:AD	3*004:04/3*083:01	6*090:01		1	0.06
unHP1.121.1	MHCI*zm29:01	MHCI*zm28:01			1	0.06
unHP1.129.2	2*047:AB	1*097:01			1	0.06
unHP1.141.1	MHCI*br29:01	2*032:AAN	2*008:AB		1	0.06
unHP1.143.1	3*050:AE	3*004:04/3*083:01	MHCI*cm9:03		1	0.06
unHP1.144.1	MHCI*zm2:01	2*048:01	3*078:01/3*078:02		1	0.06
unHP1.145.1	2*079:01	6*091:01	MHCI*gb5:01		1	0.06
unHP1.146.1	1*021:01	2*054:AA:01			1	0.06
unHP1.147.1	2*005:01	3*052:01	3*004:04/3*083:01		1	0.06
unHP1.148.1	MHCI*zm11:01	4*077:01	3*033:ABN		1	0.06
unHP1.44.3	MHCI*ke1:03	3*004:AD	6*090:01		1	0.06
unHP1.55.1	MHCI*cm10:01	3*004:AA:01	MHCI*ke5:01		1	0.06
unHP1.61.2	3*066:AB	3*004:03			1	0.06
unHP1.66.2	2*005:AA	3*087:01:AA	MHCI*ke8:04		1	0.06
unHP1.79.3	MHCI*br7:02	MHCI*br15:01			1	0.06
unHP1.A13.4	1*031:AA	2*032:01N			1	0.06
unHP1.A15v.4	1*009:AA	4*024:01:01:01/4*024:01:01:02	2*025:01	6*040:AE	1	0.06
unHP1.A19.2	6*014:01	2*016:01			1	0.06
unHP1.A19v.2	6*014:02:AA	2*016:01	MHCI*cm36:01		1	0.06
unHP1.A20.2	3*027:AA	2*026:04			1	0.06

*Note*: For each haplotype (column 1) the names of the constituent alleles (columns 2–5), the number of observations (column 5) and their frequency (column 6) in the Zambian cohort are detailed. Novel alleles identified in this data are highlighted in bold script. Alleles shown in underlined script consistently had low read frequency in animals carrying the haplotype and were not observed in a subset of these animals (presumed to be because of presence below the threshold of 0.2% of reads). Putative haplotypes that were observed in single animals are indicated by prefix of ‘un’ in their names.

### 3.5 MHCII in Zambian cattle

A total of 55 DRB3, 38 DQA and 55 DQB alleles were identified in the Zambian cohort, of which only a small number (2, 7 and 9, respectively) were novel (Supplementary Data 2). For DRB3, both novel alleles were variants of BoLA‐DRB3*020 (BoLA‐DRB3*020:AB and BoLA‐DRB3*020:01:AA), and for DQA and DQB, most of the new alleles were new members of previously defined allelic groups (only 1 novel DQA and DQB allelic group were identified—BoLA‐DQA*zm1 and BoLA‐DQB*zm1 respectively). The relative frequency of the DRB3 and DQB alleles showed profiles similar to that for the MHCI haplotypes, with continuous ranges from ~7% to ~0.08% and 0.05%, respectively (Figure 3B,D). In contrast, the profile for DQA was strikingly different, with a single allele—BoLA‐DQA*022:08 having a frequency of 18.8%, distinct from the distribution of the other alleles, which formed a continuous range from 5.8% to 0.14% (Figure 3C).

A total of 100 different MHCII haplotypes were identified in the cohort (Table 2). Ten of these haplotypes had been previously described (and 9 were also identified in the Holstein‐Friesian cohort), 76 were novel, and 14 were putative novel haplotypes (i.e. observed in single individuals and so not confirmed). From the DQA/DQB data, 74 unique DQA/DQB pairs were identified, which were arranged into 69 unique DQ haplotypes (33 of which contained duplicated DQ pairs and 36 contained single DQ pairs). Different permutations of DQ haplotypes and DRB3 alleles (i.e., DR haplotypes) generated the final total of 100 MHCII haplotypes. Frequency distribution of the complete MHCII haplotypes showed a similar profile as for the MHCI (Figure 3E), with the top 10 most frequent haplotypes accounting for 41.7% of the observed haplotypes, whilst the 10 least frequent haplotypes accounted for <1%. As in the Holstein‐Friesian cohort, there were a number (*n* = 10) of inferred DQA/DQB pairs for which DQB but not DQA genes were identified; these ‘orphan’ DQA/DQB pairs contributed to a total of 22 MHCII haplotypes.

### 3.6 Diversification of MHC haplotypes by allele modification and recombination

**FIGURE 4:**
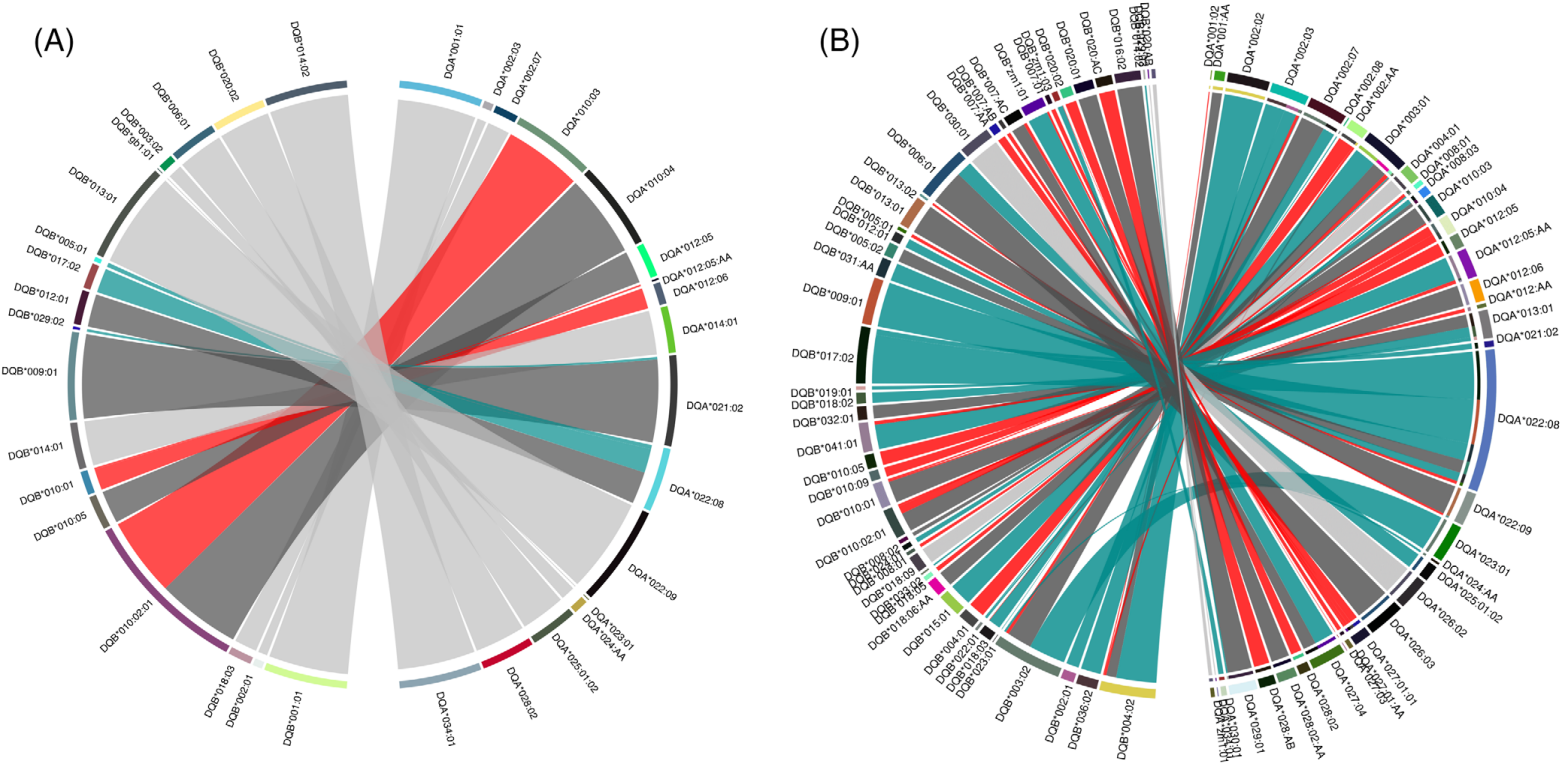
Chord plots showing paired DQA and DQB alleles in the (A) Holstein‐Friesian and (B) Zambian cohorts. Arcs join DQA and DQB alleles co‐expressed within DQ loci. The thickness of each arc is proportional to the frequency at which each DQA/DQB allele pair are observed within the dataset. The colour of the arcs represents if they are DQA and DQB alleles expressed exclusively together in a ‘one‐to‐one’ association (light grey arc) or if they are part of a ‘group’ of related DQA/DQB pairs. It is not possible from the data to define the historical relationship between DQA/DQB pairs within these groups (i.e., which was the ‘parent’ DQA/DQB pair, and which were derived from the ‘parent’ via MU/GC and/or recombination events); the figure is intended only to provide a representation of the contribution of putative recombination and MU/GC events in diversifying the repertoire of DQA/DQB pairs. Therefore in each ‘group’ the DQA/DQB pair observed at the highest frequency was assigned as the ‘index’ DQA/DQB allele pair (dark grey arc); pairs that included either a DQA or DQB allele that was a different member of the same allelic subgroup as that observed in the ‘index’ pair were represented as a putative MU/GC event (red arc); pairs that included the same DQA or DQB allele co‐expressed with a DQB or DQA allele from a different allelic subgroup were identified as a putative recombinations (cyan arcs). The DQA/DQB pairs in which no DQA allele was identified have not included; the figure represents data from a total of 20 and 65 DQA/DQB pairs in the Holstein‐Friesian and Zambian cohorts respectively.

**FIGURE 5:**
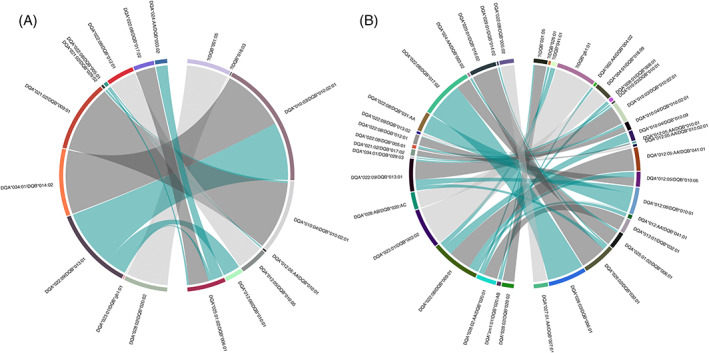
Chord plots showing co‐expressed DQ loci in MHCII haplotypes bearing duplicated DQ loci in the (A) Holstein‐Friesian and (B) Zambian cohorts. Arcs join DQ loci which are paired in MHCII haplotypes bearing duplicated DQ loci. The thickness of each arc is proportional to the frequency at which each paired DQ loci are observed within the dataset. The colour of the arcs represents if they are DQ loci are expressed exclusively together in a ‘one‐to‐one’ association (light grey arc) or if they are part of a ‘group’ of related paired DQ loci. It is not possible from the data to define the historical relationship between paired DQ loci within these groups (i.e., which was the ‘parent’ DQ haplotype and which were derived from the ‘parent’ via recombination); the figure is intended only to provide a representation of the contribution of putative recombination events in diversifying the repertoire of DQ haplotypes by forming different combination of DQ loci. Therefore in each ‘group’ one DQ haplotype was assigned as the ‘index’ (dark grey arc); and other DQ haplotypes which included one of the same DQ loci (i.e., paired DQA/DQB alleles) was assigned as a putative recombination (cyan arcs). The figure represents data from a total of 13 and 33 duplicated DQ haplotypes in the Holstein‐Friesian and Zambian cohorts respectively.

The two principal mechanisms by which the repertoires of MHC haplotypes are diversified are through (i) allele modification (by mutation or gene conversion) and (ii) formation of different permutations of alleles (and/or loci) through recombination.^23^, ^24^ Casual observation of our data indicates evidence of both occurring in the cohorts of animals included in this study. Although the data presented here is not of the type or structure conventionally used to study MHC mutation/gene conversion (MU/GC) or recombination events, we sought to provide some estimates of the role these two mechanisms had in diversifying the MHC repertoires characterised herein by using proxies for MU/GC (recurrence of different allelic variants from the same BoLA group with the same adjacent BoLA gene/region—e.g., BoLA‐DQA*010:03 and 010:04 with DQB*010:02:01 in Holstein‐Friesians as shown in Figure 4A) and recombination (different permutation of a BoLA region/BoLA allele with an adjacent BoLA region/BoLA allele from different group—for example, DQ pair DQA*010:03|DQB*010:02:01 found in duplicated DQ haplotypes with both DQA*034:01|DQB*014:02 and DQA*022:09|DQB*013:01 in Holstein‐Friesians as shown in Figure 5A) events. Based on the genomic organisation of the bovine MHC locus (Supplementary Data 5) we applied this approach sequentially, looking at: (i) DQA/DQB genes within DQ pairs, (ii) paired DQ loci in DQ duplicated haplotypes, (iii) DQ/DR haplotypes and (iv) MHCI/DR haplotypes.

**FIGURE 6:**
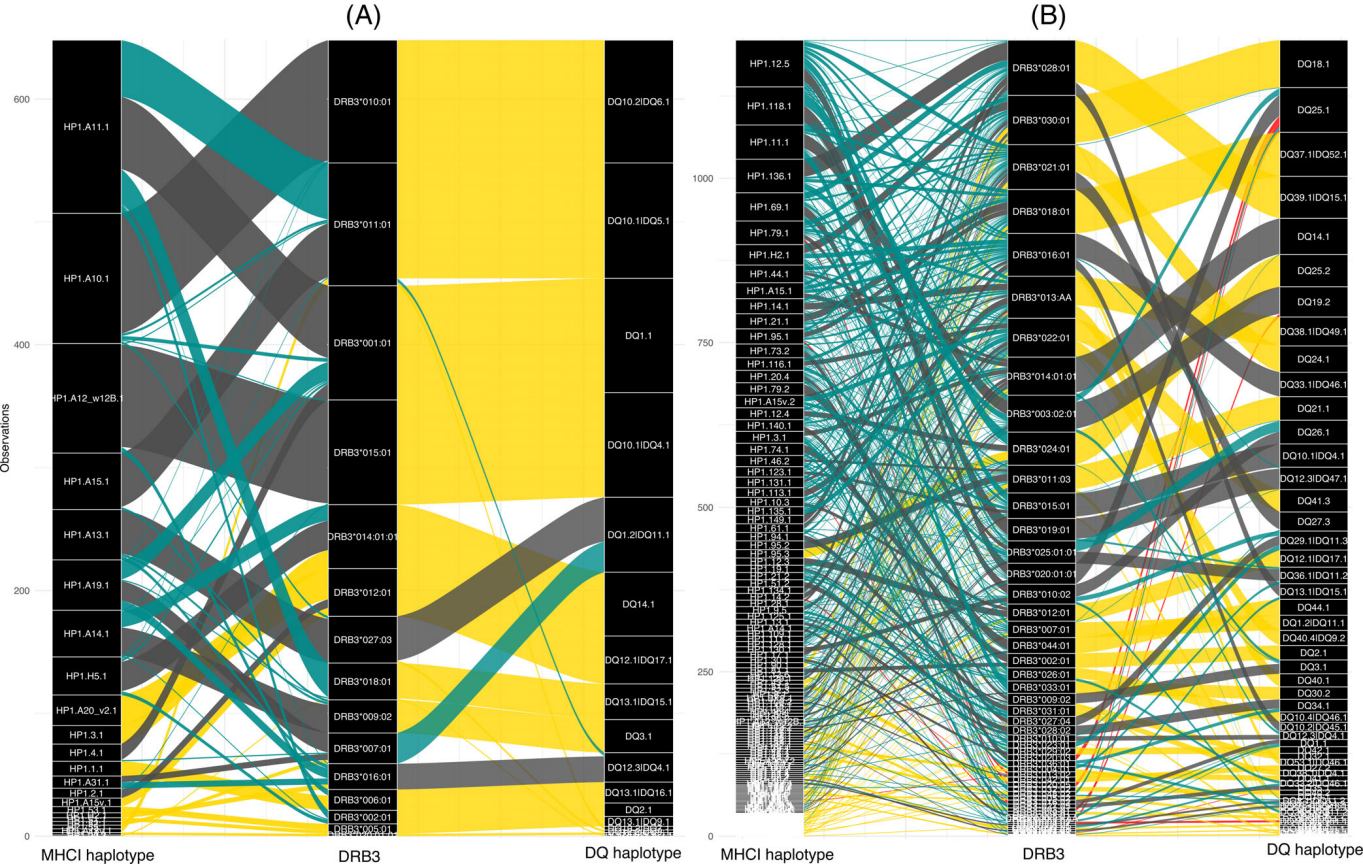
Alluvial diagram showing the association between expressed MHCI, DR and DQ haplotypes in (A) Holstein‐Friesian and (B) Zambian cohorts. Lines connect MHCI and DR (left side of panels) and DR and DQ (right side of panels) haplotypes which co‐segregate in extended MHCI‐MHCII haplotypes. The width of the lines represented the frequency at which these MHCI/DR and DR/DQ associations were observed within the cohorts. The colour of the lines represents if the associations are exclusive ‘one‐to‐one’ associations (yellow lines) or if the associations are part of a ‘group’ of related MHCI/DR or DR/DQ pairings. As it is not possible to define from the data the historical relationship between pairings in the same ‘group’, the most frequently observed pairing in each ‘group’ is designated as the ‘index’ (dark grey line) and other pairings were assigned as either putative recombination (cyan lines) or MU/GC (red lines) events. The figure is based on 50 and 457 extended MHCI‐MHCII extended haplotypes in the in the Holstein‐Friesian and Zambian cohorts respectively.

(i) Paired DQA/DQB alleles in DQ loci: In the Holstein Friesian cohort, a total of 20 DQA/DQB pairs (excluding incomplete DQA/DQB pairs with no DQA gene) were identified. Ten of these (50%) represented simple ‘one‐to‐one’ relationships between single DQA and DQB alleles (Figure 4A – light grey arcs). The remaining 10 DQ pairs belonged to ‘groups’ where there was an indication of diversification, with 3 DQA/DQB pairs appearing to arise from MU/GC (15% of DQA/DQB pairs; Figure 4A – red arcs) and 3 arising from putative recombination (Figure 4A – cyan arcs) events. In the Zambian cohort (Figure 4B), of the 65 unique DQA/DQB pairs identified, only 4 (6.2%) represented ‘one‐to‐one’ relationships, with the majority belonging to groups where there was an indication of mutation (24 DQA/DQB pairs arising from MU/GC—36.9%) and/or recombination (24%–36.9%). Notably, in both cohorts, DQA*022:08 appeared to be involved in a disproportionate number of recombination events—forming DQA/DQB pairs with 3 and 6 DQB alleles from different DQB groups in the Holstein‐Friesian and Zambian cohorts, respectively. (ii) paired DQ loci in duplicated DQ haplotypes: in the Holstein‐Friesian and Zambian cohorts, there were 13 and 34 DQ haplotypes containing duplicated DQ pairs, respectively. Of these, approximately equal proportions appear to have arisen because of recombination occurring between the DQ pairs—7 (53.8%) and 20 (58.8%) in the Holstein‐Friesian and Zambian cohorts, respectively (Figure 5A,B). Notably, in the Zambian cohort, 9 of the putative recombination events involved DQ pairs expressing the DQA*022:08 allele. (iii) DQ/DR haplotypes: in the Holstein‐Friesian and Zambian cohorts, there were 20 and 100 different DR/DQ haplotypes, respectively. Within the Holstein‐Friesian cohort, putative recombination events between the DR and DQ loci generated an additional 2 DQ/DR haplotypes (10%) because of DQ pairing with multiple DR loci (Figure 6A). In the Zambian cohort, putative DR/DQ recombination events results in the generation of an additional 29 DR/DQ haplotypes (29%) because of DQ loci forming associations with multiple DRB3 alleles; 6 additional DR/DQ haplotypes (6%) also appeared to arise as a consequence of DRB3 allele MU/GC events (Figure 6B). (iv) MHCI/DR haplotypes: In total, 50 and 457 different MHC/DR combinations were identified in the Holstein‐Friesian and Zambian cohorts (Figure 6). Of these, 27 (54%) and 299 (65.4%) appear to have been generated by recombination events leading to MHCI haplotypes being associated with multiple DRB3 loci. DRB3 allele MU/GC events were also evident in the Zambian cohort, with a total of 10 (2.2%) MHCI/DR combinations appearing to have arisen because of mutation/gene conversion of the DRB allele. We did not attempt to assess the complete MHCI dataset for recombination and mutation events within the MHCI region (because of the more ambiguous gene organisation within this region). However, there was evidence for MU/GC of MHCI genes leading to the formation of ‘variant’ MHCI haplotypes (i.e., MHCI haplotypes composed of genes from the same groups, but of which one or more differed at the allelic level); putative recombination events also contributed to the creation of novel MHCI haplotype ‘variants’ by the addition/deletion of genes (Supplementary Data 6). A total of 26 different ‘variant’ MHCI haplotype groups, comprising 63 MHCI haplotypes, were identified in the Zambian cohort, demonstrating that even this partial evaluation provided evidence of both MU/GC and recombination events contributing substantially to MHCI haplotype diversification.

Thus, within both cohorts, there was evidence indicating that MU/GC and recombination occurring throughout and between the DQ, DR and MHCI loci were contributing substantially to the diversification of bovine MHC repertoires. Whilst this was observed in both the Holstein‐Friesian and Zambian cohorts, it appeared to be generally more pronounced in the latter.

### 3.7 Breed‐associated MHC alleles and haplotypes in Zambian cattle

**FIGURE 7:**
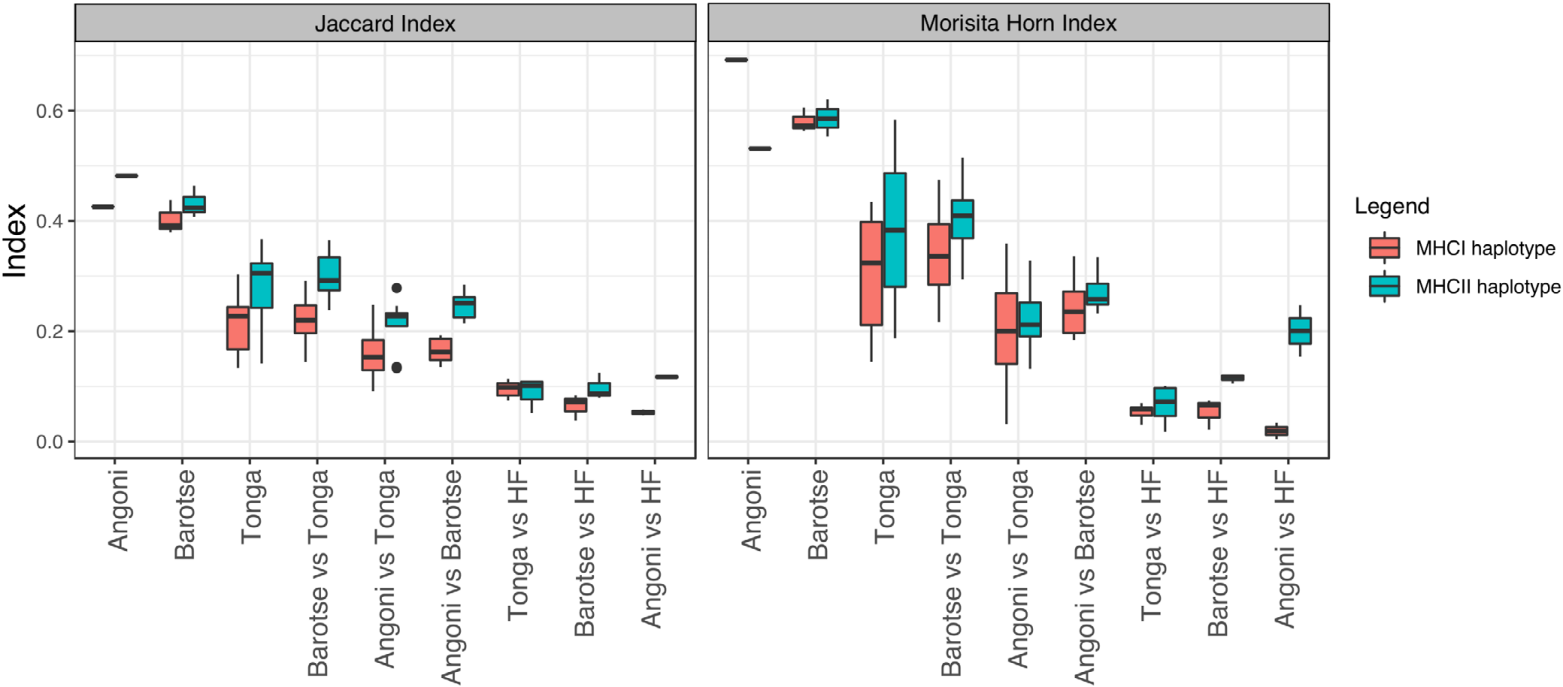
Pairwise comparison of expressed MHCI and MHCII haplotypes calculated using the Jaccard and Morisita‐Horn indices. For both indices the score ranges from 0 to 1—the higher the score the more overlap there is between the populations being compared. The Jaccard index provides a measure of the number of MHC haplotypes shared between populations, the Morisita‐Horn index compares the overlap of MHC haplotypes, weighted by frequency, between populations. Comparisons are made between different sampling sites; because of small sample sizes (<20 animals), data from the following sites was not included: for MHCI and MHCII—Angoni from Mkanile, Tonga from Shababwa, Baila from Lubanaga Banga and for MHCII only—Tonga from Settlement. The analysis was based on comparison of 212 Angoni from 2 sites, 226 Barotse from 3 sites and 308 Tonga from 6 different sites (212 Tonga from 5 farms in case of MHCII).

The Zambian cohort included cattle from the 4 indigenous breeds—Angoni, Barotse, Tonga and Baila (although for the latter very few individuals were included), which have been considered thus far as a single Zambian cohort rather than as representatives of the multiple breeds. To evaluate how similar the MHCI and MHCII repertoires present in these breeds were, we measured repertoire overlap using the Jaccard (proportion of MHC haplotypes shared between two groups) and Morisita‐Horn (proportion of MHC haplotypes, weighted by frequency, shared between two groups) indices. The Jaccard index scores for within breed comparisons (treating individual sampling sites as populations for comparison) for the Angoni and Barotse were ~0.4 for both the MHCI and MHCII (Figure 7 – with a Jaccard index value of 1 indicating a complete overlap and 0 no overlap), this was notably higher than the equivalent values obtained for the within breed comparisons for the Tonga group (~0.2 for the MHCI and ~0.3 for the MHCII), which were more similar to the scores observed for the inter‐breed comparisons seen between Tonga, Barotse and Angoni populations (ranging from 0.09–0.29 for MHCI and 0.13–0.36 for MHCII). This suggests that whilst the Angoni and Barotse have some element of breed‐specific MHC signatures, the MHC repertoires of individual Tonga populations showed as much similarity with MHC repertoires in Angoni and Barotse as with other Tonga populations. The Morisita‐Horn values were generally higher than the equivalent Jaccard values (Figure 7), indicating that the more frequently observed MHCI and MHCII haplotypes were more often shared between populations. The Morisita‐Horn index scores indicated a greater differentiation between the breeds than observed with the Jaccard indices; the values obtained for comparisons between the Angoni and Tonga/Barotse lower than those obtained for Tonga versus Barotse (Figure 7); indicating the Angoni to be more dissimilar to the other Zambian breeds, although the difference was not substantial. In general, the overlap observed between the Holstein‐Friesian and Zambian breeds was low (0.003–0.07 and 0.0–0.24 for the MHCI and MHCII, respectively, for the Morisita‐Horn index); showing a divergence between the European and Zambian breeds' MHC repertoires; the single exception was the Morisita‐Horn value for the comparison for the Angoni vs Holstein‐Friesian MHCII repertoires (0.24), which was similar to the level of overlap observed between the Angoni and other Zambian breeds.

**FIGURE 8:**
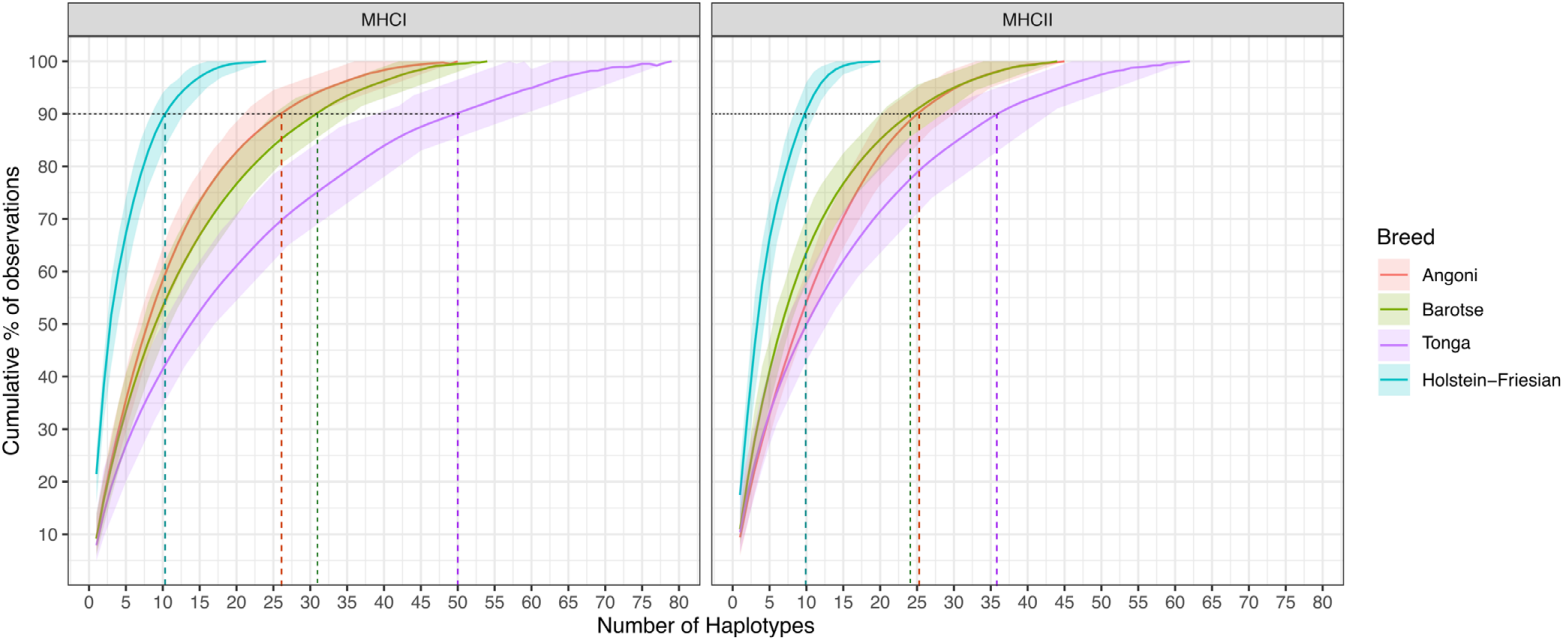
Cumulative frequency of observed MHCI and MHCII haplotypes in Angoni (red), Barotse (green), Tonga (purple) and Holstein‐Friesian (cyan) populations. To normalise for differences in the size of the cohorts samples for the different breeds a sub‐sample of 100 random animals from each breed was analysed with bootstrapping (*N* = 100). The mean cumulative observed frequency derived from the bootstrapping for each breed is shown as a line, with a distribution of the distribution of the values represented as a ribbon. The *D*_90_ values for each breed are highlighted with dashed lines.

We also evaluated the diversity of the MHC repertoires for the individual breeds, using the the *D*_90_ values for the MHCI and MHCII repertoires (i.e., the number of MHCI/MHCII haplotypes that accounted for 90% of the MHCI/MHCII haplotype observations—Figure 8). For MHCI, the values were highest for Tonga (*D*_90_ = 50) and slightly lower for the Barotse and Angoni (*D*_90_ = 32 and 27 respectively), whilst the diversity of the MHCII was generally lower, with the *D*_90_ for the Tonga being 36 and for the Angoni and Barotse populations being 26 and 24, respectively. For comparison, the *D*_90_ values for the MHCI and MCHII in the single herd Holstein‐Friesian population were 11 and 10. Assessment of MHCI and MHCII diversity in the different breeds as measured by a number of indices provided comparable results showing similar levels of diversity in the Zambian breeds, but with Tonga slightly higher than the others (Supplementary Data 7).

## 4 DISCUSSION

In this study, we describe the development and application of a high‐throughput sequencing approach for analysis of bovine DQA and DQB alleles. By combining this with equivalent protocols for bovine MHCI and DRB alleles,^14^, ^15^ it is now possible to conduct high resolution and high throughput analysis of the classical MHCI and MHCII genotypes of cattle and rapidly expand our knowledge of the MHC repertoires in populations of cattle for which there is currently limited data available. Applying this approach, we provide a comprehensive analysis of the MHCI/MHCII haplotypes present in a large cohort of animals from indigenous Zambian cattle breeds.

The system developed for the analysis of DQA and DQB had the same fundamental basis as that we had previously developed for the DRB,^15^ with the design of the PCR and the bioinformatics pipelines following the same rationale. The only elements that required bespoke validation were the thresholds used for read frequency cut‐off, which were empirically determined, by analysis of data from a Holstein‐Friesian cohort, to be 7% and 2% for DQA and DQB, respectively. Overall, the approach was effective with the combined MHCI/MHCII repertoires described for a total of 326/327 (99.7%) Holstein‐Friesian and 627/800 (78.4%) Zambian cattle; however, there were a number of technical limitations to the system. Firstly, for some Zambian samples there was insufficient read depth to reliably confirm the expressed MHC alleles; for 16 animals this resulted in loss of both the MHCI and MHCII genotypes, and for an additional 109 animal the loss of the MHCII genotypes. The greater frequency of MHCII genotype loss was because of the need to achieve sufficient read depth for 3 separate PCRs amplicons (DRB, DQA and DQB) to define MHCII genotypes, whereas for the MHCI there was a degree of redundancy between the 2 PCR reactions used to generate amplicons. For the Zambian cohort, 960 separate amplicons (192 animals × 5 PCR reactions) were ‘pooled’ and sequenced in each MiSeq run, and no normalisation of libraries was conducted as this would have incurred substantial financial and labour costs (the number of amplicons included in the sequencing runs used for the Holstein‐Friesian cohort were slightly lower). It would therefore seem that this approach to library preparation is at, or just beyond, the capacity of what can be accommodated. For future studies, a reduction in the number of samples included in each MiSeq run, and/or normalisation of DNA input from each individual PCR reaction, may reduce the number of individuals for which insufficient read numbers are obtained. However, these options would either reduce the number of individuals that can be included in each run and/or significantly increase labour and financial costs. Therefore, although there is some loss of data when conducted at scale, the approach adopted here affords an acceptable compromise between input and output in studies, such as this, where the generation of data from all individuals sampled is not essential to achieving the study's aims. For some of the individuals for which the read depth was not sufficient to pass the automated threshold in the pipeline, the MHC haplotypes could be inferred retrospectively (data not shown). The second technical limitation of the study was the ‘allele dropout’ observed for DQA. We employed a similar approach to the design of primers for all 3 MHCII genes, with primers located in the conserved exons 1 and 3 to enable amplification and sequencing of the entire polymorphic exon 2. Whilst this approach appears to have worked well for DRB and DQB, there were 11 DQB alleles where (an assumed) partner DQA allele was not sequenced. Whilst it is possible that some of these represent situations where the DQA is genuinely absent or non‐expressed, the most likely explanation is amplification failure because of mismatches in the primer annealing sites. A contributing factor to this problem was that most available bovine DQA allele sequence data has been generated from amplicons generated using primers located in the introns flanking exon 2, and so there is minimal exon 1 and 3 sequence data in the IPD database on which to base primer design. As a consequence, the capacity to address the issue through re‐design of primers (either by changing annealing sites or introducing further degeneracy) is limited, whilst the option of using multiple DQA primer sets to reduce ‘allele dropout’ (as has been done for MHCI) may exacerbate the issue regarding the number of amplicons that can be accommodated in each MiSeq run. We have been able to successfully infer the MHCII haplotypes in most animals where DQA ‘dropout’ was observed and so consider that the best approach at the moment is to retain the current primers but complement the MiSeq data with targeted extended sequencing of DQA alleles to generate the requisite exon 1 and 3 data to complete the MHCII haplotype characterisation and enable primer re‐design at a future time.

Despite these limitations the approach was successful in allowing us to achieve the primary objective of the study, which was to characterise the combined MHCI‐MHCII repertoires of indigenous cattle populations in Zambia. Our sample‐set included animals from the 3 major breeds—Angoni (zebu – primarily *Bos indicus* but with evidence of *Bos taurus* introgression), Barotse and Tonga (both Sanga breeds—i.e., African *Bos taurus* × zebu) as well as a small number of Baila cattle (considered to be a cross between Barotse and Tonga), for which MHC sequence data was previously unavailable. A total of 260 MHCI, 56 DRB3 38 DQA and 55 DQB alleles were identified in the Zambian cohort—of which 63 (24.2%), 2 (3.6%), 7 (18.4%) and 9 (16.4%) were novel respectively. The low proportion of novel DRB3 alleles is likely a consequence of the widespread characterisation of bovine DRB3 diversity using SBT methodologies,^25^, ^26^, ^27^, ^28^, ^29^ whilst the MHCI, DQA and DQB repertoires have been less intensively studied because of the absence, prior to NGS, of high‐throughput sequencing approaches. Notably, only 1 of the DQA and 1 of the DQB alleles represented members of novel allelic groups, whilst members of 16 putative novel MHCI allelic groups were identified. This suggests that, as in humans, there is more diversity at the allele‐group level in the MHCI than the MHCII loci; this is supported by the number of total allelic groups identified in the Zambian cohort—147 MHCI putative allelic groups were identified, compared with 40 DRB, 21 DQA and 34 DQB allelic groups.

Within the Zambian cohort a total of 158 MHCI and 100 MHCII haplotypes were identified, of which 80 and 90 were novel (including putative unconfirmed haplotypes), respectively. The high number of novel MHCII haplotypes is largely attributable to the fact that there have been limited studies conducting parallel sequencing of DRB and DQA/DQB^12^, ^19^, ^20^, ^21^, ^22^ and, as far as the authors as aware, this is the first time such work has been conducted on a large‐scale outwith the Holstein‐Friesian breed. For MHCI the high number of novel haplotypes is partly because of haplotypes including novel alleles; however of the 56 confirmed novel MHCI haplotypes, a significant proportion (30.5%, *n* = 17) were composed only of previously known alleles (with the caveat of only having partial cDNA sequence data; it is possible that there are variations outwith this area)—indicating that formation of new permutations of MHC alleles through recombination was having a substantial role in diversifying the repertoire of MHCI haplotypes. The structure of our data did not permit a formal analysis of the genetic evolution of the MHC via recombination, mutation or gene conversion events, which requires longitudinal data following familial groups or colonies or other formats enabling inter‐generational tracking.^30^, ^31^, ^32^, ^33^ However, using proxies for recombination and mutation/gene conversion it appeared that within the Zambian (and, to a lesser extent, the Holstein‐Friesian) cohort there was evidence for putative examples of each occurring throughout and between the MHCI and MHCII regions. Although, in the absence of data from a pedigree‐defined population, the details of recombination and mutation/gene conversion in diversifying the MHC of the Zambian cohort remains speculative, the conclusions inferred from the data are not controversial—the roles of recombination and mutation/gene conversion in MHC evolution have been well established in multiple species.^32^, ^33^, ^34^, ^35^ A couple of specific features of the diversity of the MHC observed in the Zambian cohort were note‐worthy. The first of these was the discrepancy in the number of variants observed between different allele groups; for example 90 MHCI groups had only a single member, whereas for others (e.g., BoLA‐3*004) upto 10 different alleles were observed; suggesting that some alleles may be more susceptible to mutation/gene conversion events. In many cases these allelic variants were associated with the generation of ‘variant’ haplotypes (Supplementary Data 6 – haplotypes that share the same MHCI allele composition except for one (or multiple) genes with different members of the same allele group expressed); interestingly the ‘variants’ were frequently the dominant or co‐dominantly alleles expressed—suggesting a potential linkage between diversification and quantity of peptide presentation (assuming higher expressed MHC alleles present a greater quantity of peptides). The second observation was that certain BoLA‐6 and BoLA‐3 alleles appeared to be ‘appended’ to multiple MHCI haplotypes—creating another form of ‘variant’ haplotype (Supplementary Data 6). These two MHCI loci have recently been confirmed to be duplicated in some MHCI haplotypes.^5^ The recurrence of BoLA‐6 and BoLA‐3 loci being ‘appended’ to MHCI haplotypes and their duplication in some MHCI haplotypes suggests that these loci (or a subset of alleles of these loci) may be more liable to uneven recombination cross‐over events during meiosis—confirmation of this and investigation into its potential functional consequences would be of interest. The third observation related to the BoLA‐DQA*022:08 allele, which was observed to form pairs with a high number of DQB partners and was correspondingly over‐represented in the Zambian population (Figure 3C). The promiscuity of BoLA‐DQA*022:08 was also observed in the Holstein‐Friesian cohort, suggesting that some form of allele‐specific recombination ‘hotspot’ was associated with this allele. A fourth observation was that recombination in the DQ region had led to sufficient numbers of different permutations of the same DRB, DQA and DQB alleles, that for some animals it was possible to resolve the MHCII haplotypes in multiple ways—consequently the phasing of the MHCII haplotypes was not possible in these animals (*n* = 48). In summary our analysis provides some preliminary inferences of how the bovine MHC repertoire is diversified by recombination and mutation/gene conversion events and highlighted some interesting examples; however from an immunological perspective perhaps the most notable feature was the level of MHC diversity observed in the Zambian cohort.

No equivalent combined bovine MHCI‐MHCII datasets, against which the diversity observed in the Zambian cohort can be directly compared, are available from previous studies (as far as the authors are aware). In this study we conducted parallel analyses of the Holstein‐Friesian cohort, however comparison with the Zambian cohort is confounded by the fact that the former was composed of animals from a single breed and from a single farm. A more equitable comparison is with the cohort of Brazilian animals published recently^15^ which was composed of 555 individuals from 4 breeds (Gyr, Guzerat, Nelore and Girlando) from 7 different farms, so of a more similar structure and complexity to the Zambian cohort; however only the MHCI and DRB was sequenced. A total of 165 different combined MHCI‐DR haplotypes were identified in the Brazilian cohort (29.7 haplotypes/100 animals)—the equivalent figure for the Zambian cohort was 457 MHCI‐DR haplotypes (72.6 haplotypes/100 animals) and the difference in complexity can be visualised by comparing Figure 6B (left side of figure) with Supplementary Data 9 in Vasoya et al. (2021).^15^ A number of factors may contribute to the disparity between the Brazilian and Zambian cohorts, but a major factor is likely to be the relatively small founder populations of the *Bos indicus* breeds in Brazil and continuing genetic selection as a consequence of selective breeding.^36^ Similarly, multiple analyses have shown global populations of the Holstein‐Friesian breed to have small effective population size because of intensive selection pressure and a commensurate loss of genetic variability^37^, ^38^, ^39^; in this study the number of MHCI‐DR haplotypes in the Holstein‐Friesian cohort was 14.8/100 animals, although this needs to be considered with caveats detailed above. Together this data suggests that the MHC repertoire diversity of Holstein‐Friesian and similar breeds which have been subject to hierarchical breeding strategies and intensive selection (e.g., widespread use of artificial insemination from a limited number of high‐performing bulls) are not representative of the diversity present in other cattle populations. In Zambia there is limited breeding selection—most breeding (98%) occurs through natural, indiscriminate mating by animals during communcl grazing^16^ and artificial insemination use is negligible (0.2%). As the bull to cow ratio is 1:11, this traditional, non‐selective breeding management structure promotes the maintenance of high levels of genetic diversity, and as our data indicates, a MHC repertoire that is much more diverse than that observed in more commercial breeds.

The indigenous cattle breeds of Zambia are named according to region and the tribes with which they are traditionally associated and although there are breed‐associated phenotypes and genetic characterisation, the concept of breed is less rigidly applicable than for European *Bos taurus* breeds. Of the 3 major breeds included in the study, only the Angoni has a registered herd book and remains distinct from the Tonga and Barotse breeds because of its more pronounced *Bos indicus* phenotype as well as its geographical separation. In contrast, the Tonga and Barotse breeds are less distinct, both being sanga breeds and in closer geographical proximity. There is some suggestion that in border areas between the Barotse (Lozi) and Tonga people, there is mixture between the Tonga and Barotse cattle (shorturl.at/buvzK). In addition, following repeated raids in the late 19th century, Tonga communities restocked depleted cattle populations mainly with Barotse animals (shorturl.at/bDQS1), further mixing the genetics of these two breeds. These population interactions were reflected in the degree of similarity exhibited by the MHC repertoires of the 3 breeds; intra‐breed comparisons suggested a higher level of in‐breed homogeneity in the MHC repertoires of both the Angoni and Barotse populations (Morisita‐Horn index scores of >0.5), whilst for the Tonga, the in‐breed comparisons suggested more heterogenous repertoires between sampling sites (Morisita‐Horn scores of 0.14–0.58)—perhaps indicative of this breed's diversity being increased because of introduction of Barotse genetics during re‐stocking of populations. The close relationship of the Tonga and Barotse breeds was also reflected in the inter‐breed comparisons, where the similarity between the Barotse and Tonga MHC repertoires (Morisita‐Horn scores of 0.2–0.5) were similar to that observed for the intra‐breed comparisons for the Tonga—implying that the MHC repertoire present in the Barotse may largely represent a subset of that observed in the Tonga. Similarly, the segregation of the Angoni from the Tonga/Barotse was reflected in the low MHC overlap in these inter‐breed comparisons (Morisita‐Horn scores of 0.03–0.36).

Zambian populations showed very little similarity to the MHC repertoire in Holstein‐Friesian, the breed in which MHC has been most intensively studied; most Morisita‐Horn scores comparing Holstein‐Friesian to Zambian populations were ~0.1. This reaffirms that the MHC repertoire identified in the Holstein‐Friesian is not representative of other cattle breeds and that a more comprehensive characterisation of the global cattle MHC diversity requires the analysis of a greater range of different cattle populations. Indigenous African cattle breeds are ideal populations in which to initiate these studies because of a combination of (i) the genetic complexity of many indigenous breeds, where there are varying levels of admixture of European *Bos taurus*, African *Bos taurus* and *Bos indicus* lineages,^40^, ^41^, ^42^ (ii) less selective pressure and hierarchical breeding structures have been employed—so enabling the maintenance of greater genetic diversity and (iii) these populations are exposed to a wide range of pathogens—which have been implicated as major drivers of MHC selection. In Zambia important cattle diseases include those caused by tick‐borne pathogens such as *Theileria, Babesia, Anaplasma* and *Ehrlichia* as well as Foot‐and‐Mouth Disease Virus, Lumpy Skin Disease Virus, Mycoplasma, *Trypanosoma* and Clostridium; although there is anecdotal evidence that indigenous breeds are more tolerant of many of these pathogens, there is a paucity of scientific data to confirm this. Now is the apposite time in which to undertake these studies as accelerating use of selective cross‐breeding with ‘exotic’ European *Bos taurus* animals threatens a loss of the genetic diversity that currently exists.

In Zambia, as in many low‐middle countries, cattle represent not just a means to provide high quality protein and contribute to farmer's and national food security—they are also a means of financial security, provide important draught power for agricultural and transportation, assist in soil regeneration as well as serving other societal purposes.^16^ As such, diseases which can devastate cattle have the potential to severely negatively affect small‐holder farmers. For many of these diseases there remains a need for the development of novel control strategies, such as the generation of new vaccines. For diseases such as East coast fever, which was reported as the priority disease by ~25% of small‐holder cattle farmers in Zambia,^16^ these vaccines will need to elicit T‐cell responses. Consequently, it is imperative that in parallel to characterising the MHC repertoires in indigenous cattle populations, work that facilitates the transition of this knowledge into information that can be used to ensure future vaccines incorporate antigens relevant to these populations (e.g., immunopeptidomic analysis), is conducted.

In conclusion, we present a NGS sequencing system that enables, for the first time, comprehensive characterisation of the combined classical MHCI and MHCII repertoires of cattle that can be used to analyse data from large cohorts of animals. The data from the Zambian cohort has continued to expand the known repertoire of individual MHCI and MHCII alleles, generated information on a large number of novel MHCI and MHCII and combined MHCI/MHCII haplotypes and provided some preliminary observations on the role of mutation and recombination in diversifying the bovine MHC repertoire. We hope the approach will find application in continuing to study the MHC diversity of cattle populations from across the globe and be used to support translational outputs such as the engineering of novel T‐cell inducing vaccines that are applicable to a broad proportion of the global cattle population, including those animals that sustain the livelihoods of some of the most vulnerable farming communities in the world.

## AUTHOR CONTRIBUTIONS

Isaac Silwamba, Martin Simuunza, Edgar Simulundu, collected and performed preliminary preparation of samples, Isaac Silwamba, Thomas Tzelos, Christina Vrettou, conducted the laboratory work, Deepali Vasoya and Mick Watson performed the bioinformatics analysis, Timothy Connelley, John Bwalya Muma and King S. Nalubamba designed and conceived the study and Timothy Connelley, Isaac Silwamba and Deepali Vasoya wrote the manuscript. All authors have read and approved the final manuscript.

## CONFLICT OF INTEREST STATEMENT

The authors declare no conflicts of interest.

## Supporting information


**Supplementary Data 1** Summary of MHCII sequencing data for a cohort of 327 Holstein‐Friesian cattle. For each sample the sample identification (column A) and the breed and lineage of the animal from which the sample was taken (column B and C respectively; HF = Holstein Friesian, BT = european *Bos taurus*) are shown. Comments on the MHCII haplotyping are provided in column D; 3 haplotypes = 3 MHCII haplotypes identified in the sample; Homozygous = a single MHCII haplotype identified in the sample; No DQA = No DQA sequences obtained for sample; Ambiguous = alleles could not unambiguously be assigned to haplotypes (on red background). The number of reads for each sample that passed filtering and were used for haplotyping for DRB3, DQA and DQB are shown in columns E–G. The number of DRB3, DQA and DQB alleles identified in each sample are shown in columns H–J and the defined haplotypes are shown in columns K‐M. Details of the sequences in haplotypes 1, 2 and 3 are shown in columns O‐AC, AE‐AS and AU‐BI respectively. Details of DRB3, DQA and DQB sequences not assigned to haplotypes are provided in columns BK‐BP, BR‐BW and BY‐CG respectively.

**Supplementary Data 2** Fasta files for all novel MHCI and MHCII sequences identified in this study.

**Supplementary Data 3** Summary of MHCI and MHCII sequencing data for Zambian cattle. For both the MHCI and MHCII data the sample identification (column A) and the breed and lineage of the animal from which the sample was taken (column B and C respectively; BI = *Bos indicus*) are shown and the district and site of sampling are detailed in columns D and E. *MHCI*: Comments on the MHCI haplotyping are provided in column F; 3/4 haplotypes = 3/4 MHCI haplotypes identified in the sample; Homozygous = a single MHCI haplotype identified in the sample; No for1/rev2 and No for3/rev1 = no amplicon reads for primer set; insufficient data = samples removed because of insufficient read data to support haplotyping (on red background); contamination = samples removed because of apparent contamination (on red background). The number of reads for each sample that passed filtering and were used for haplotyping from the For1/rev2 and For3/rev1 primers are recorded in columns G and H and the defined haplotypes are recorded in columns I‐L. Details for the alleles in haplotype 1, 2, 3 and 4 are detailed in columns N‐AL, AN‐BL, BN‐CG and CI‐DB. Alleles that could not be assigned to haplotypes, or were in samples excluded from subsequent analysis because of insufficient data or contamination are shown in columns DD‐EL, with unassigned alleles with >1% of read frequency shown on a blue background. *MHCII*: Comments on the MHCII haplotyping are provided in column F; 3/4 haplotypes = 3/4 MHCII haplotypes identified in the sample; Homozygous = a single MHCII haplotype identified in the sample; No DQA = no amplicon for DQA primer set; insufficient data = samples removed because of insufficient read data to support haplotyping (on red background); ambiguous = alleles could not unambiguously be assigned to haplotypes (on red background). The number of reads for each sample that passed filtering and were used for haplotyping for DRB3, DQA and DQB are shown in columns G‐I. The number of DRB3, DQA and DQB alleles identified in each sample are shown in columns J‐L and the defined haplotypes are shown in columns M‐P. Details of the sequences in haplotypes 1, 2, 3 and 4 are shown in columns R‐AF, AH‐AV, AX‐BL and BN‐CB respectively. Details of DRB3, DQA and DQB sequences not assigned to haplotypes are provided in columns CD‐CU, CW‐DN, DP‐EV respectively.

**Supplementary Data 4** Scatterplot analysis of the read frequency observed with the For1/Rev2 and For3/Rev1 MHCI PCR reactions for each haplotype identified in the Zambian cohort. Scatterplots are used to illustrate the correspondence between the two independent PCRs for the normalised read frequency of the different alleles in the haplotypes. This varies between haplotypes dependent on the presence of PCR bias in the amplification of specific alleles. In previous publications for haplotypes where there was high correlation (*R* ^2^ > 0.9) between the read frequencies observed with the 2 independent PCRs, and a slope of 0.8–1.1 for the line of best fit we have assumed that the read frequency represents relative mRNA transcript expression levels. Based on this it can be observed that the different MHCI haplotypes conform to different patterns with regard to the number of alleles expressed and the expression levels of different constituent alleles.

**Supplementary Data 5** Genomic organisation of the bovine MHC locus. The bovine MHC locus is located on chromosome 23 at position 25.58–28.72 Mb in the current Ensembl annotation of genome ARS‐UCD1.2. The location of the DQ, DR and BoLA‐I genes are shown (with the Ensembl identifier in brackets). The distances between the gene loci are calculated based on the annotation; paired DQA/DQB loci are in close proximity (10.7 and 14.1 Kb), the distance between the DQ loci is 42.9Kb, the distance between the DRB3 and proximal DQ loci is 28.4Kb and the approximate distance between the DRB3 and MHCI loci is ~1.9 MB.

**Supplementary Data 6** ‘Variant’ MHCI haplotypes identified in the Zambian cohort. ‘Variant’ MHCI haplotypes express BoLA‐I alleles from the same allelic groups but with one or more differing at the allelic level. A total of 26 different ‘variant’ MHCI groups were identified in the Zambian cohort, which together included a total of 63 MHCI haplotypes (groups included between 2 to 5 different ‘variant’ haplotypes). For each haplotype the haplotype group (column A), the haplotype name (column B), the number and percentage of observations in the Zambian cohort (columns C and D respectively) and the number of alleles in the haplotype (column E) are shown. The alleles expressed in each haplotype and their normalised average read frequency in the For1/Rev2 and For3/Rev1 primer sets are shown (columns F–Q). The alleles that vary between haplotypes in the same group are highlighted in yellow. In some instances, ‘variant’ haplotypes also express additional gene(s) (shown in red script); notably in all examples were the additional gene can be attributed to a specific BoLA‐I locus it is either a BoLA‐3 or a BoLA‐6 allele

**Supplementary Data 7** MHCI and MHCII diversity analysis in Tonga, Barotse, Angoni and Holstein‐Friesian (HF), Shannon entropy, Simpson's diversity index and a profile of Hill numbers using a series of alpha parameter values were used to to analyse a sub‐sample of 100 animals randomly selected for each breed. Shannon's entropy measures the richness (diversity) and evenness (equivalence of frequency) of MHC haplotypes in a population. Decrease in diversity and the evenness in the frequency of MHC haplotypes leads to a reduction in the index value. Simpson's index is a measure that assesses diversity by both the number of MHC haplotypes present in a population as well as the relative frequency of each haplotype. The value ranges between 0 and 1, with higher values reflecting greater diversity. Hill values describe the complexity of a population with the relative effect of the frequency of different haplotypes modified depending on the alpha value (increasing alpha values cause the frequency of MHC haplotypes to have a greater impact; when α = 0 the frequency of haplotypes is not considered so the Hill number is the same as the number of MHC haplotypes in the population). In the graph the value of the Hill number is shown (vertical axis) for a range of α values (horizontal axis). For each breed the mean of the values derived from 100 bootstrap iterations is shown as a solid line, with the range of scores indicated by the ribbon as described in the legend.

## Data Availability

The data that support the findings of this study are openly available in European Nucleotide Archive at https://www.ebi.ac.uk/ena/browser/home, reference number PRJEB44287, PRJEB55564 .
