# Supplementary figures and images for: High throughput analysis of MHC class I and class II diversity of Zambian indigenous cattle populations
Source: HLA. 2023 Jan 29;101(5):458–83. doi: 10.1111/tan.14976 (PMC10952738; doi:10.1111/tan.14976)

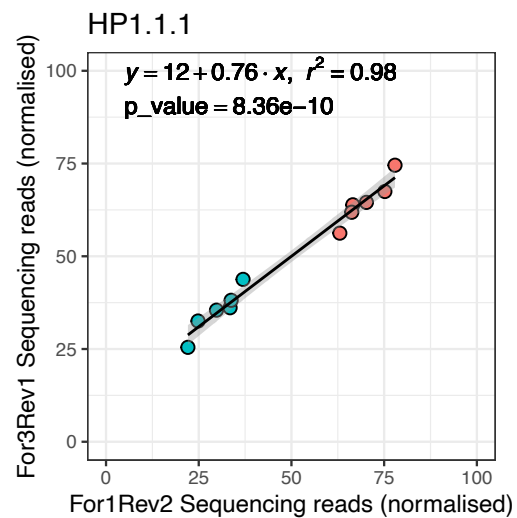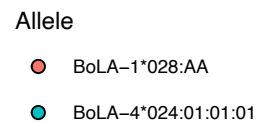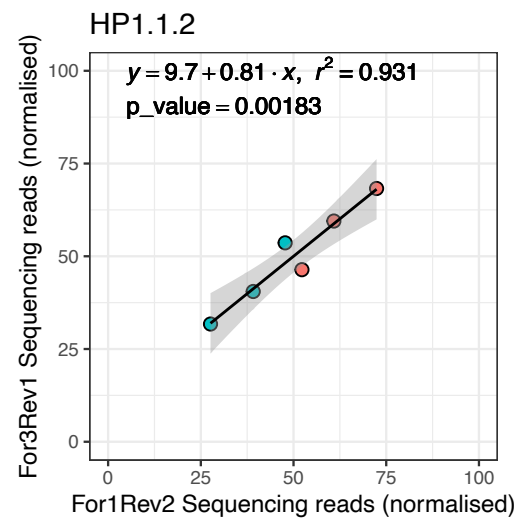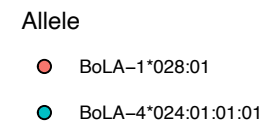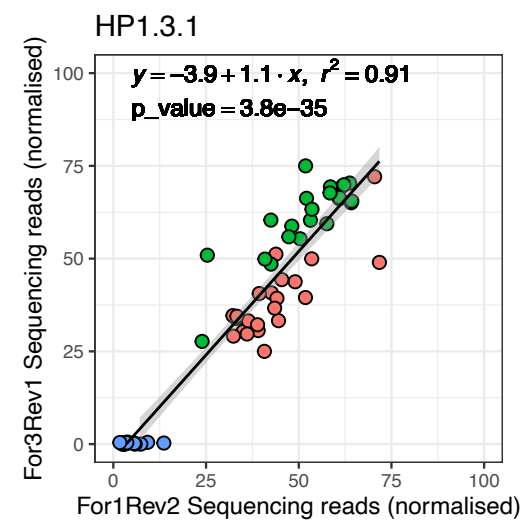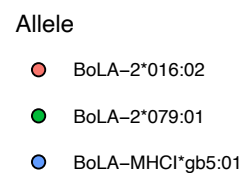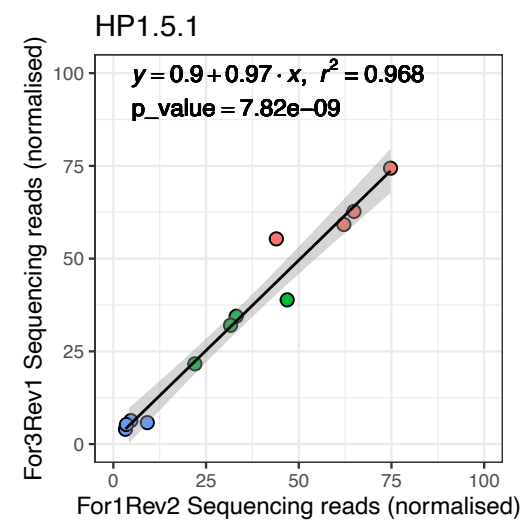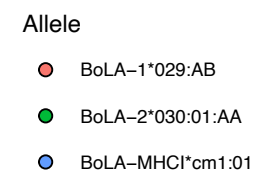

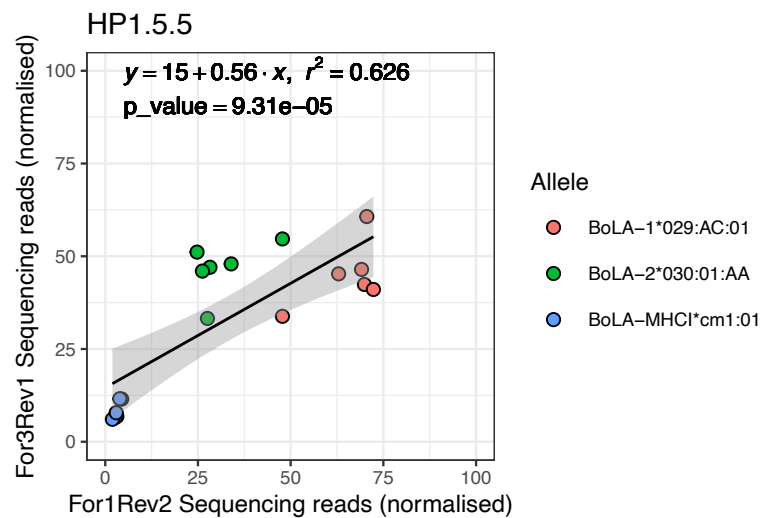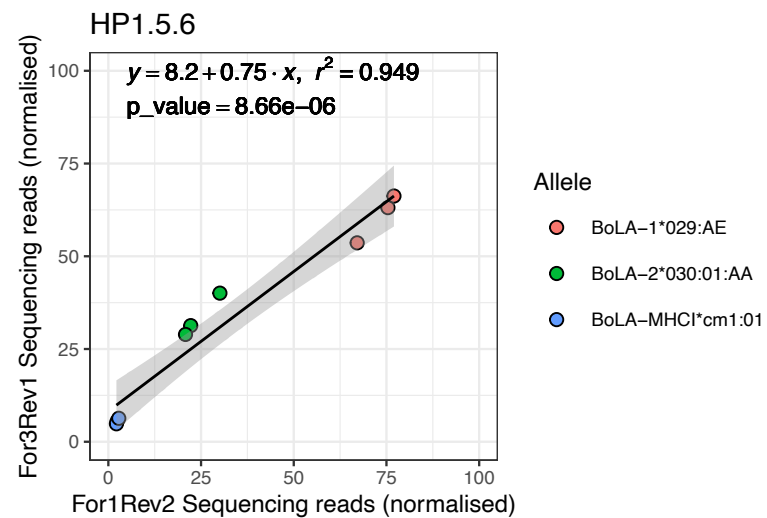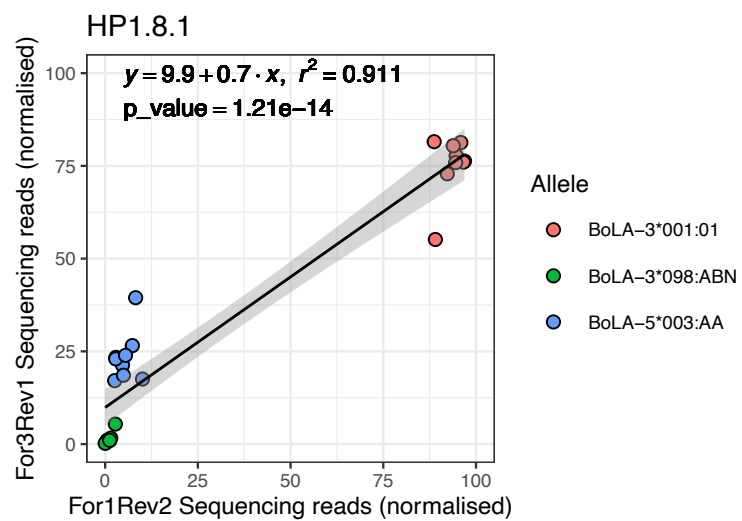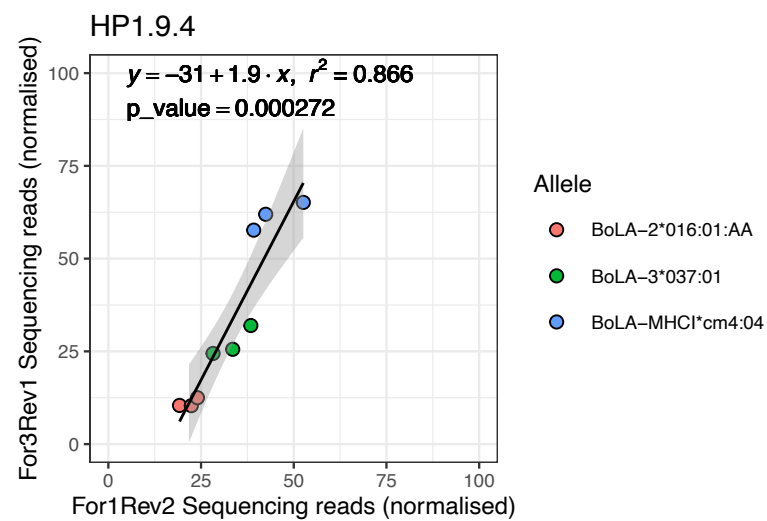

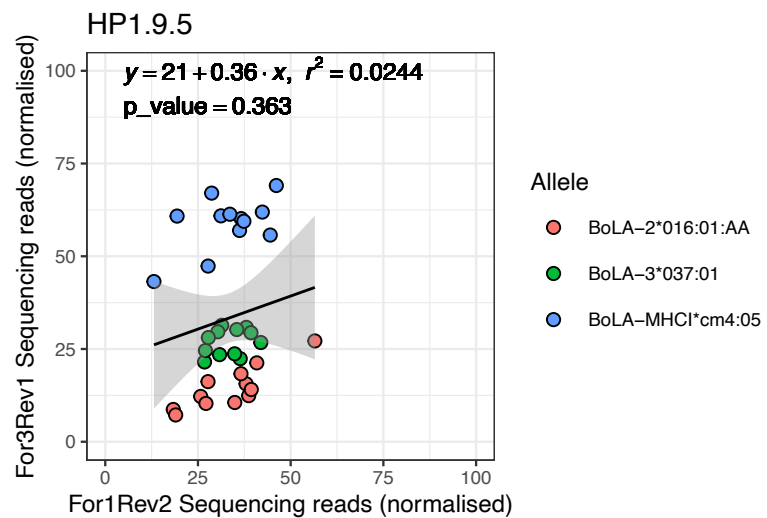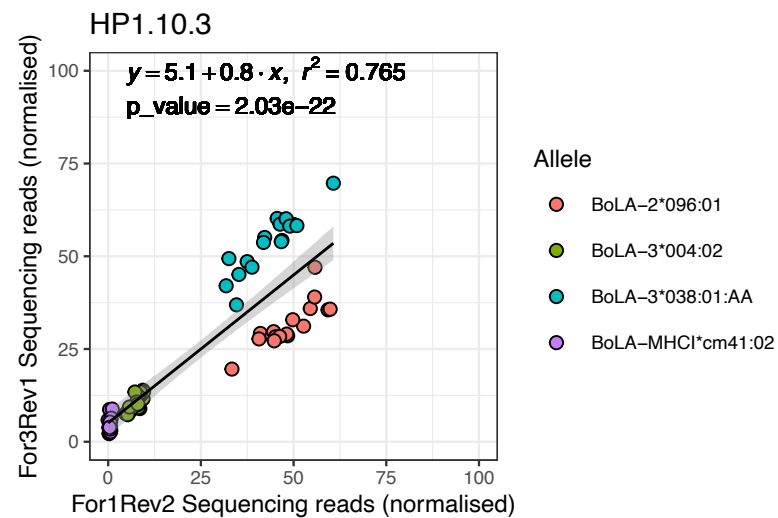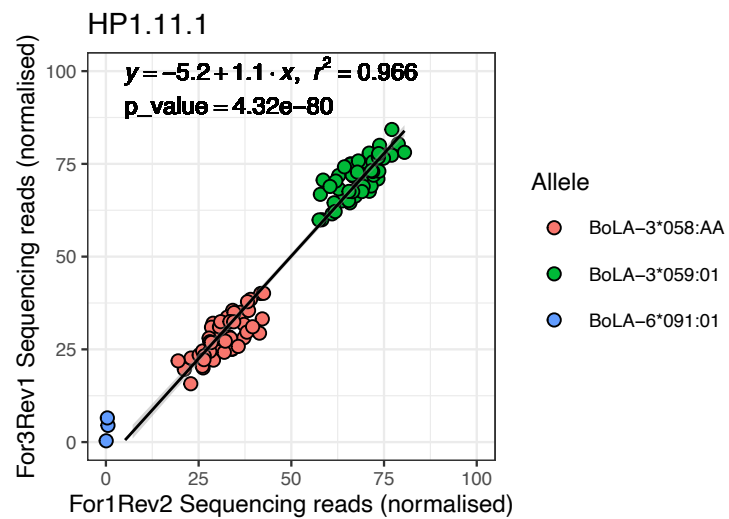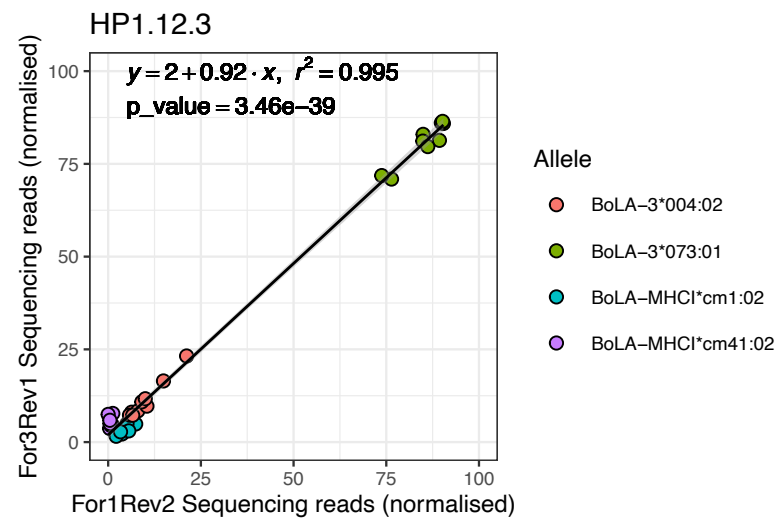

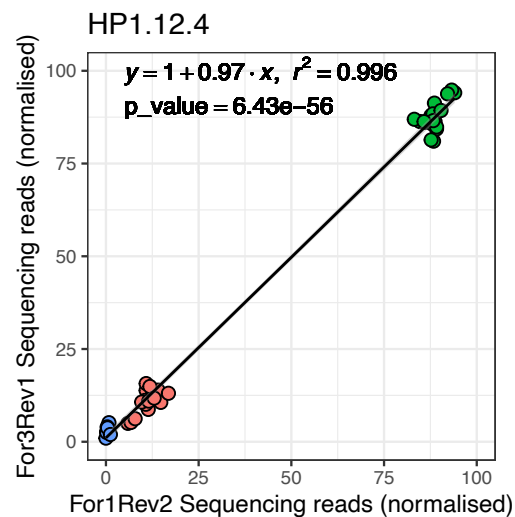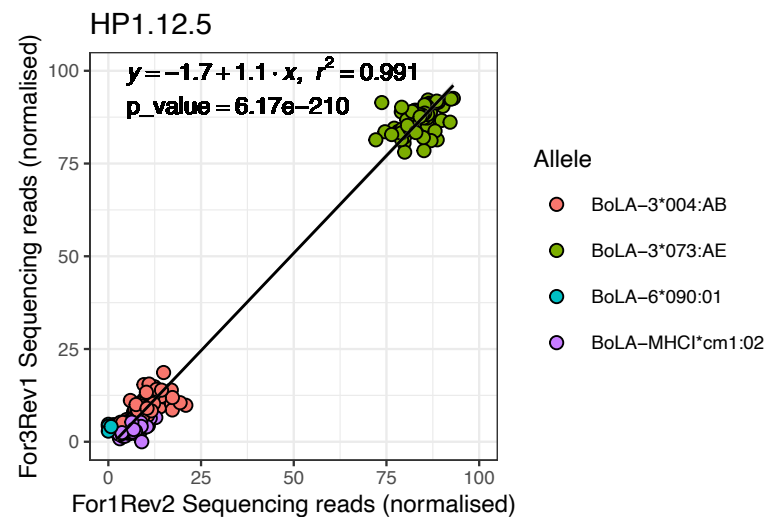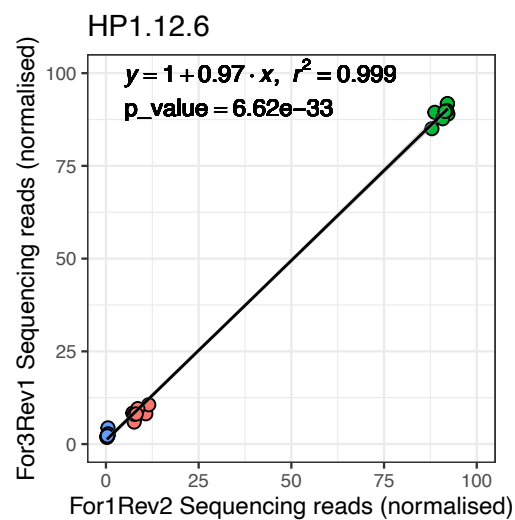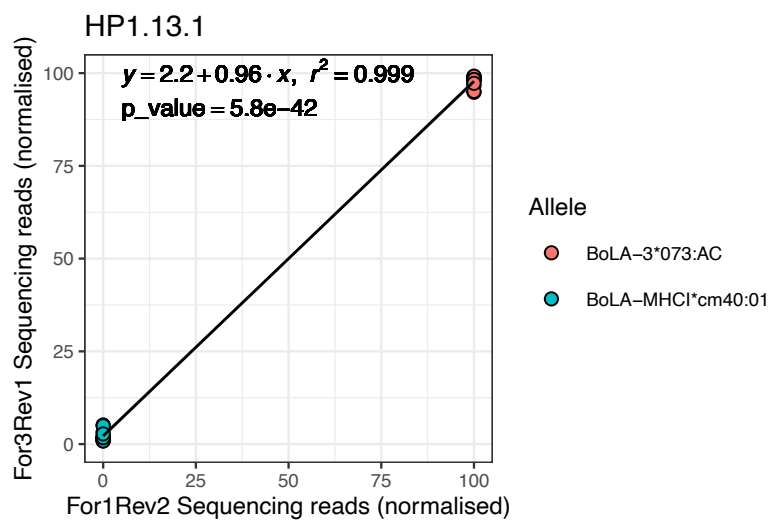

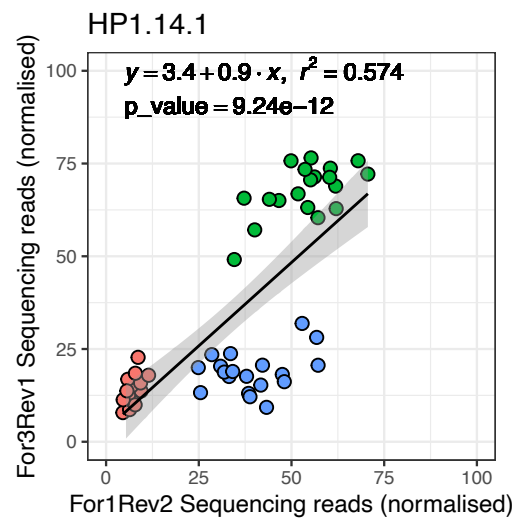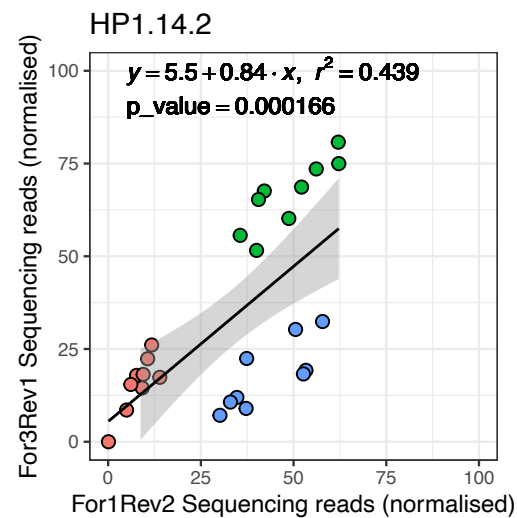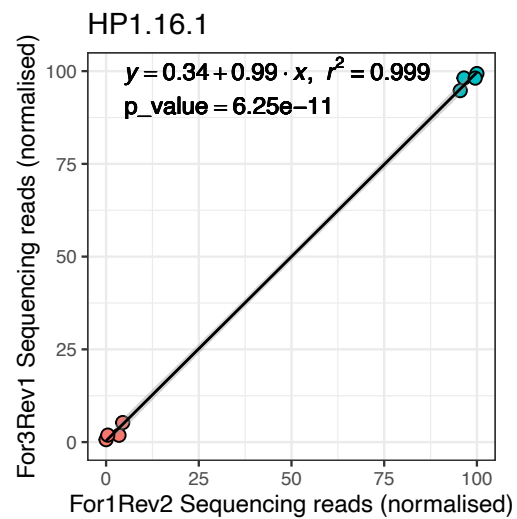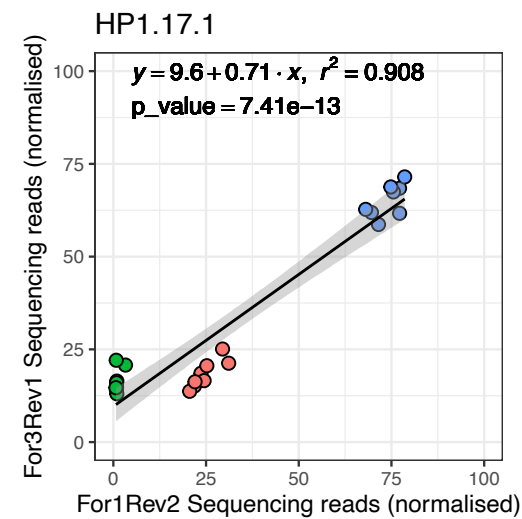

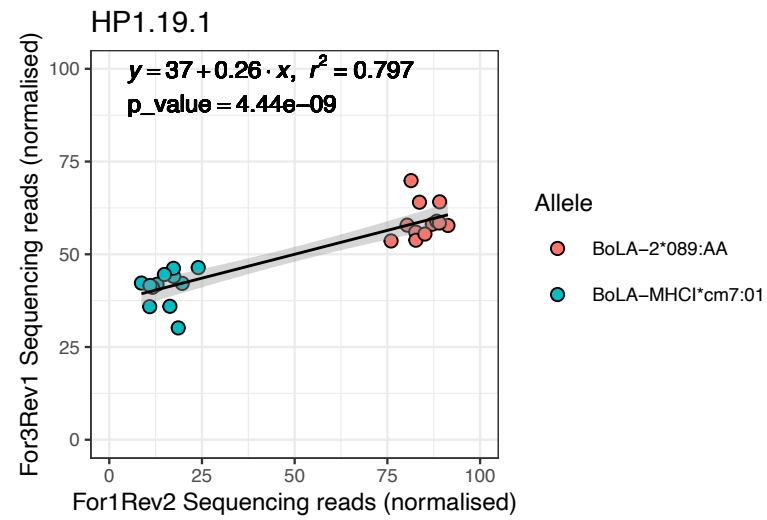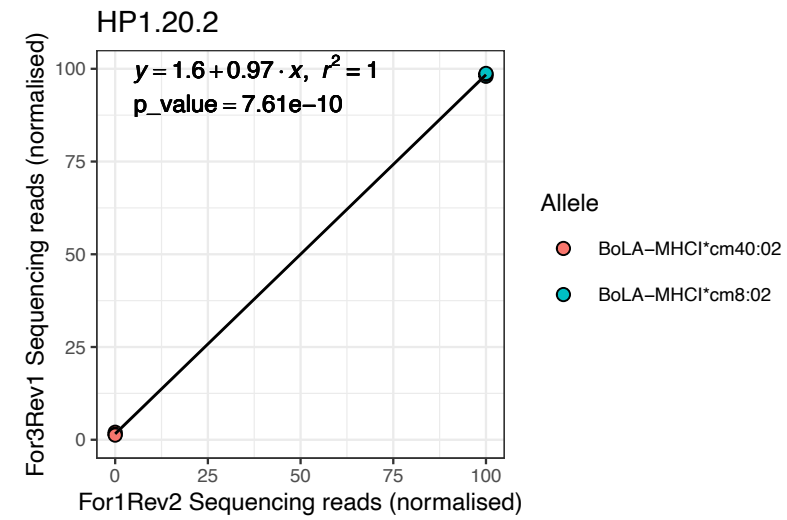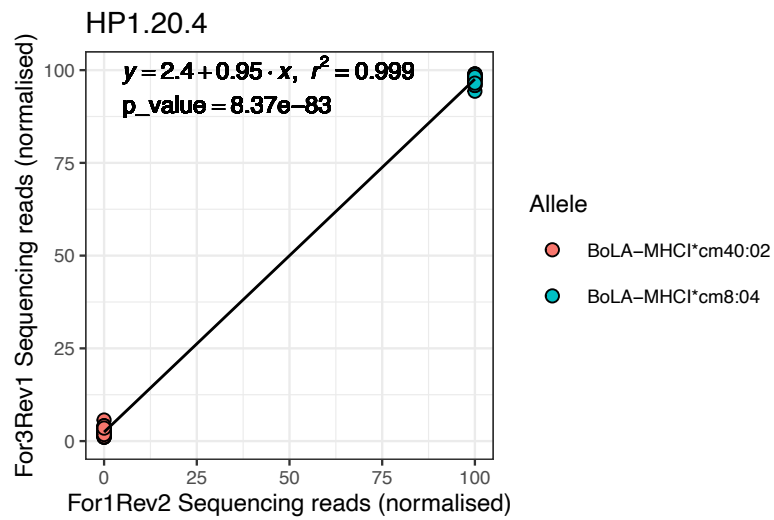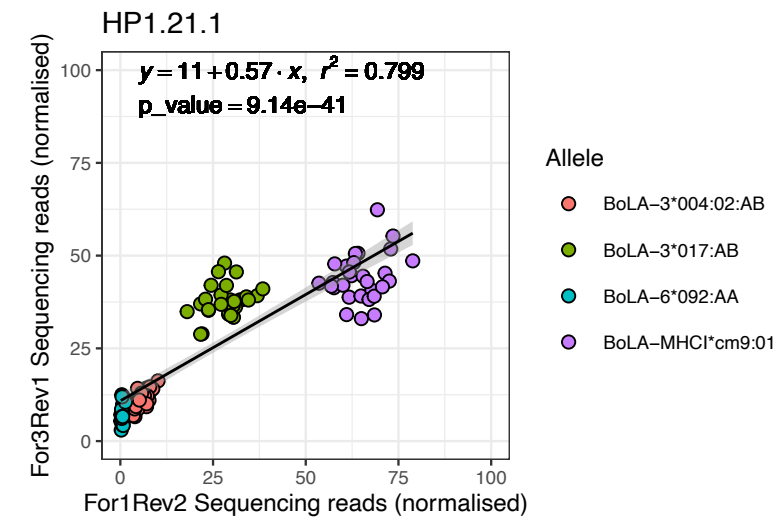

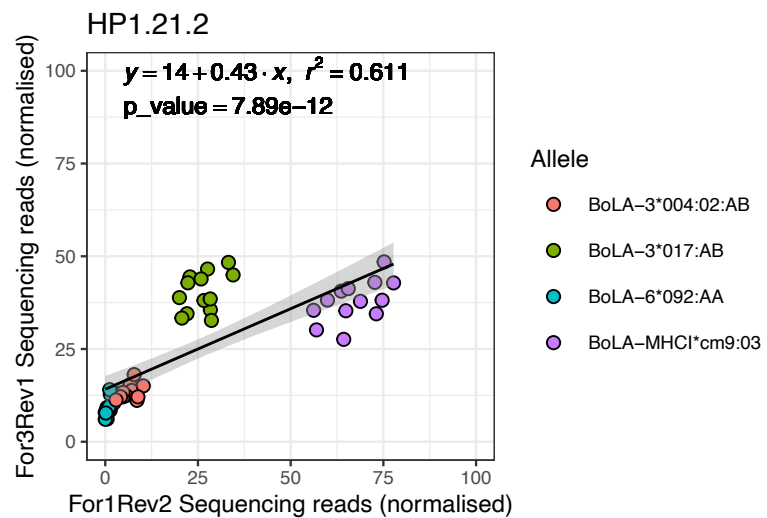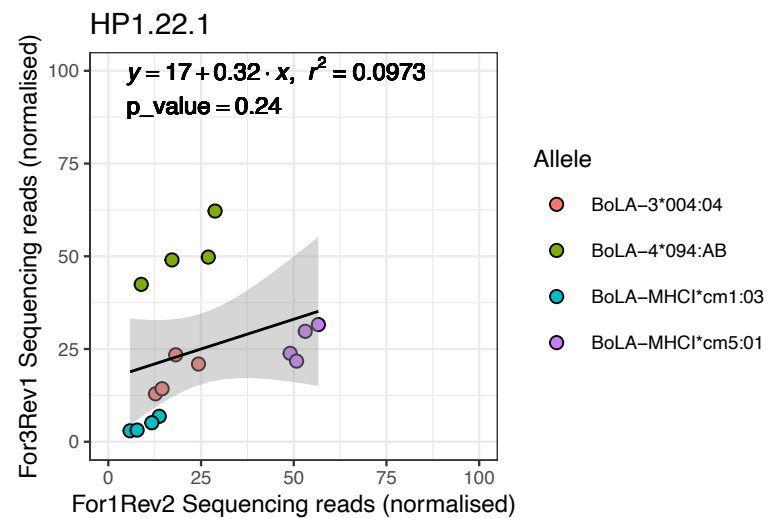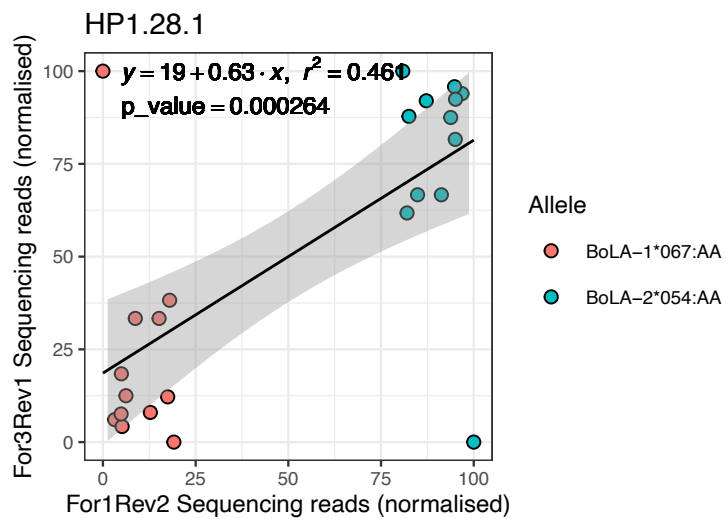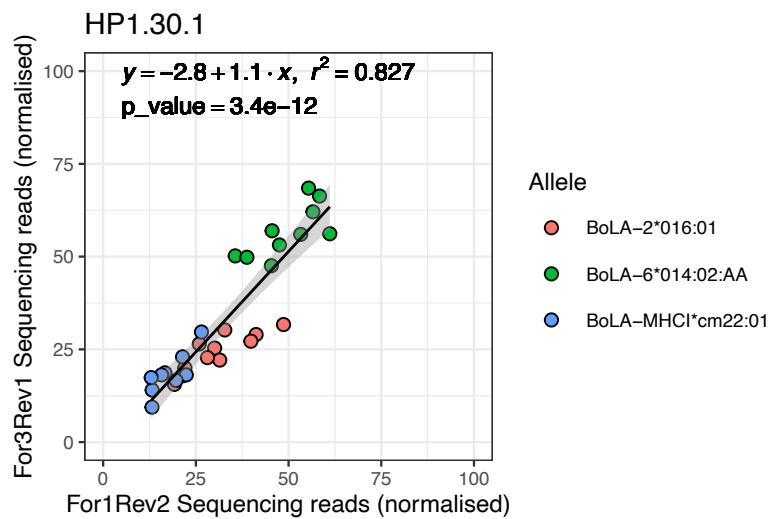

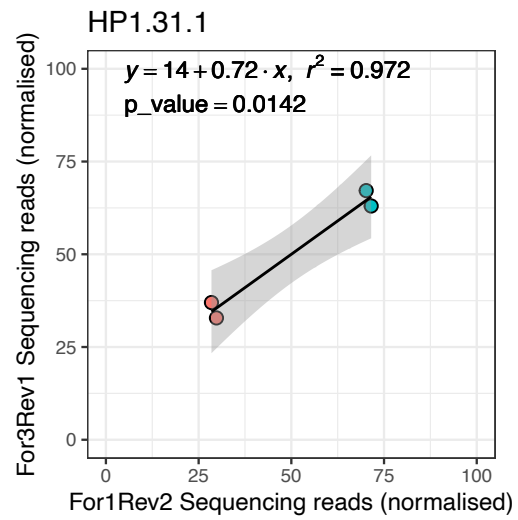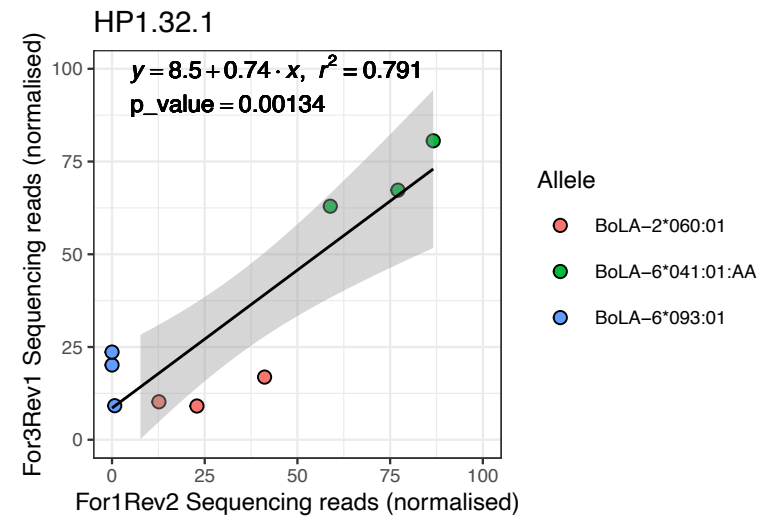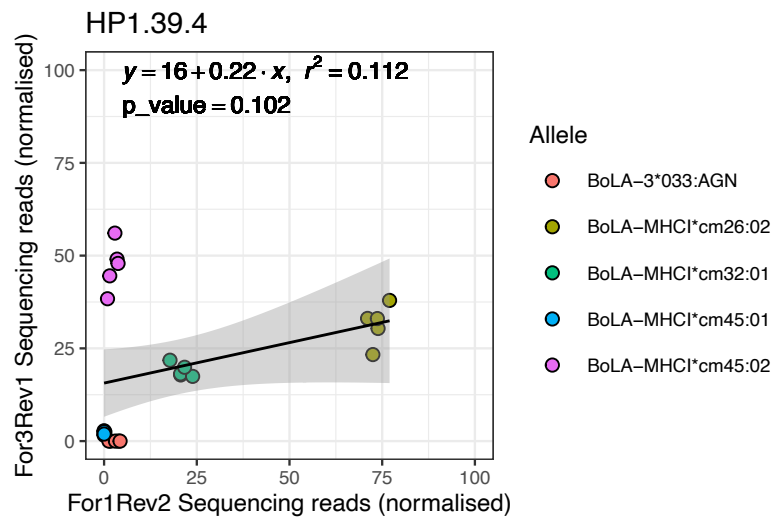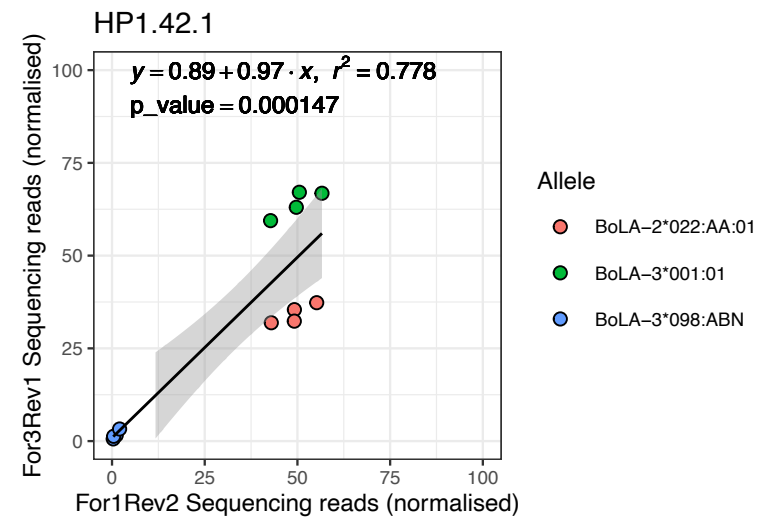

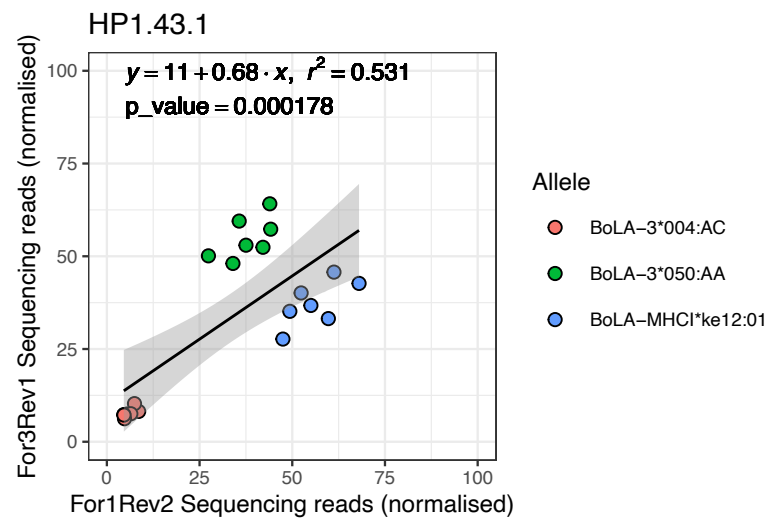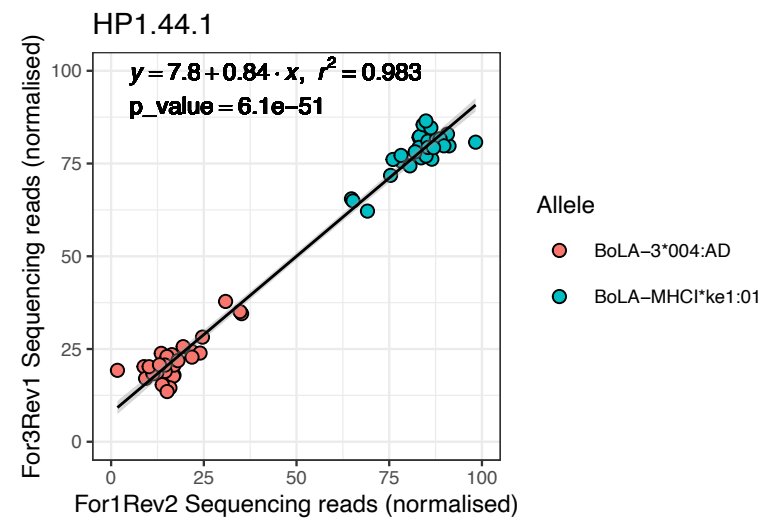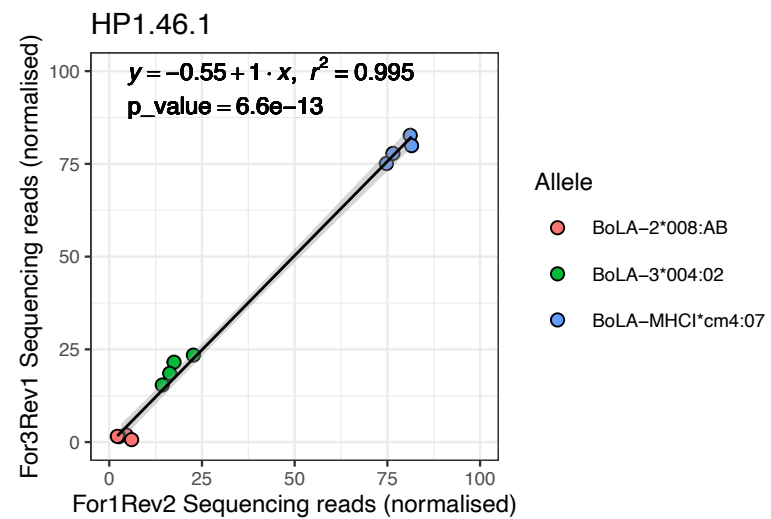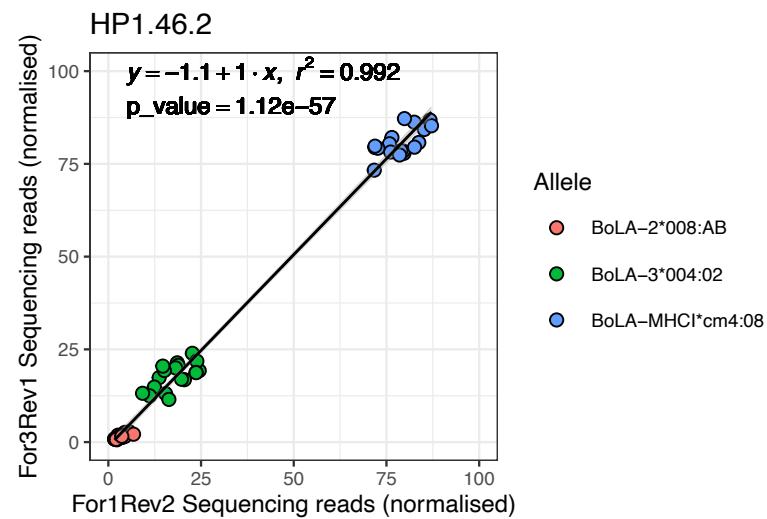

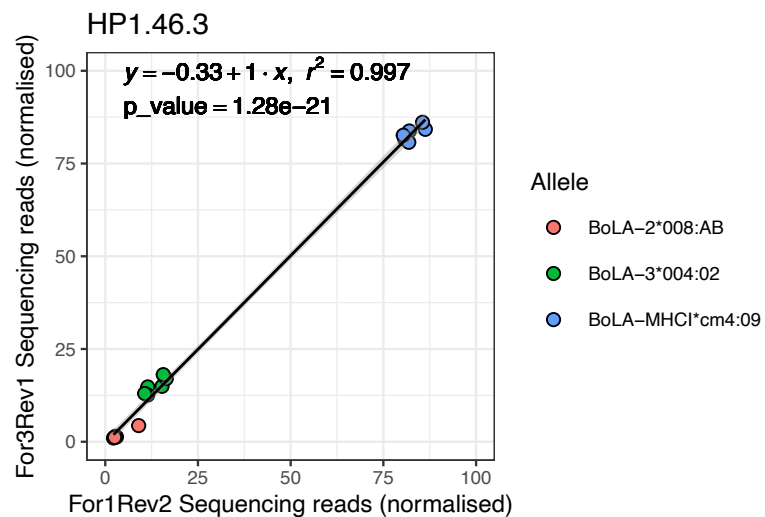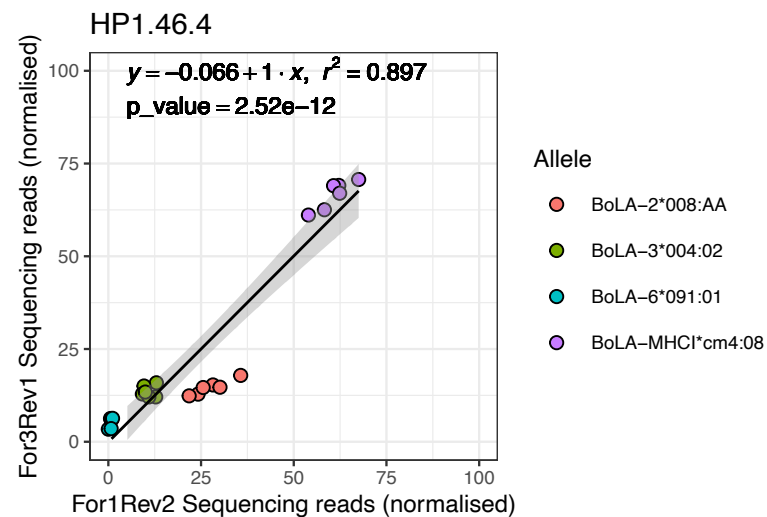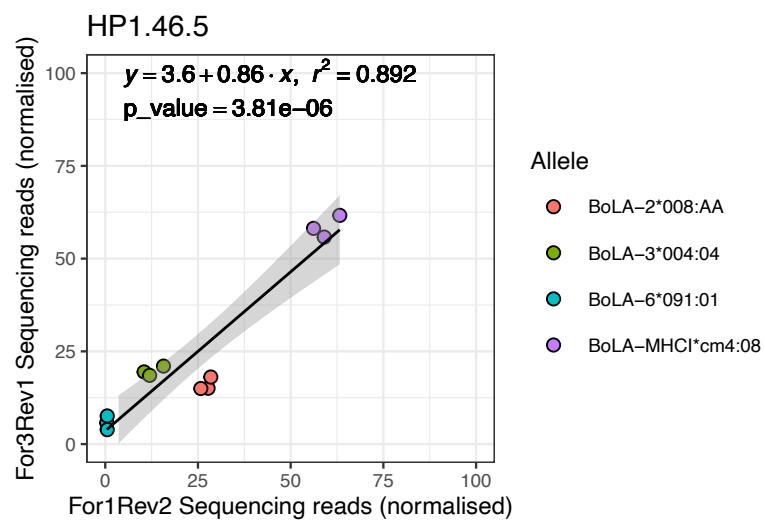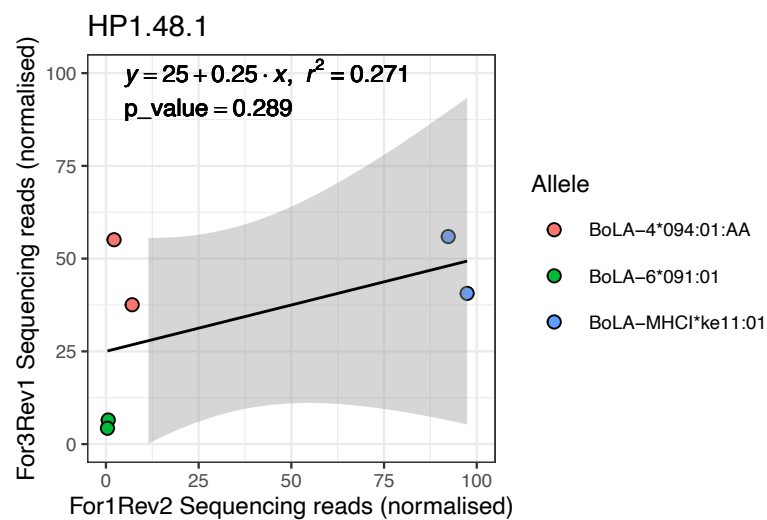

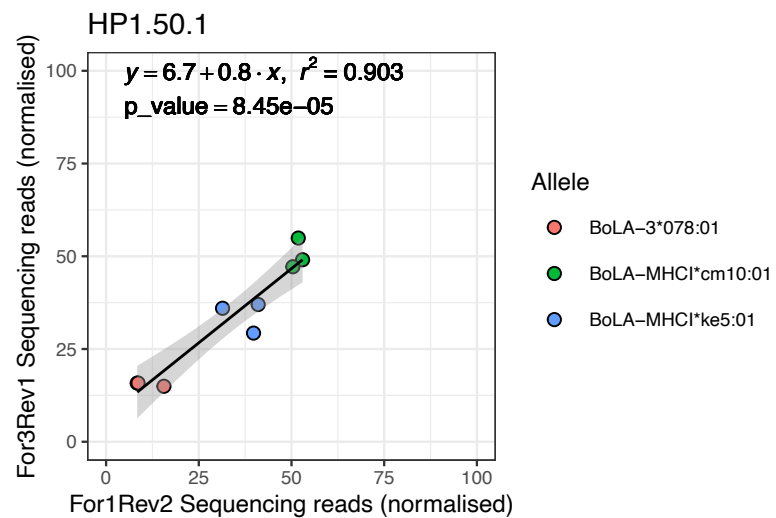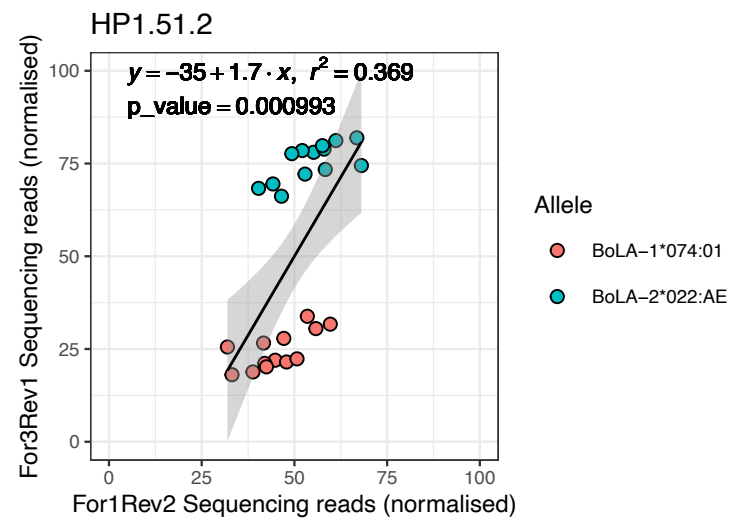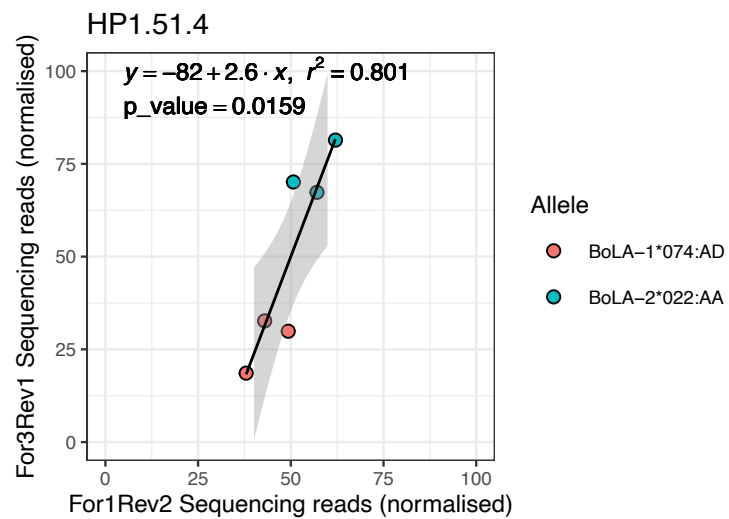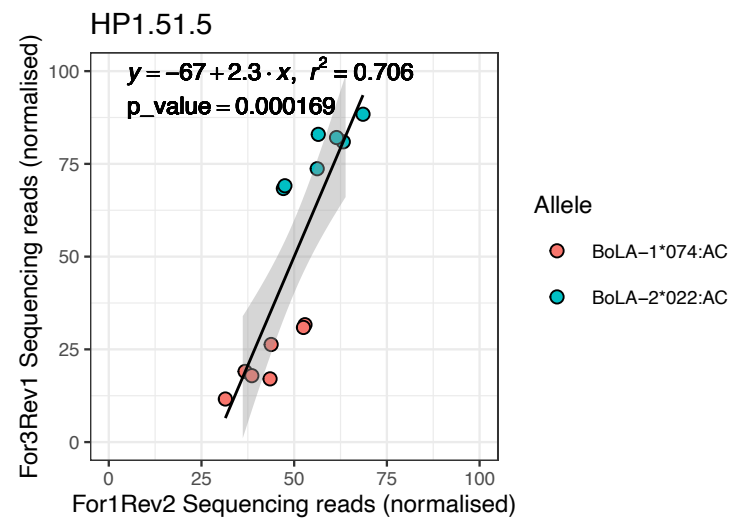

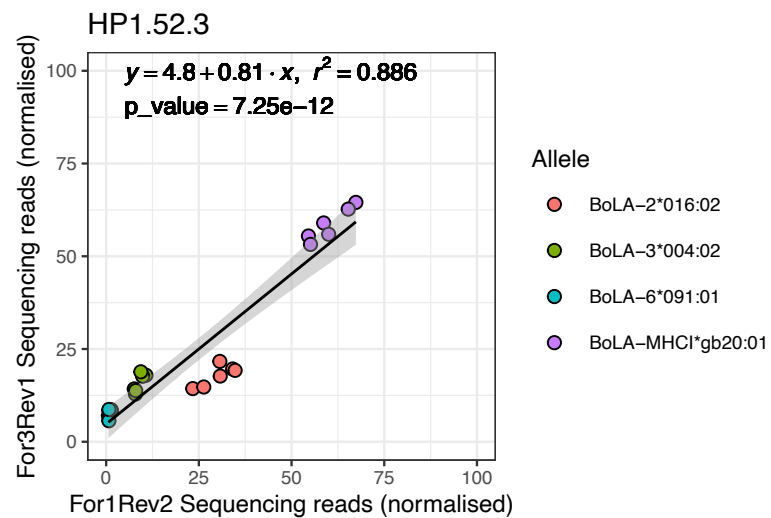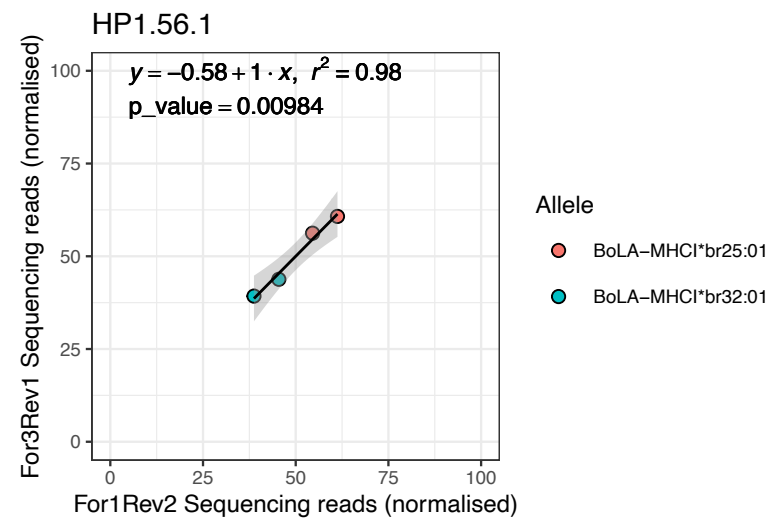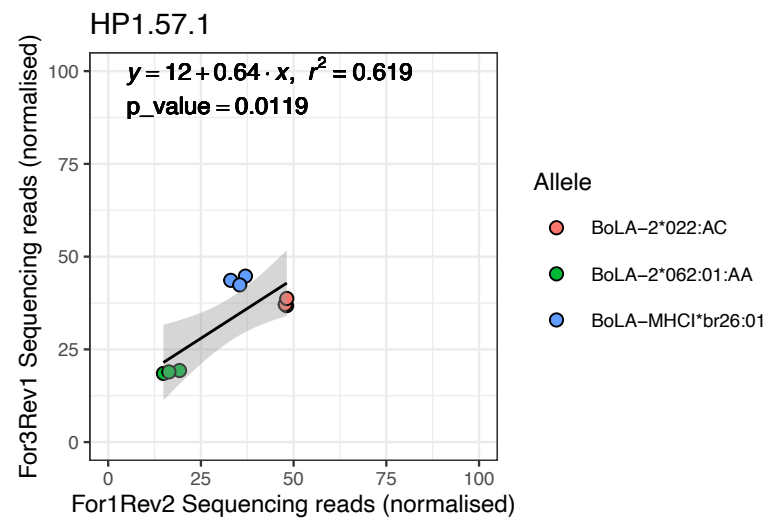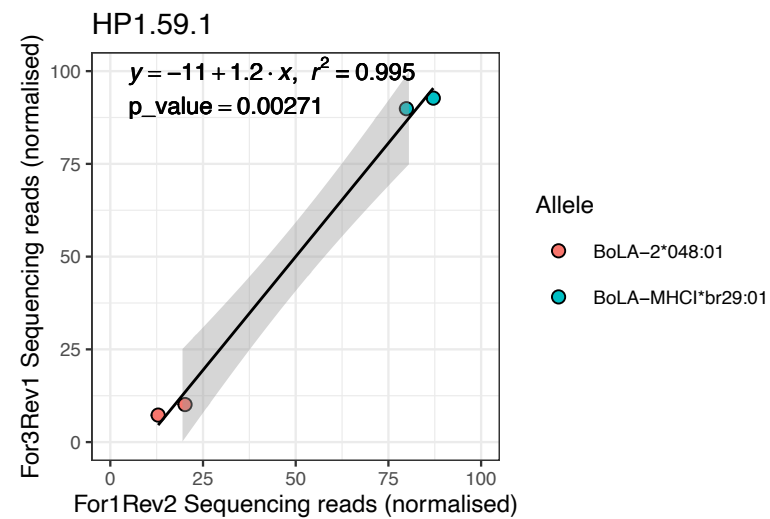

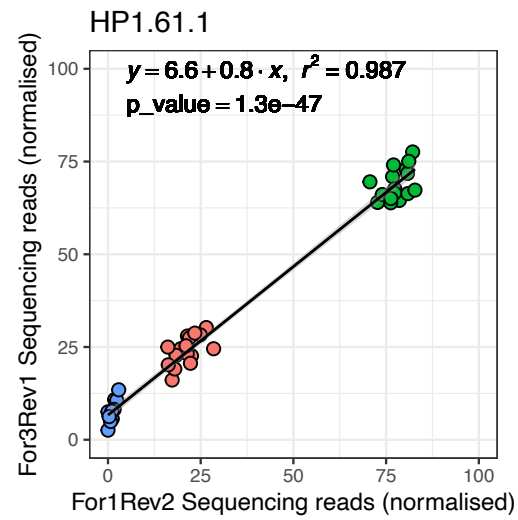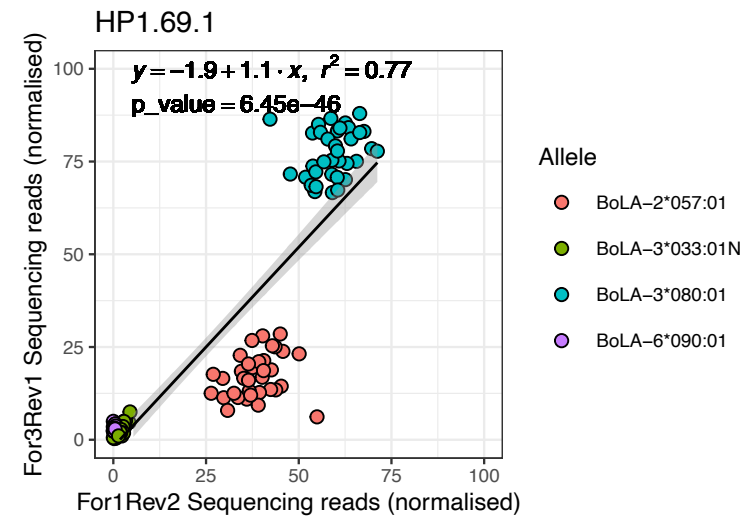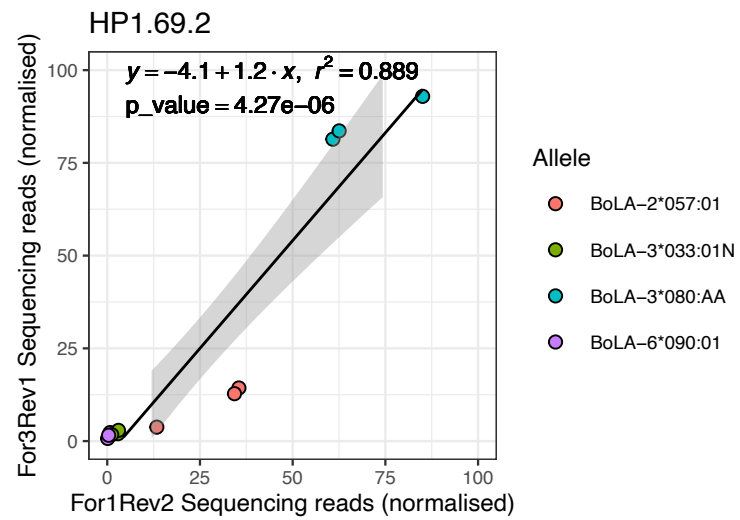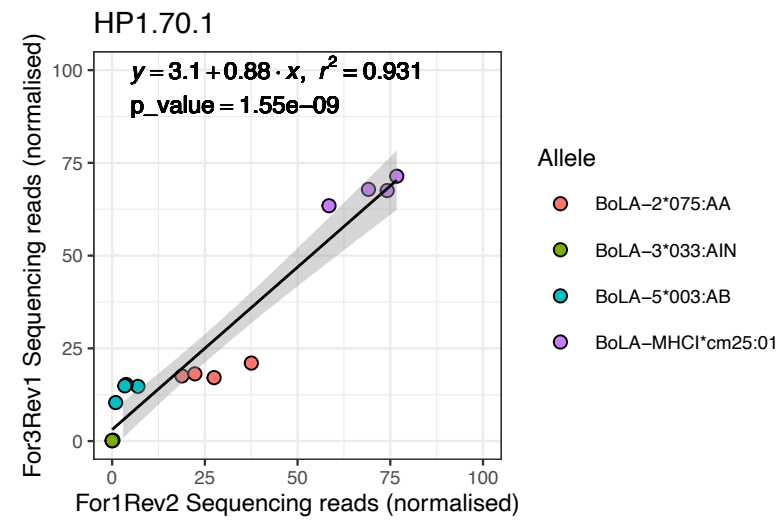

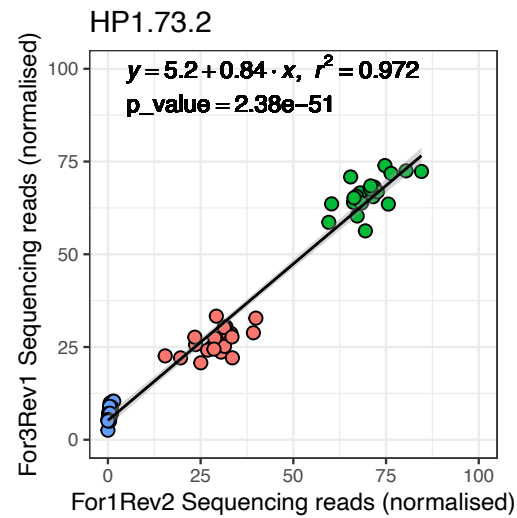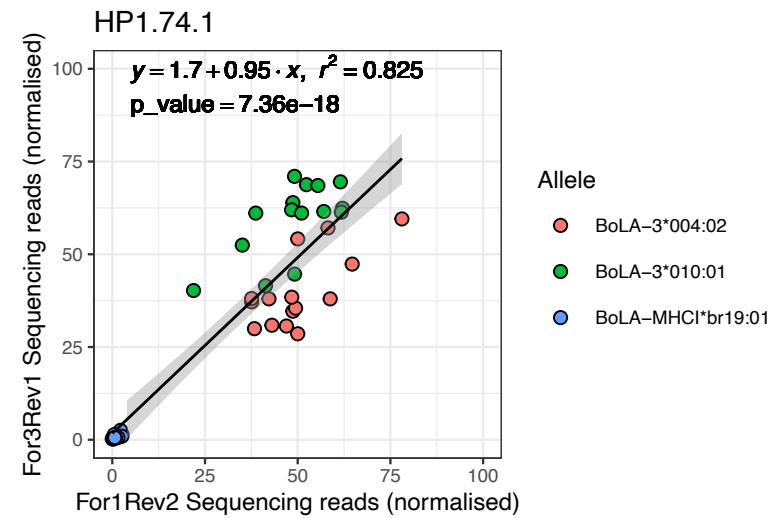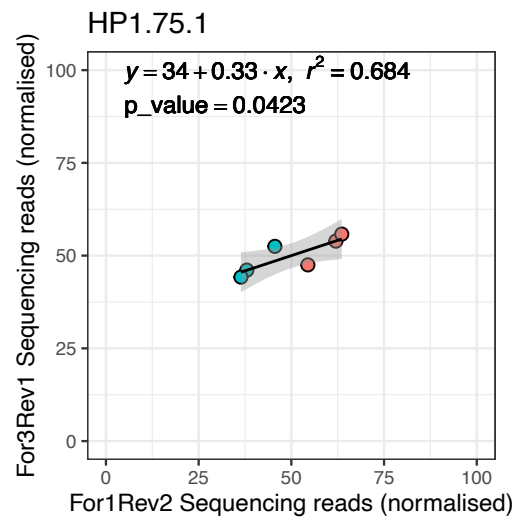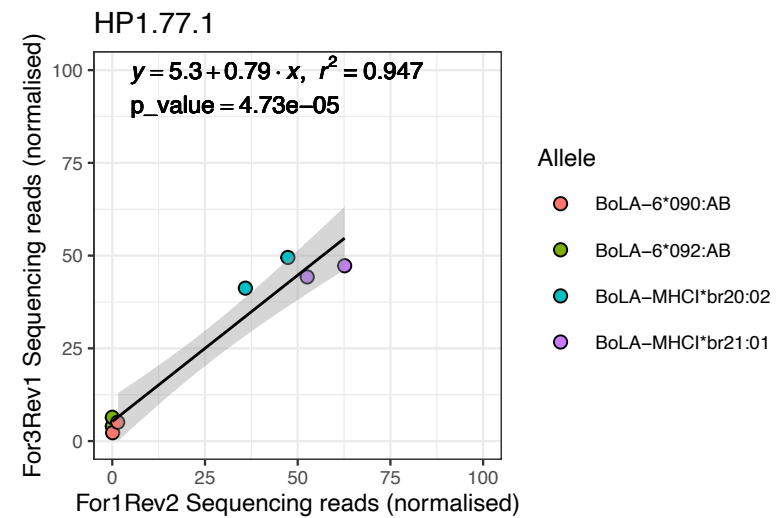

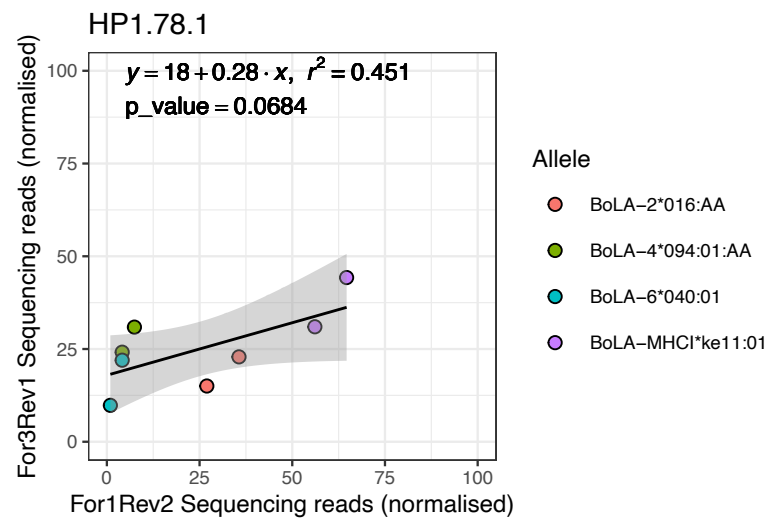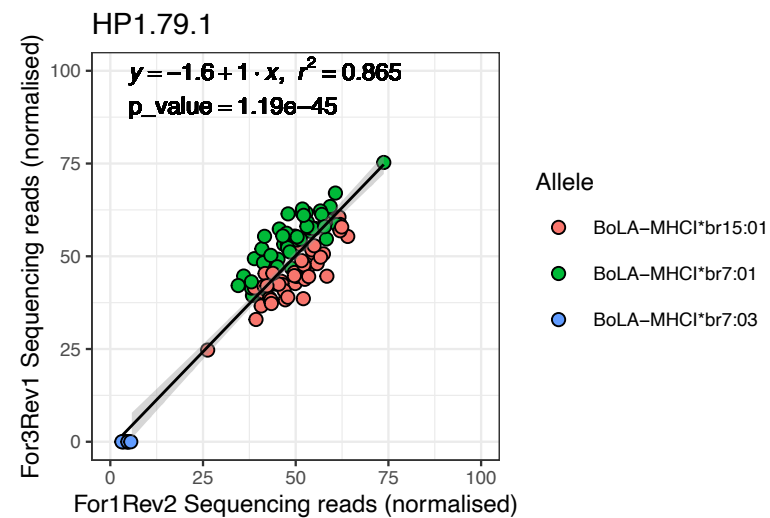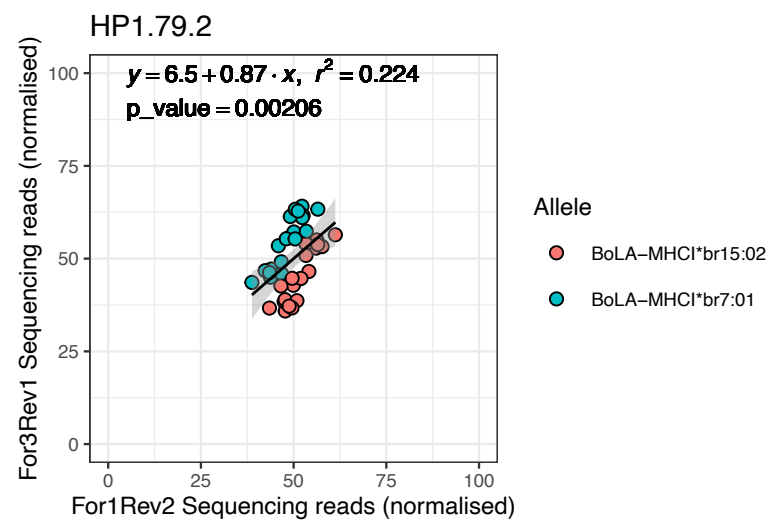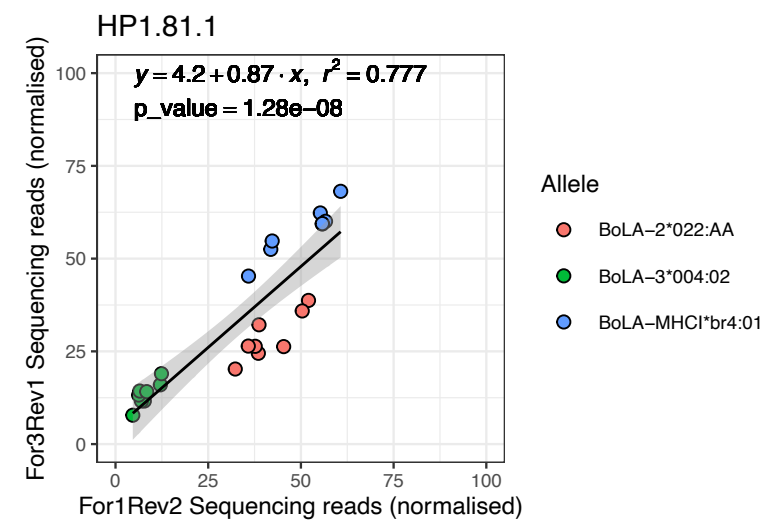

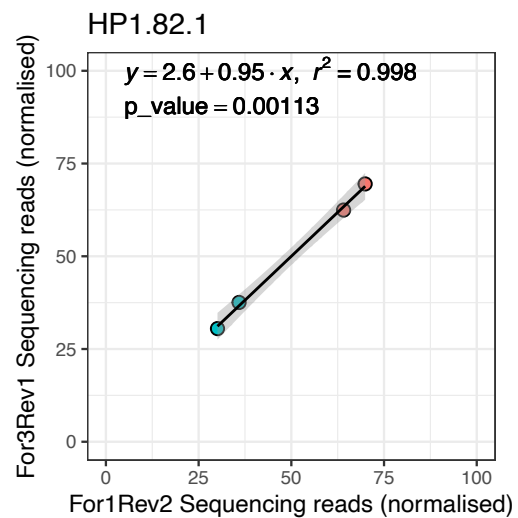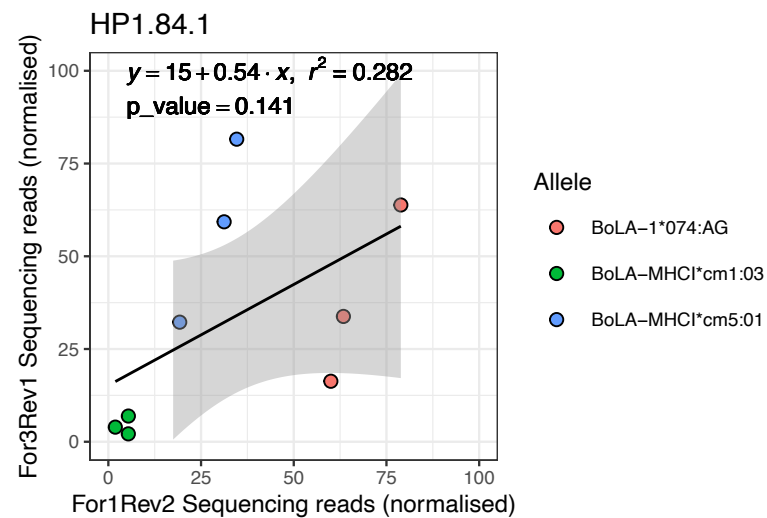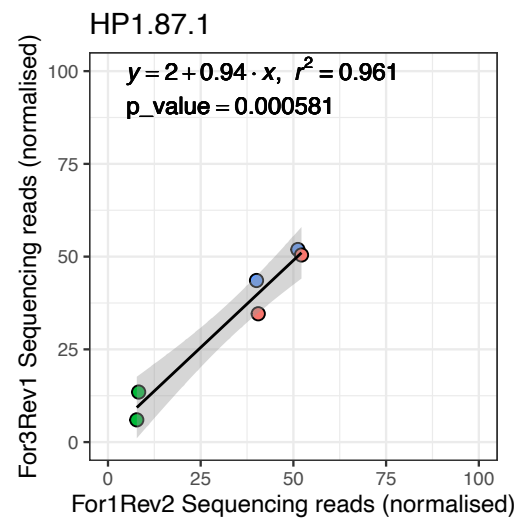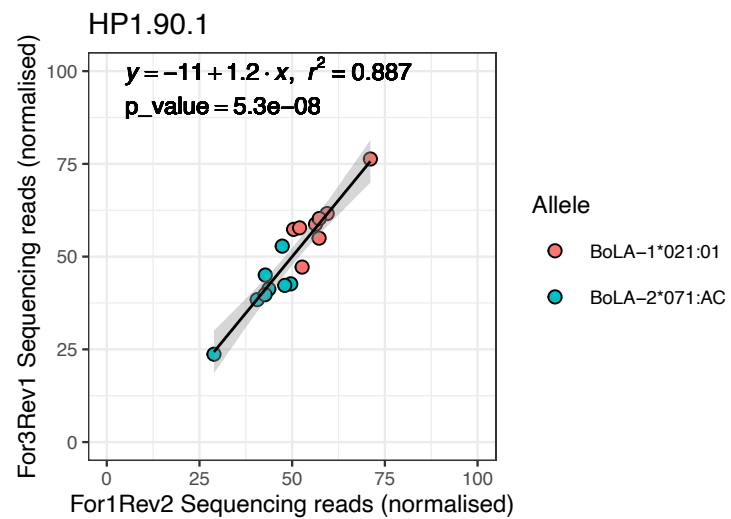

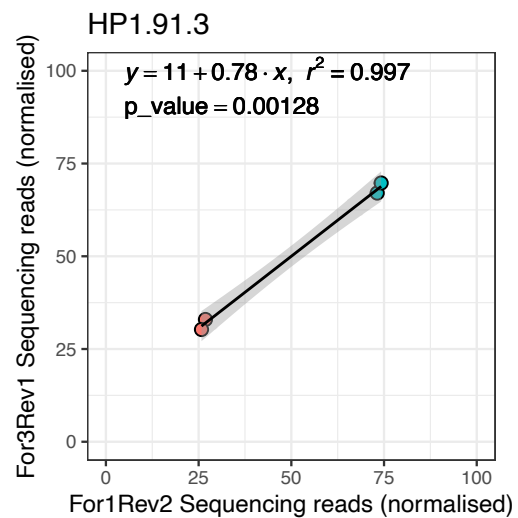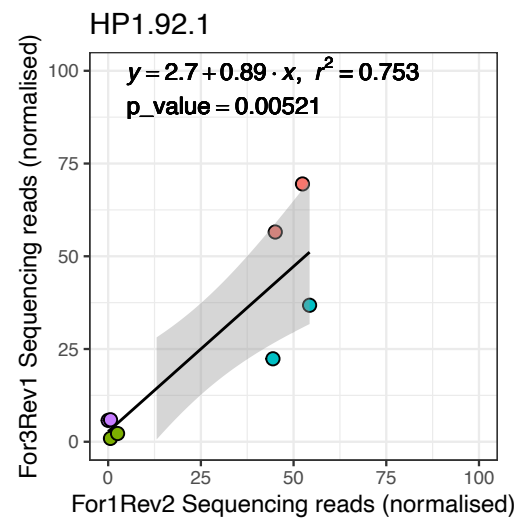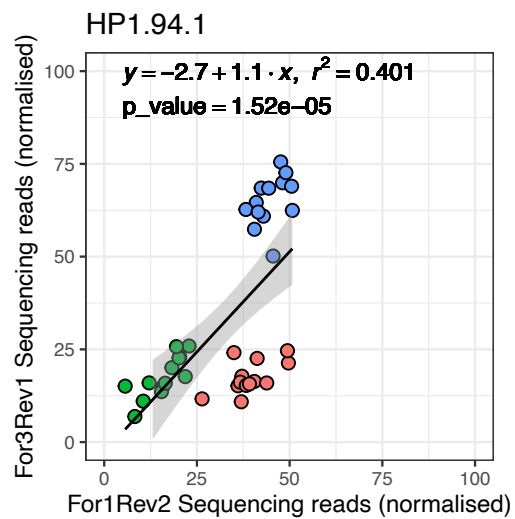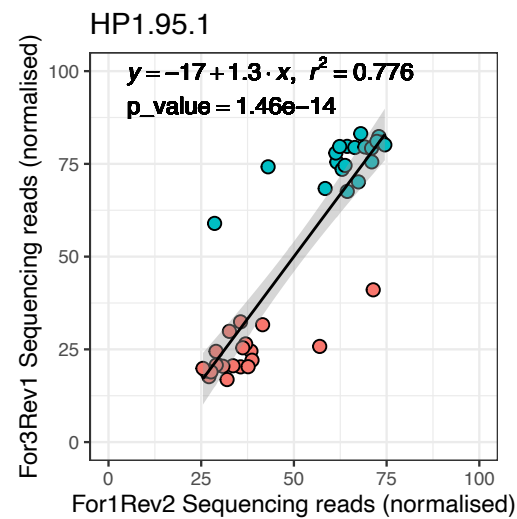

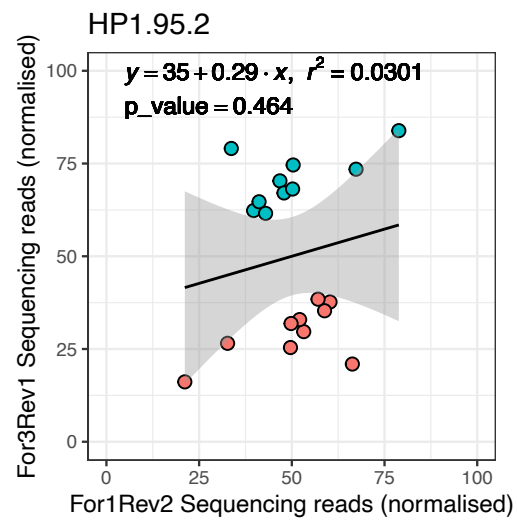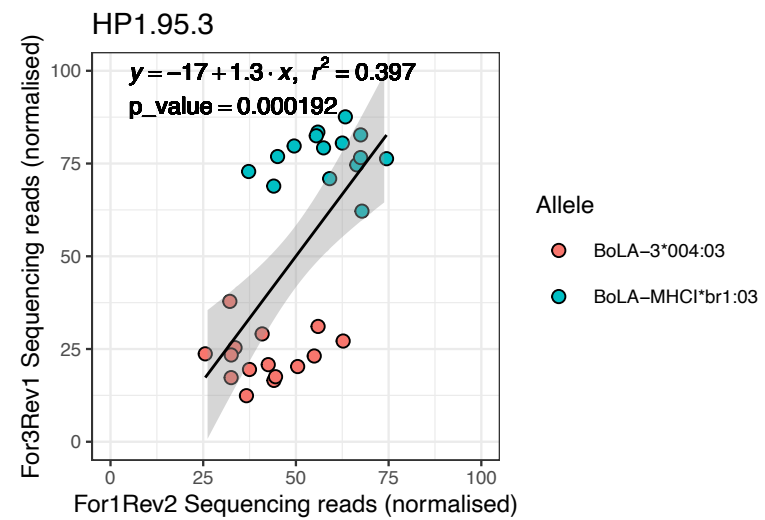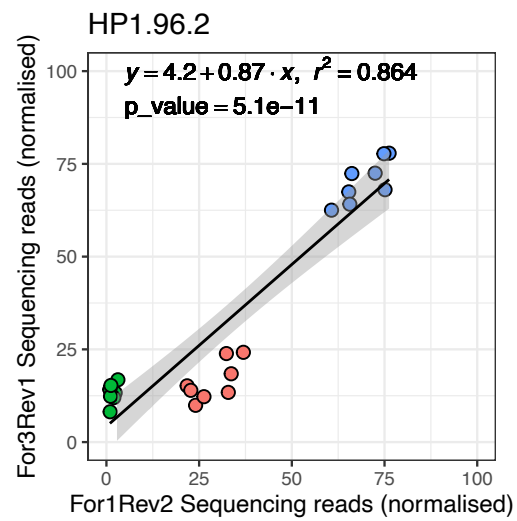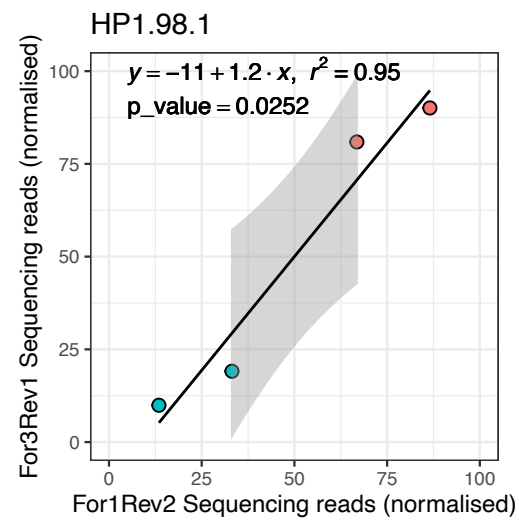

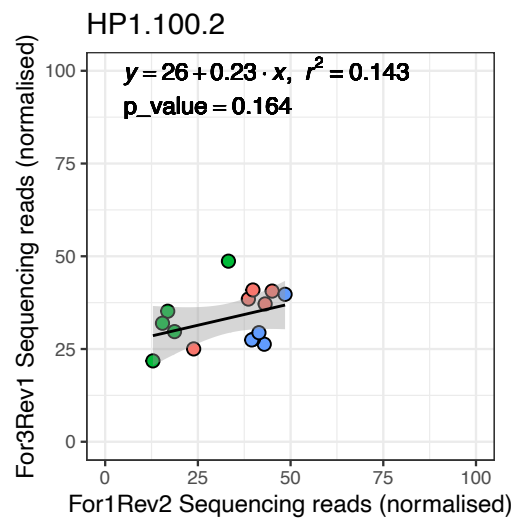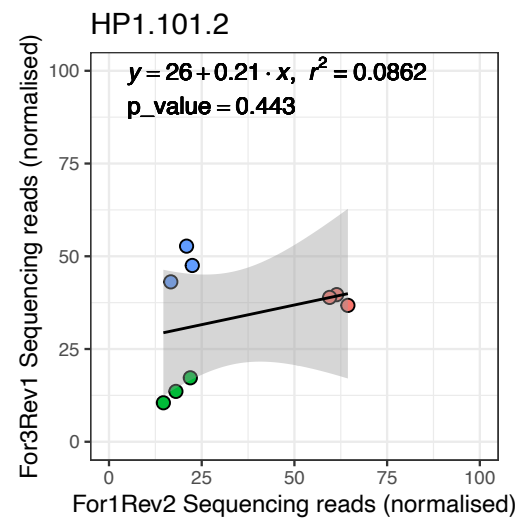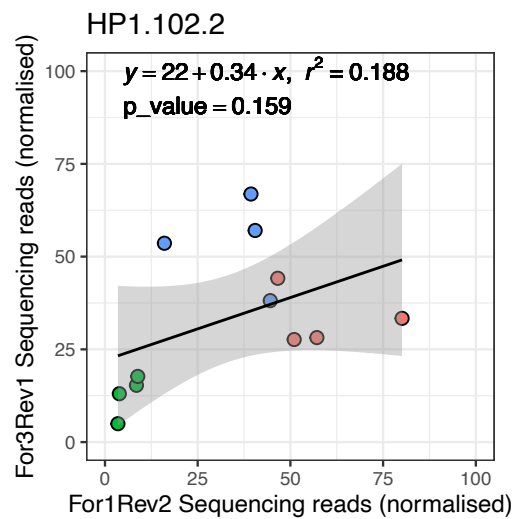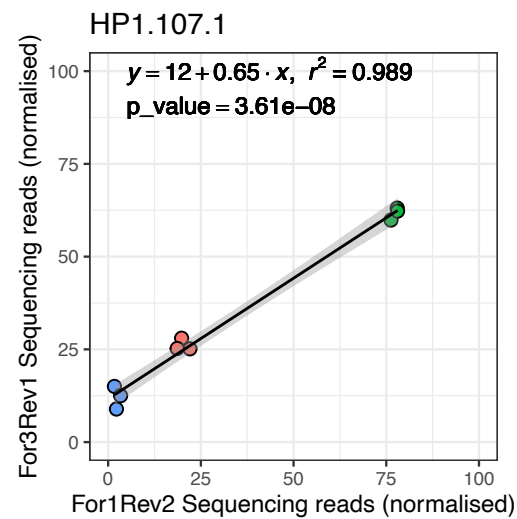

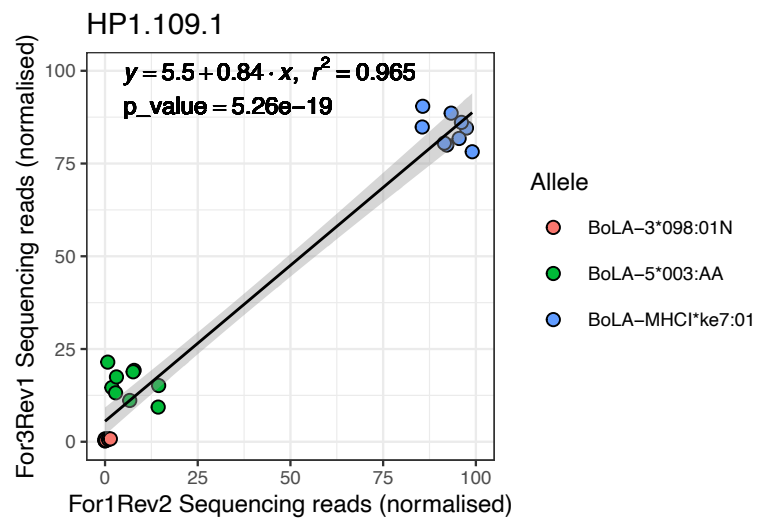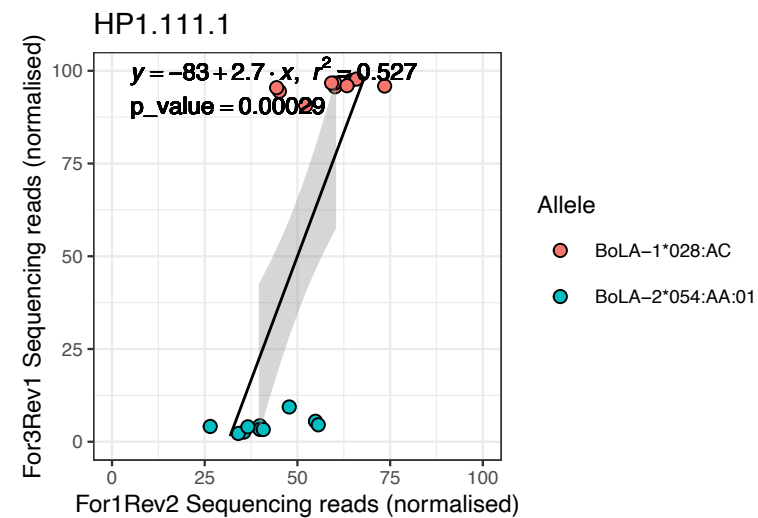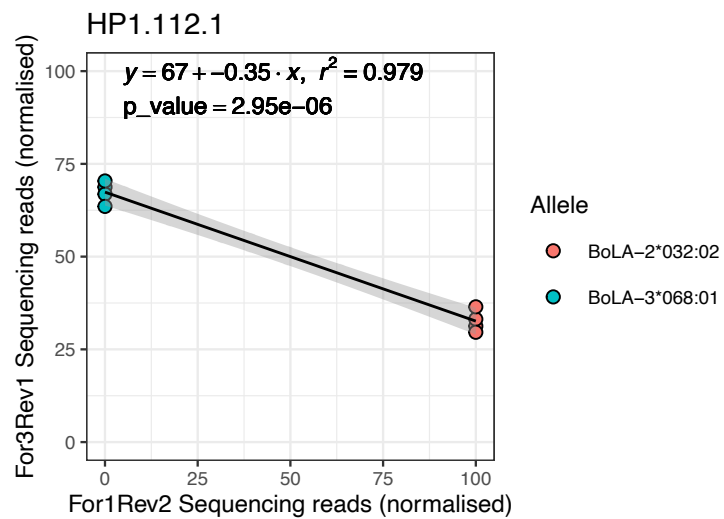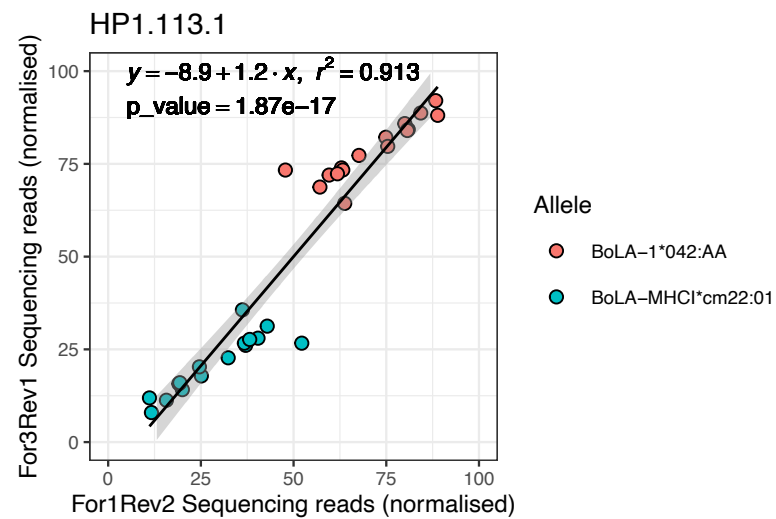

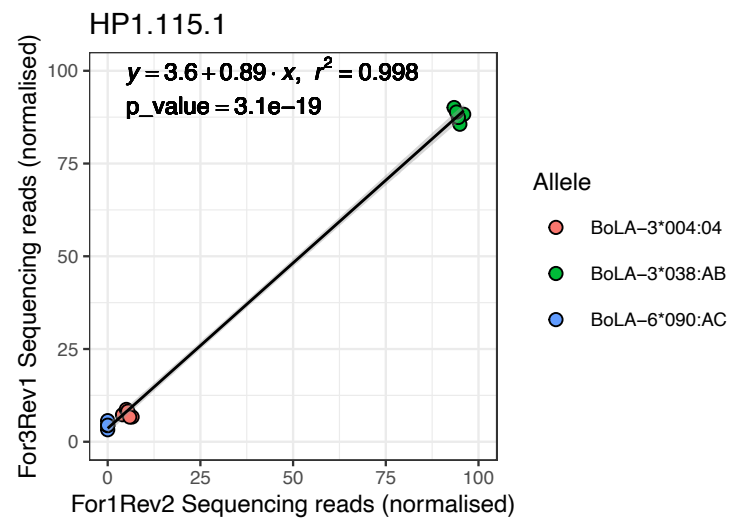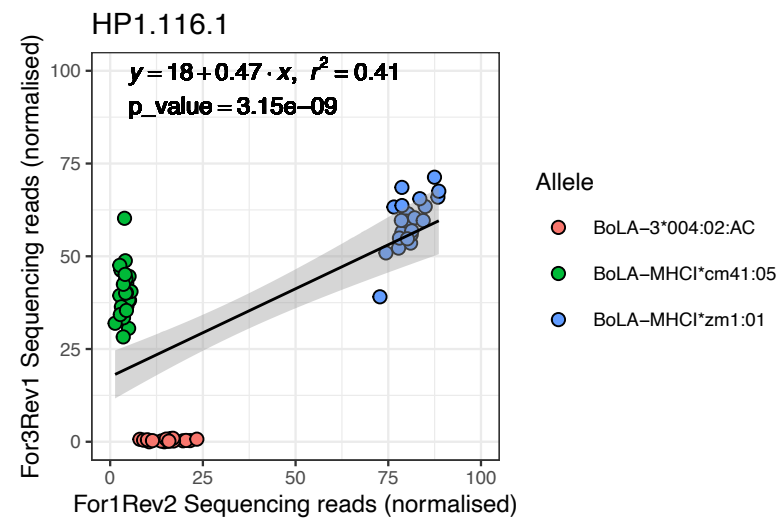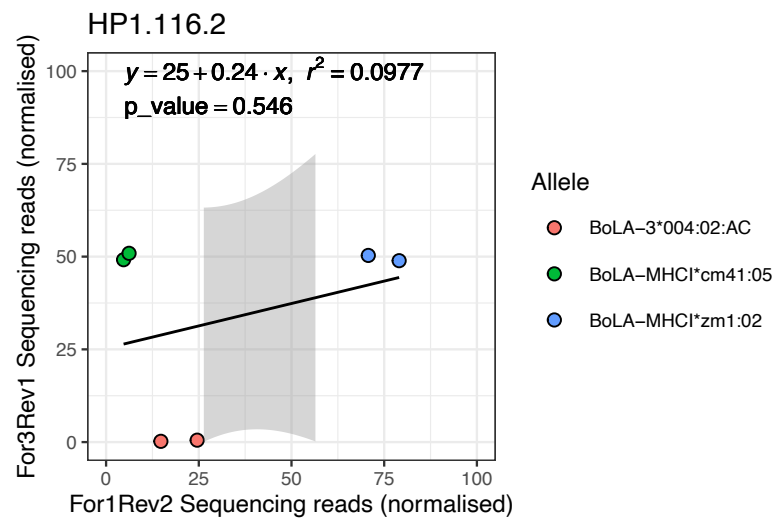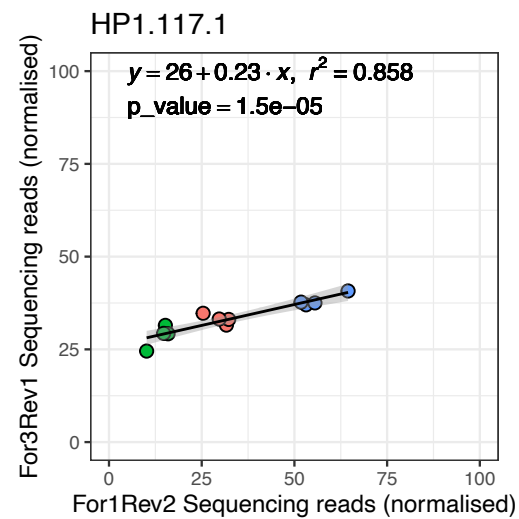

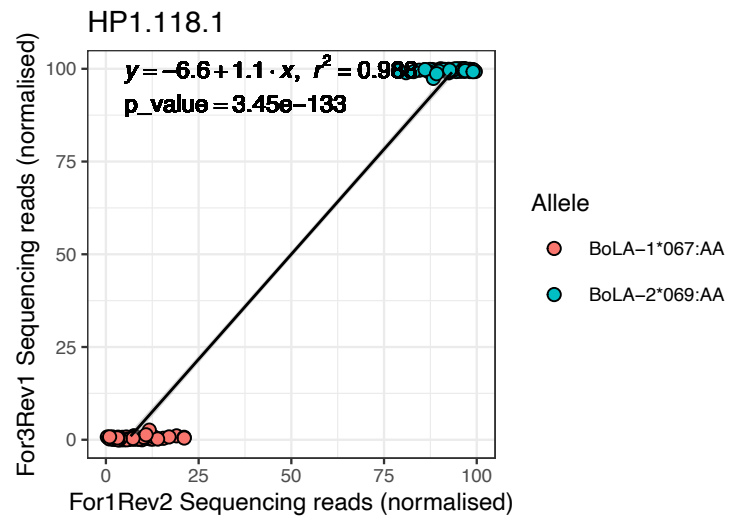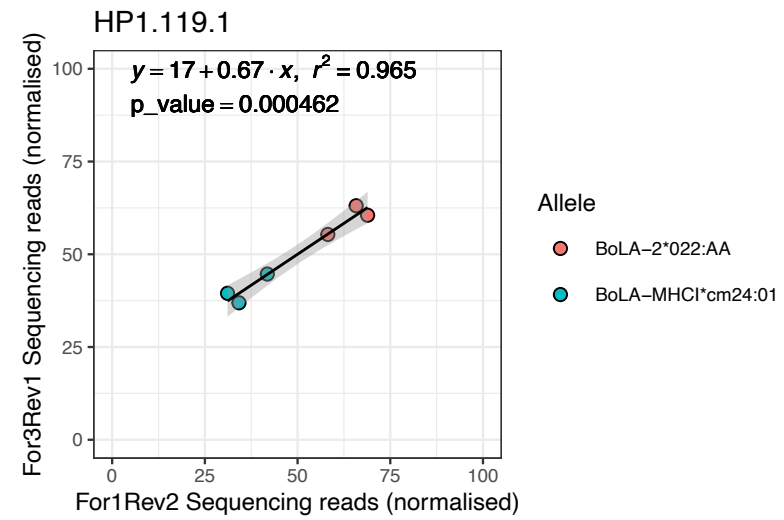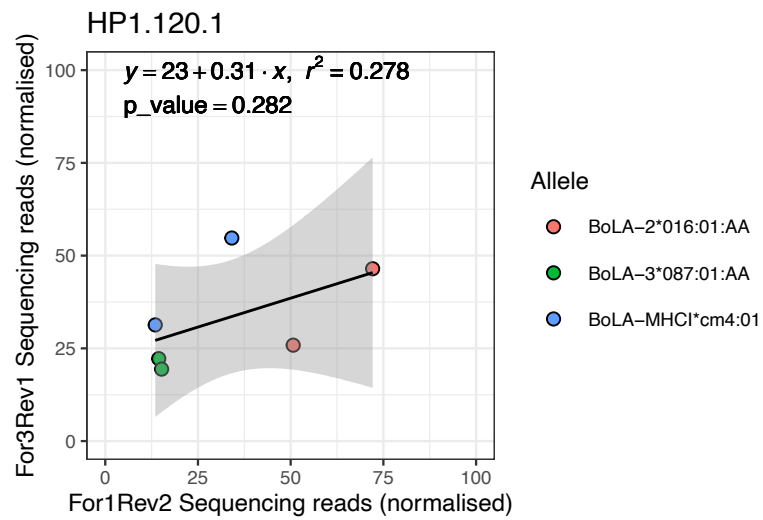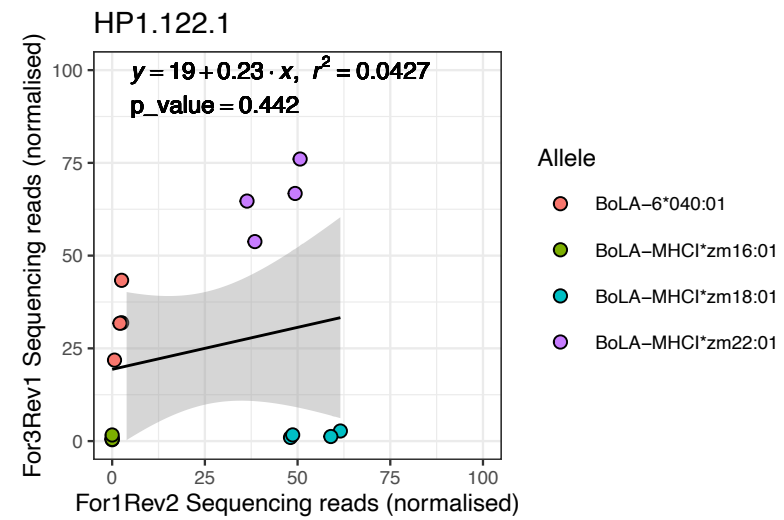

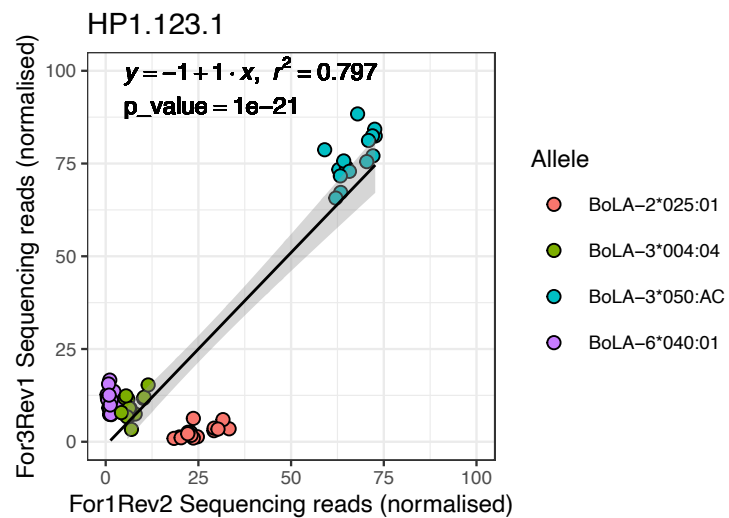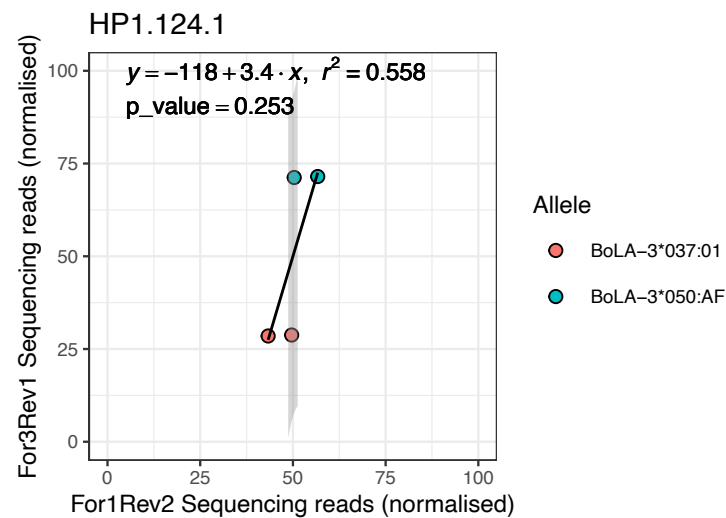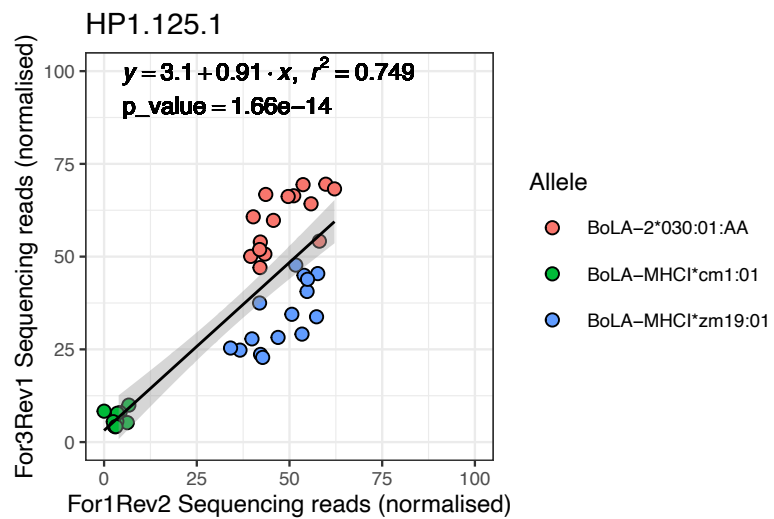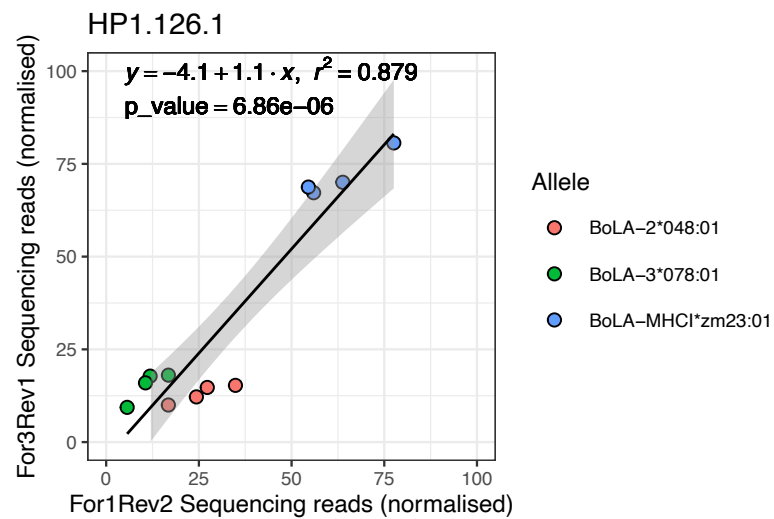

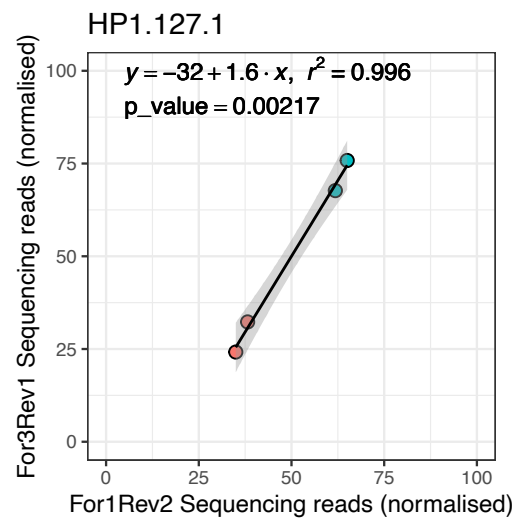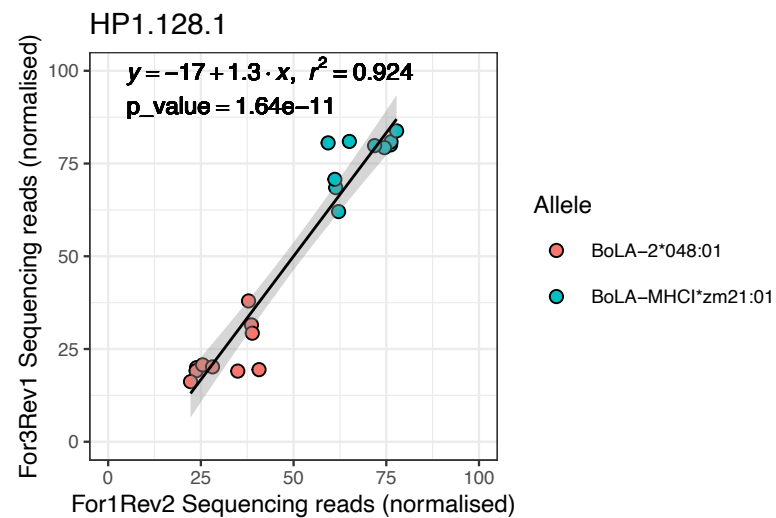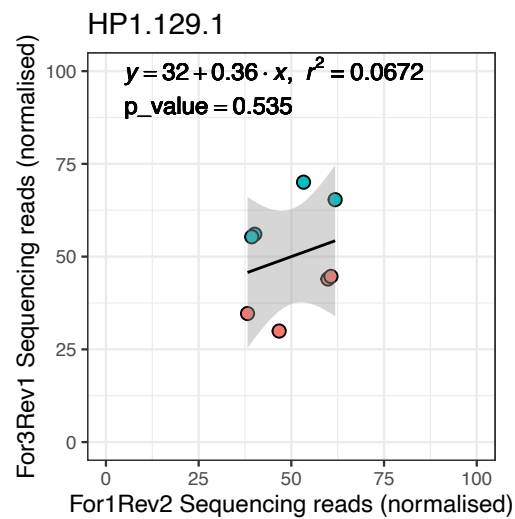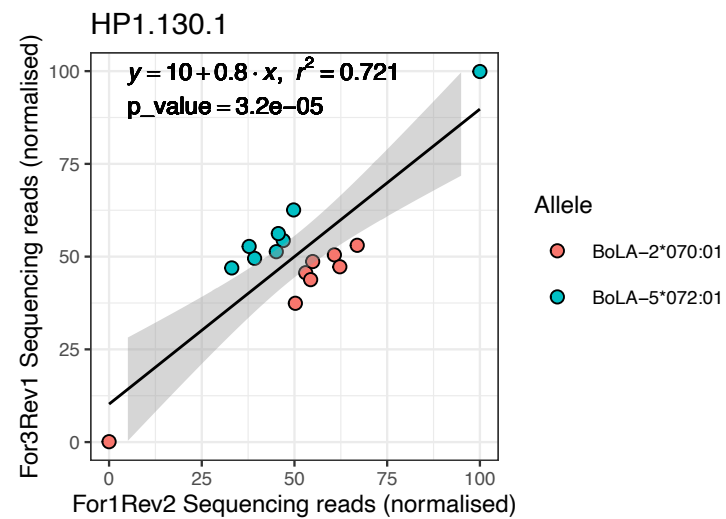

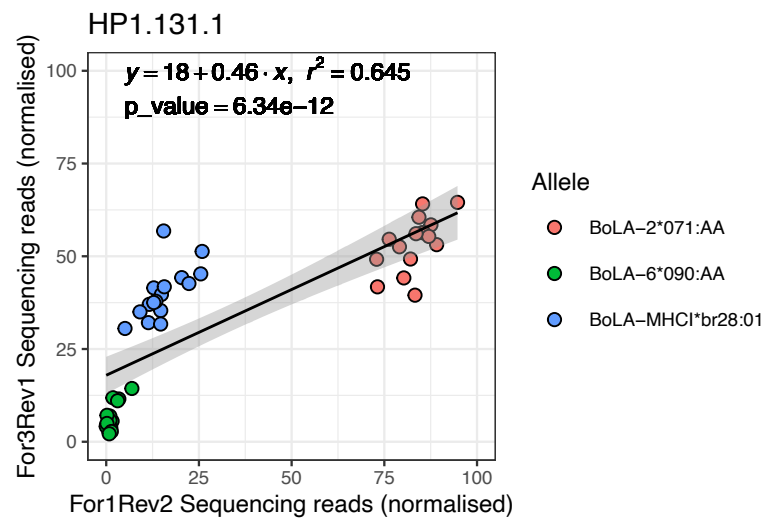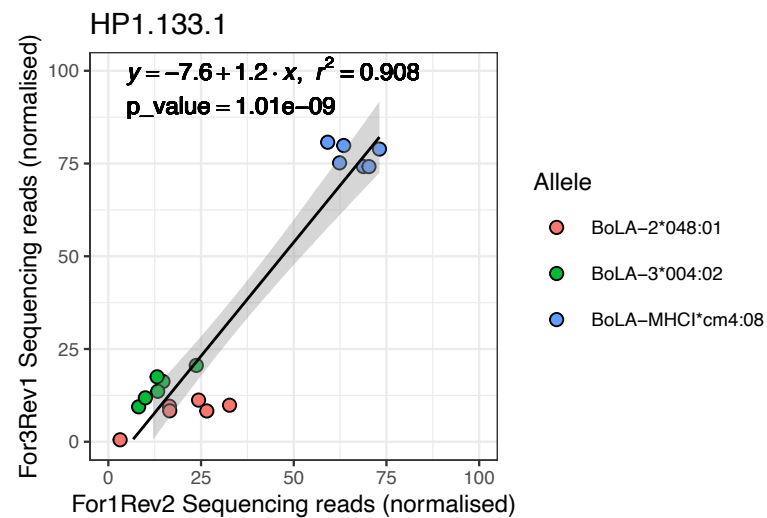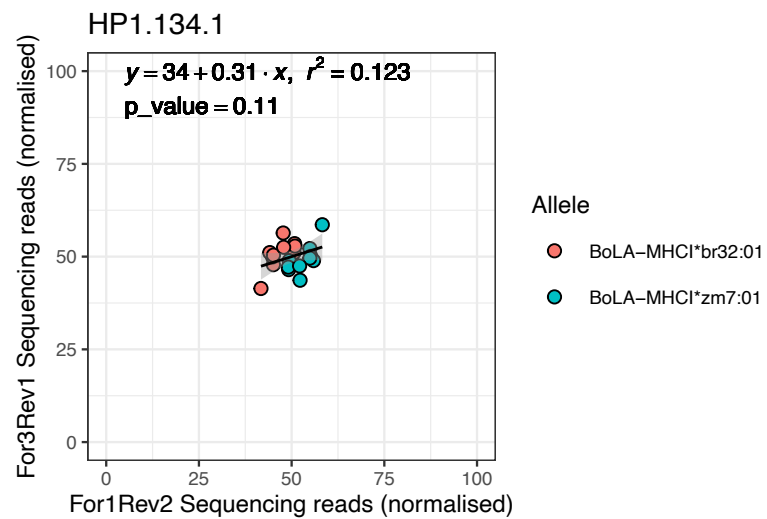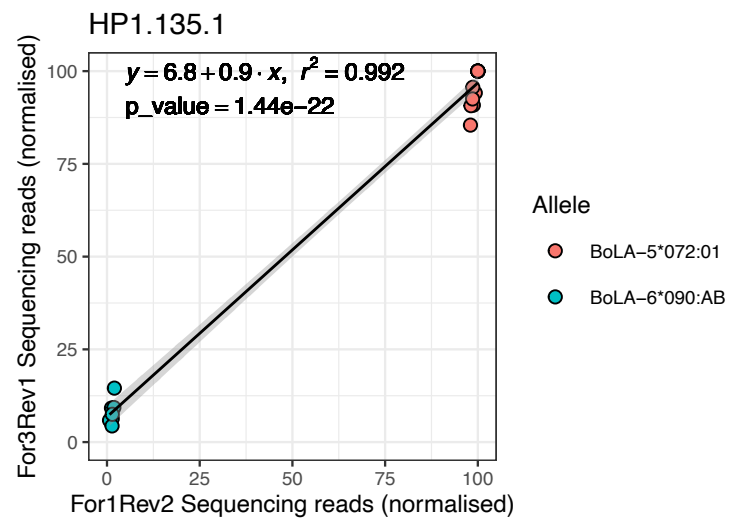

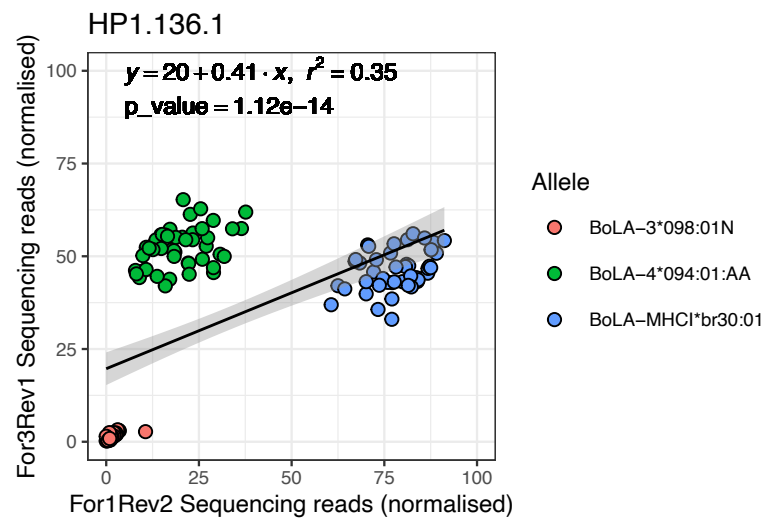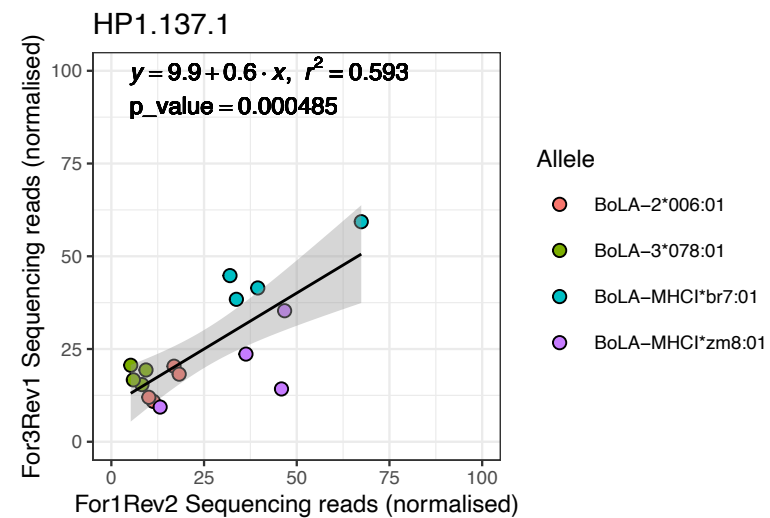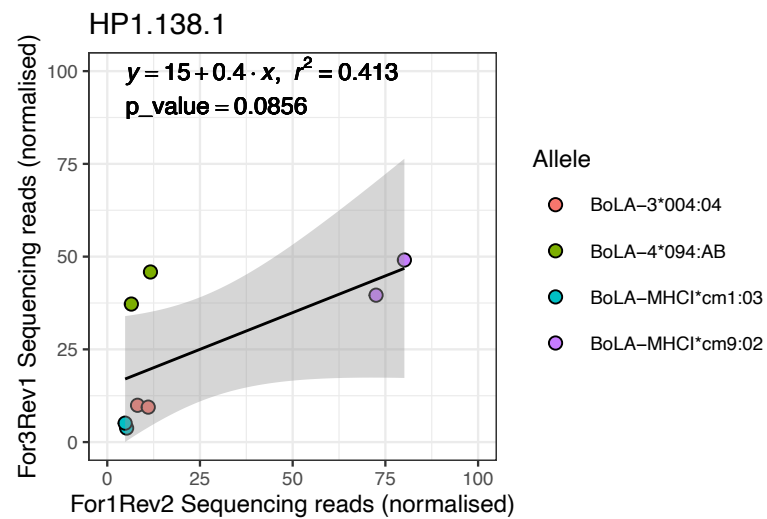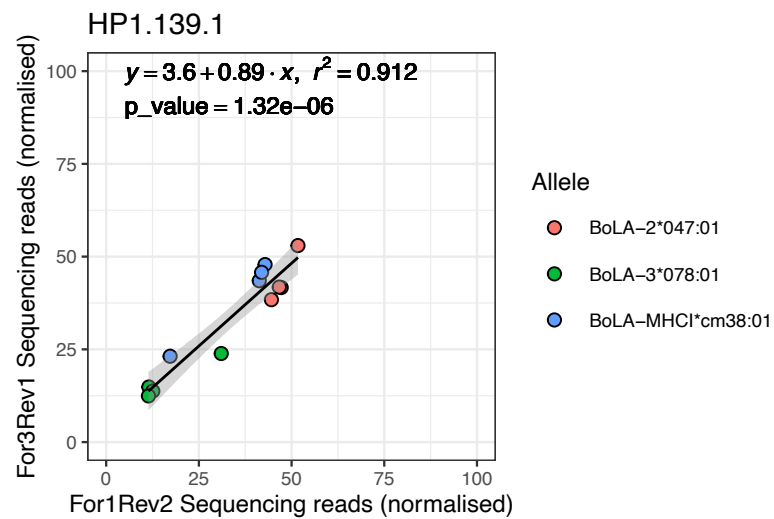

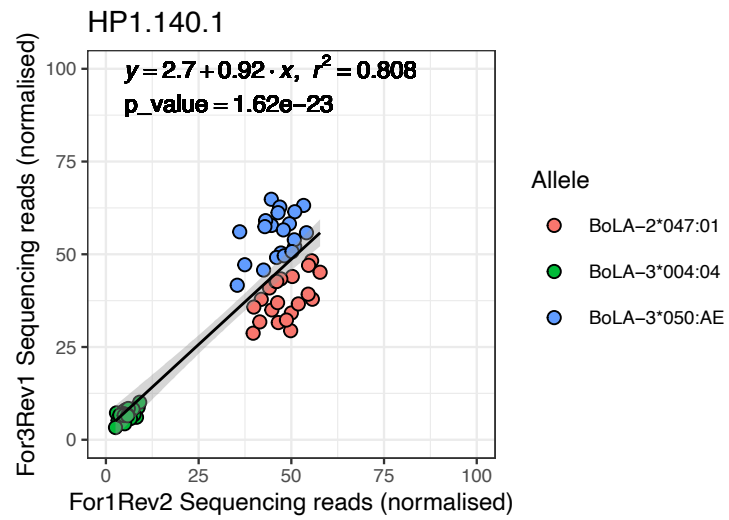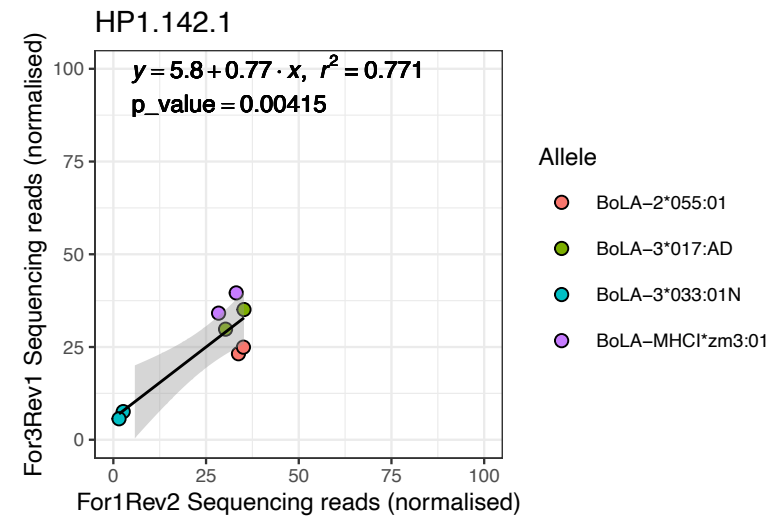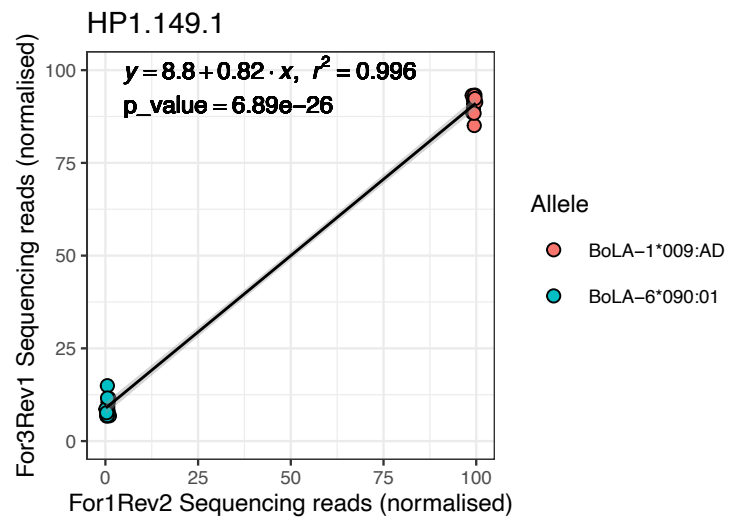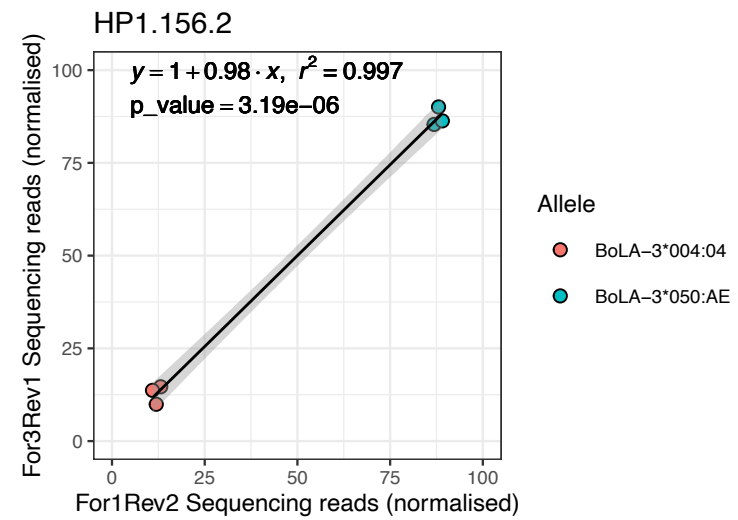

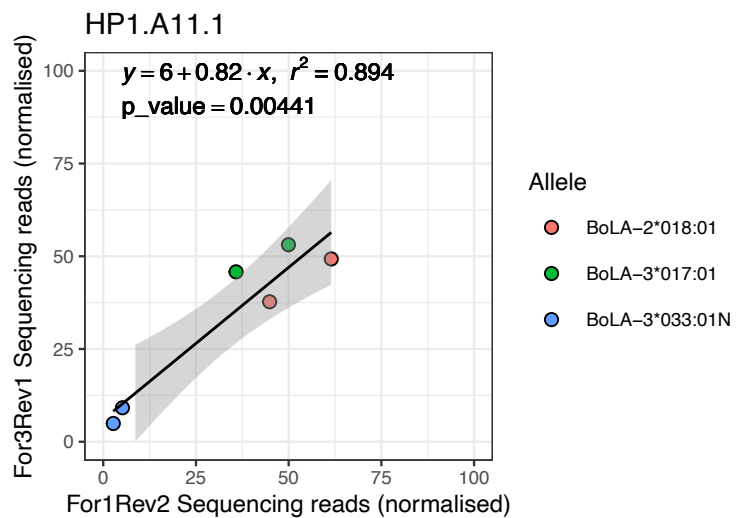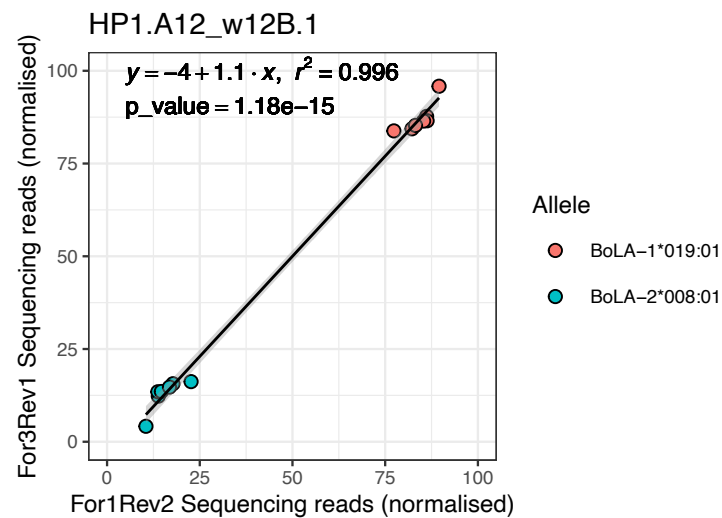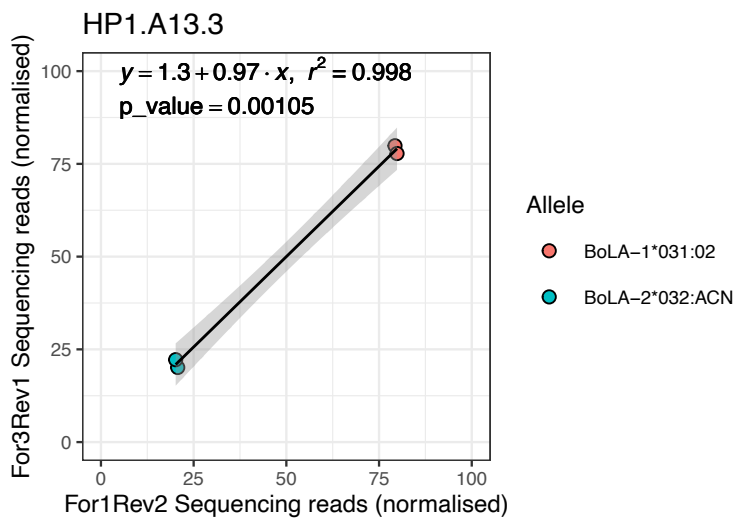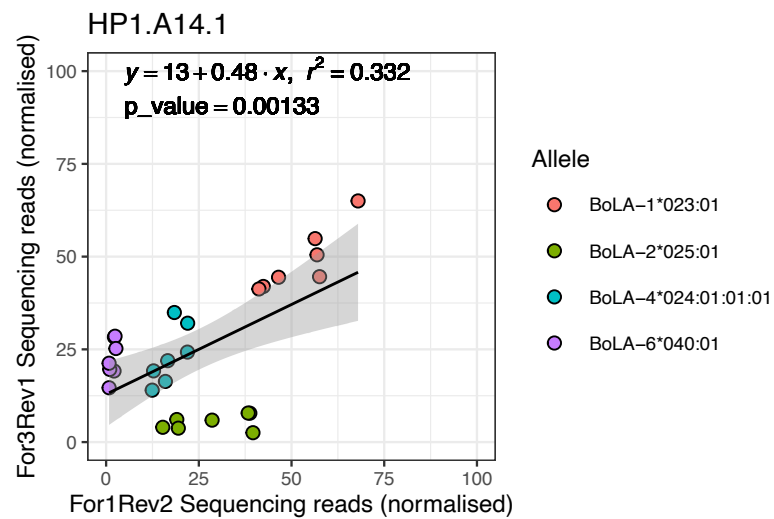

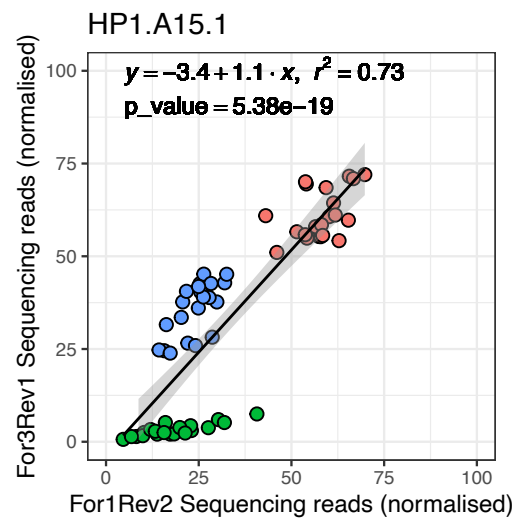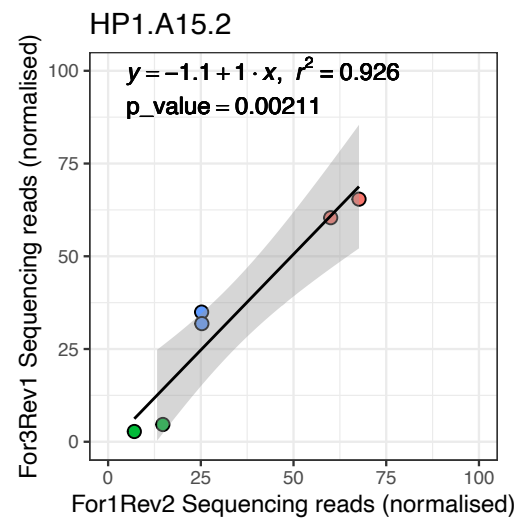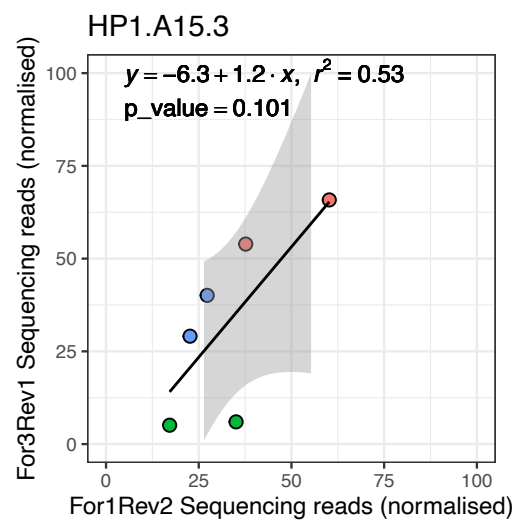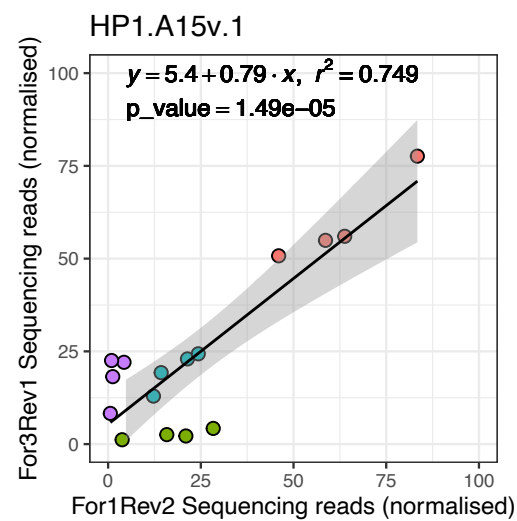

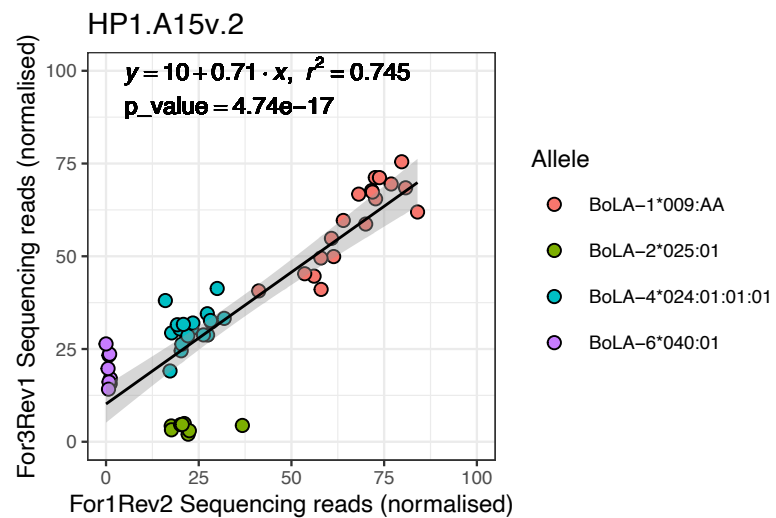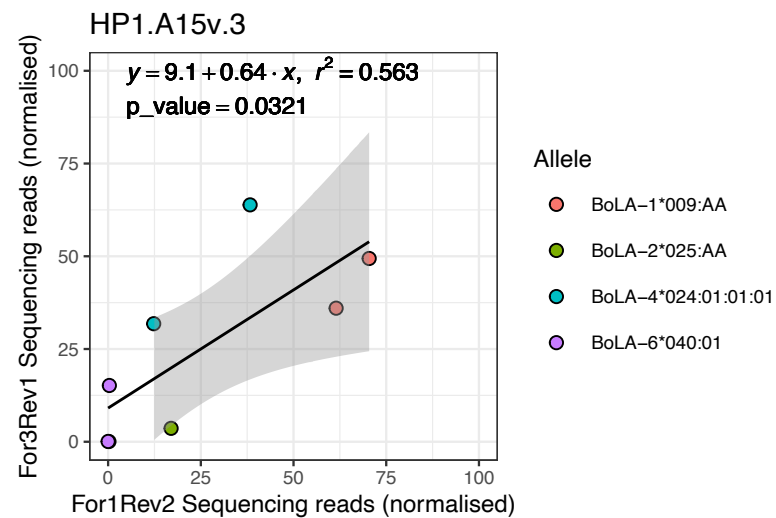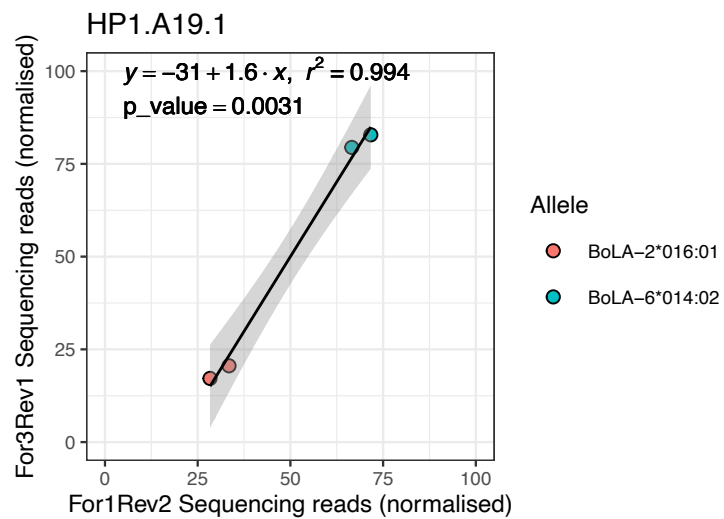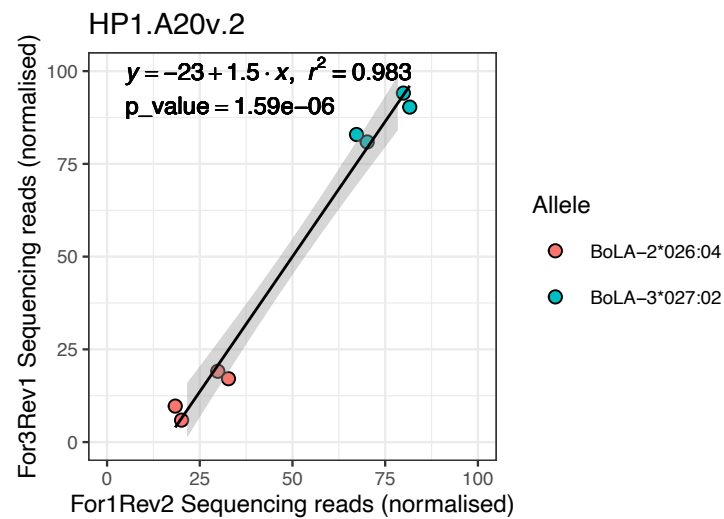

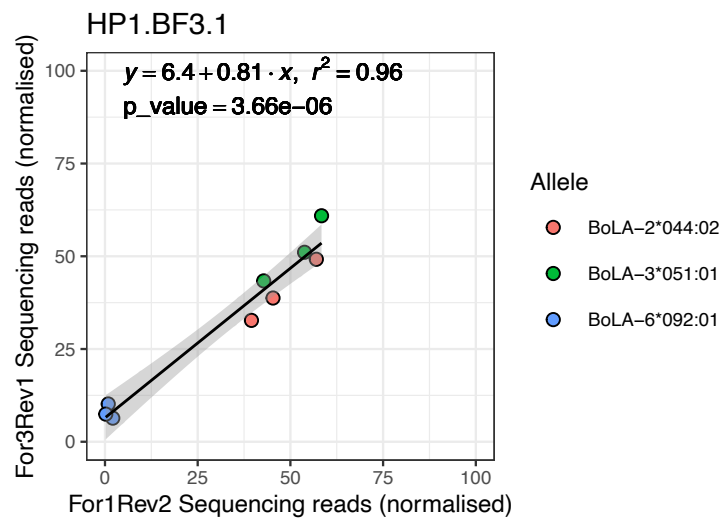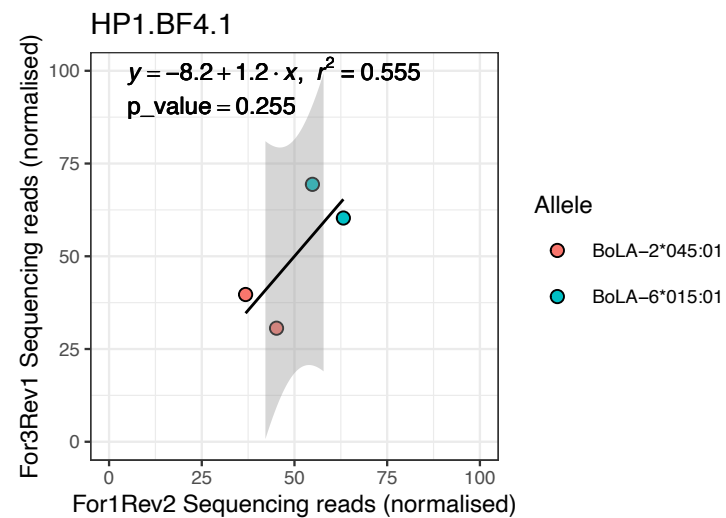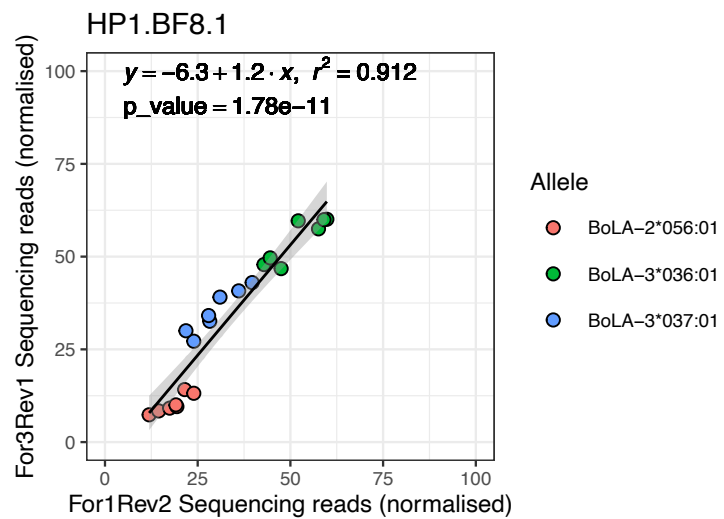

Supplement: Supplementary file 4 — Supplementary Data 4 Scatterplot analysis of the read frequency observed with the For1/Rev2 and For3/Rev1 MHCI PCR reactions for each haplotype identified in the Zambian cohort. Scatterplots are used to illustrate the correspondence between the two independent PCRs for the normalised read frequency of the different alleles in the haplotypes. This varies between haplotypes dependent on the presence of PCR bias in the amplification of specific alleles. In previous publications for haplotypes where there was high correlation (R 2 > 0.9) between the read frequencies observed with the 2 independent PCRs, and a slope of 0.8–1.1 for the line of best fit we have assumed that the read frequency represents relative mRNA transcript expression levels. Based on this it can be observed that the different MHCI haplotypes conform to different patterns with regard to the number of alleles expressed and the expression levels of different constituent alleles. [file TAN-101-458-s006.pdf]

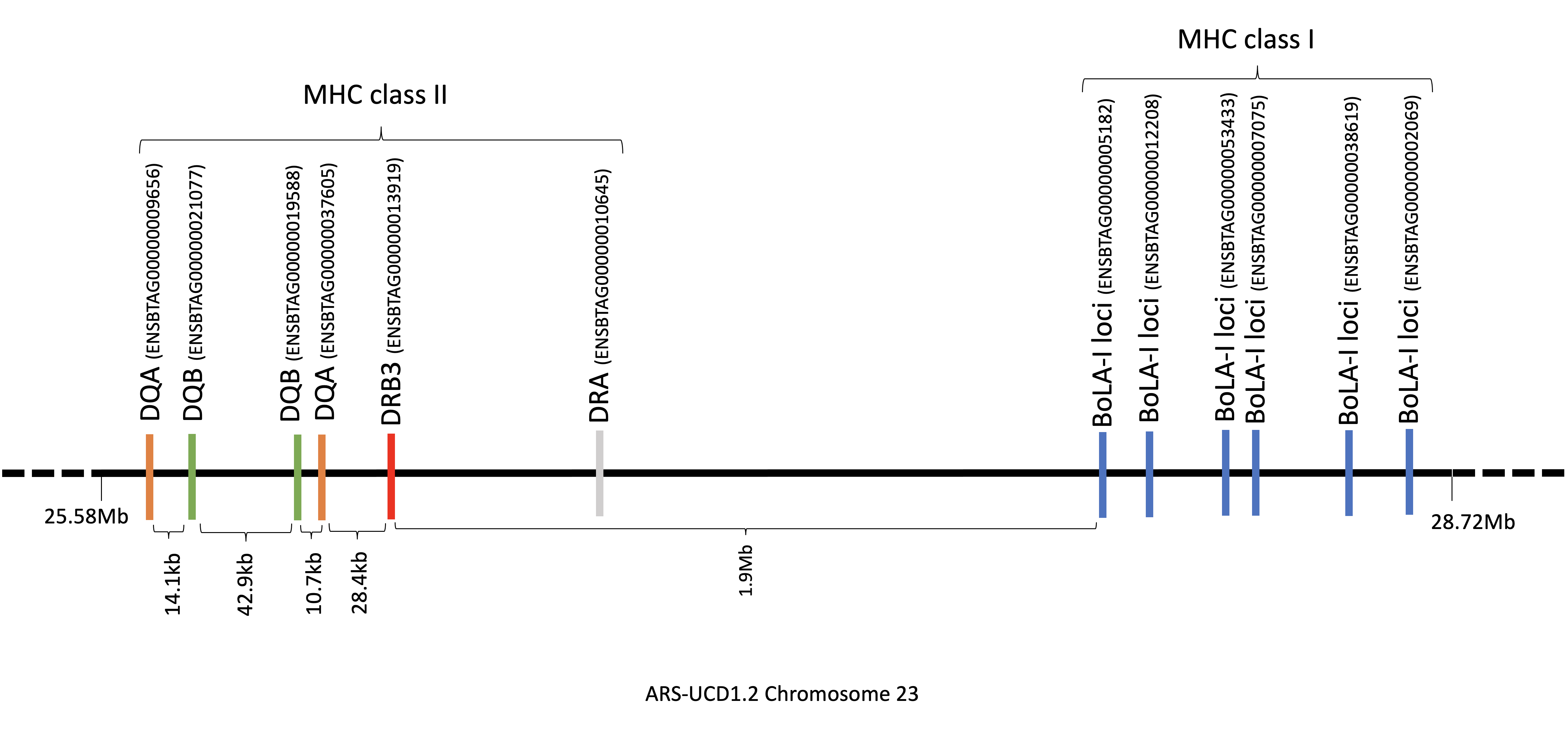

Supplement: Supplementary file 5 — Supplementary Data 5 Genomic organisation of the bovine MHC locus. The bovine MHC locus is located on chromosome 23 at position 25.58–28.72 Mb in the current Ensembl annotation of genome ARS‐UCD1.2. The location of the DQ, DR and BoLA‐I genes are shown (with the Ensembl identifier in brackets). The distances between the gene loci are calculated based on the annotation; paired DQA/DQB loci are in close proximity (10.7 and 14.1 Kb), the distance between the DQ loci is 42.9Kb, the distance between the DRB3 and proximal DQ loci is 28.4Kb and the approximate distance between the DRB3 and MHCI loci is ~1.9 MB. [file TAN-101-458-s007.png]

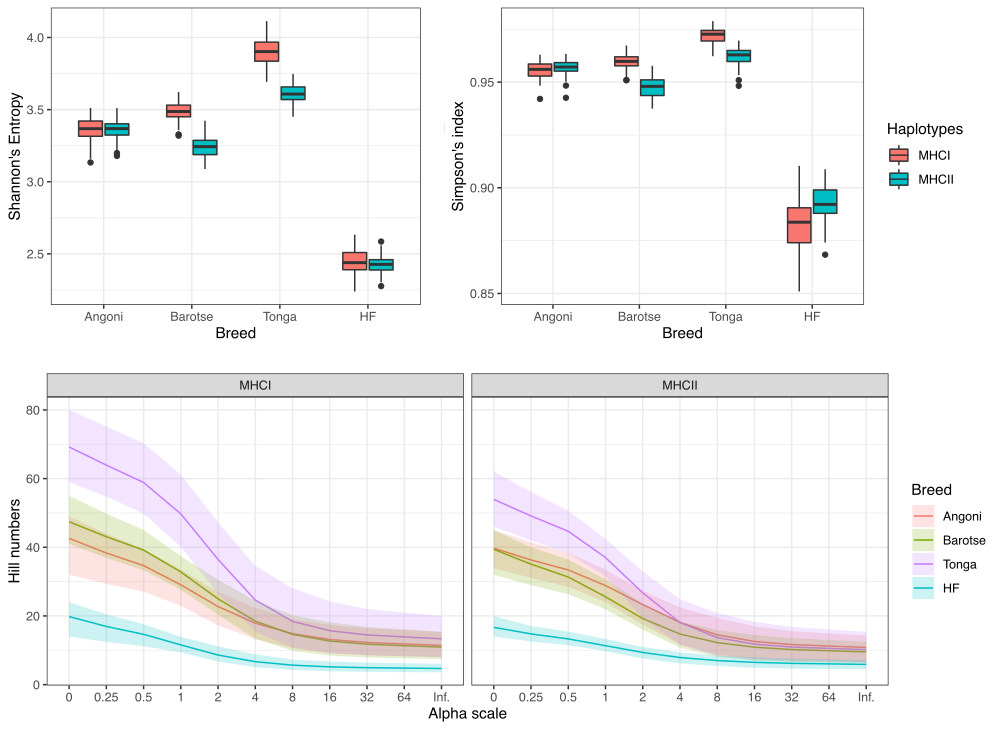

Supplement: Supplementary file 7 — Supplementary Data 7 MHCI and MHCII diversity analysis in Tonga, Barotse, Angoni and Holstein‐Friesian (HF), Shannon entropy, Simpson's diversity index and a profile of Hill numbers using a series of alpha parameter values were used to to analyse a sub‐sample of 100 animals randomly selected for each breed. Shannon's entropy measures the richness (diversity) and evenness (equivalence of frequency) of MHC haplotypes in a population. Decrease in diversity and the evenness in the frequency of MHC haplotypes leads to a reduction in the index value. Simpson's index is a measure that assesses diversity by both the number of MHC haplotypes present in a population as well as the relative frequency of each haplotype. The value ranges between 0 and 1, with higher values reflecting greater diversity. Hill values describe the complexity of a population with the relative effect of the frequency of different haplotypes modified depending on the alpha value (increasing alpha values cause the frequency of MHC haplotypes to have a greater impact; when α = 0 the frequency of haplotypes is not considered so the Hill number is the same as the number of MHC haplotypes in the population). In the graph the value of the Hill number is shown (vertical axis) for a range of α values (horizontal axis). For each breed the mean of the values derived from 100 bootstrap iterations is shown as a solid line, with the range of scores indicated by the ribbon as described in the legend. [file TAN-101-458-s001.png]
